# Towards an asymmetric β-selective addition of azlactones to allenoates

**DOI:** 10.3762/bjoc.20.134

**Published:** 2024-07-04

**Authors:** Behzad Nasiri, Ghaffar Pasdar, Paul Zebrowski, Katharina Röser, David Naderer, Mario Waser

**Affiliations:** 1 Institute of Organic Chemistry, Johannes Kepler University Linz, Altenbergerstrasse 69, 4040 Linz, Austria; 2 Department of Chemistry, Faculty of Science, University of Kurdistan, 66177-15175 Sanandaj, Kurdistan, Iran

**Keywords:** allenoates, amino acids, azlactones, organocatalysis, quaternary ammonium salt catalysis

## Abstract

We herein report the asymmetric organocatalytic addition of azlactones to allenoates. Upon using chiral quaternary ammonium salt catalysts, i.e., Maruoka’s binaphthyl-based spirocyclic ammonium salts, the addition of various azlactones to allenoates proceeds in a β-selective manner with moderate levels of enantioselectivities (up to 83:17 er). Furthermore, the obtained products can be successfully engaged in nucleophilic ring opening reactions, thus giving highly functionalized α-amino acid derivatives.

## Introduction

**Scheme 1:**
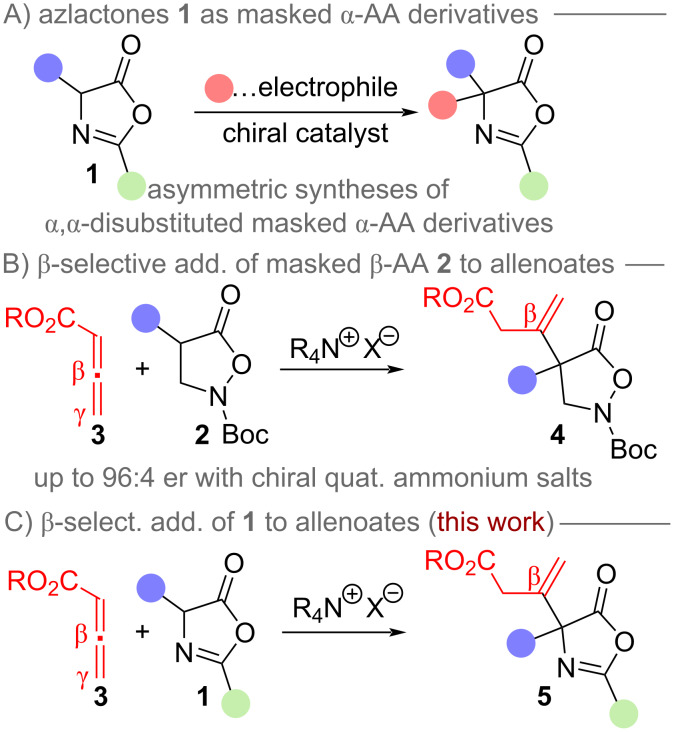
General use of azlactones **1** to access more advanced α-AA derivatives (A), our recently reported ammonium salt-catalyzed β-selective addition of compounds **2** to allenoates **3** (B), and the herein investigated β-selective addition of azlactones **1** to allenoates **3** (C).

The development of asymmetric synthesis routes to access non-natural amino acids has for decades been one of the most heavily investigated tasks in organic synthesis and catalysis-oriented research [1–13]. As a consequence, a broad variety of conceptually orthogonal strategies to access differently functionalized non-natural α-amino acids (α-AA) [2–7] as well as β-amino acids (β-AA) [8–13] have been introduced and there is still considerable interest in the development of new concepts and synthesis approaches. Our group has a longstanding focus on the development of asymmetric organocatalytic methods to access non-natural chiral α- and β-AA [14–19]. Hereby we are especially interested in utilizing simple (prochiral) starting materials and carry out stereoselective α-functionalizations by reacting them with suited C- or heteroatom electrophiles. α-Amino acid-derived azlactones **1** are amongst the most commonly utilized starting materials to access more diverse chiral α,α-disubstituted amino acids (Scheme 1A) [20–22]. More specifically, these compounds can be engaged in a variety of asymmetric α-carbo- and α-heterofunctionalization reactions by utilizing different catalysis strategies [20–22]. We have recently carried out systematic investigations concerning the syntheses of advanced β-AA by means of asymmetric α-carbofunctionalization reactions and during these studies we also realized that the masked β-AA derivatives **2** undergo enantioselective β-addition to allenoates **3** under chiral ammonium salt catalysis (Scheme 1B) [18]. Interestingly, hereby we also found that the use of alternative catalyst systems (i.e., tertiary phosphines) allows for a γ-selective addition of **2** to the allenoate instead, thus resulting in two complementary catalyst-controlled pathways [18]. Based on these previous results, and also the well-documented different reactivity trends of allenoates **3** when using different organocatalysts and activation modes [23–27], we were thus wondering if we could extend this ammonium salt-catalyzed β-selective allenoate functionalization strategy to other amino acid classes. Azlactones **1** have previously been used for γ-selective additions to allenoates under chiral phosphine catalysis [28]. In addition, glycine Schiff base derivatives [29] as well as α-amino acid-based thiazolones [30] have successfully been used for asymmetric β-selective additions to allenoates when using chiral ammonium salt catalysts or chiral organobase catalysts. However, to the best of our knowledge the β-selective asymmetric addition of azlactones **1** to allenoates **3** delivering highly functionalized α,α-disubstituted α-amino acid derivatives **5** has so far not been systematically addressed (for recent other β-selective additions of enolate precursors to allenoates please see references [31–34]). Thus, we now became interested in testing this transformation under asymmetric ammonium salt catalysis [35–38] and the results of these investigations are outlined in this contribution (Scheme 1C).

## Results and Discussion

We started our investigations by testing the quaternary ammonium salt-catalyzed addition of azlactone **1a** to allenoate **3a** (Table 1 gives an overview of the most significant results obtained hereby). First experiments using cinchona alkaloid-based quaternary ammonium salts **A** showed that the expected β-addition product **5a** can be accessed under typical phase-transfer conditions, but with low selectivities and yields only when using these catalysts (Table 1, entries 1–4, other cinchona alkaloid-based ammonium salt derivatives as well as free base cinchona alkaloids were tested too but did not allow for any improvement). Using the established and commercially available Maruoka catalysts **B1** and **B2** [39] next turned out to be more promising (Table 1, entries 5–8). Testing the bis-CF_3_-substitued **B1** first allowed for 75:25 er, but with moderate yield only when carrying out the reaction in toluene in the presence of 3 equiv of K_2_CO_3_ (Table 1, entry 5). Lower amounts of base (Table 1, entry 6) or other solvents, as exemplified for CH_2_Cl_2_ (Table 1, entry 7, similar non-selective results were obtained when using THF), were found to be less-suited however. Testing the 3,4,5-trifluorobenzene-decorated catalyst **B2** with K_2_CO_3_ in toluene next (Table 1, entry 8) allowed for a slightly higher selectivity but still gave only a relatively low yield. Spirobiindane-based salts **C** emerged as promising alternative for quaternary ammonium salt scaffolds recently [40–41] and were also the catalysts of choice in our recently developed β-selective allenoate addition of isoxazolidinones **2** (compare with Scheme 1B [18]). Unfortunately, these catalysts were found to be less-suited for our azlactone protocol, as exemplified for derivative **C1** (Table 1, entry 9). Accordingly, we carried out our final optimization using Maruoka’s catalyst **B2** (Table 1, entries 10–14). By testing different bases and lower temperatures as well as lower catalyst loadings we identified the use of 3 equiv Cs_2_CO_3_ in toluene (0.05 M) at room temperature as the best-suited conditions (Table 1, entry 13), allowing for the synthesis of **5a** in moderate yield (61%) and enantioselectivity (81:19 er).

**Table 1:** Optimization of the addition of azlactone **1a** to allenoate **3a**^a^.

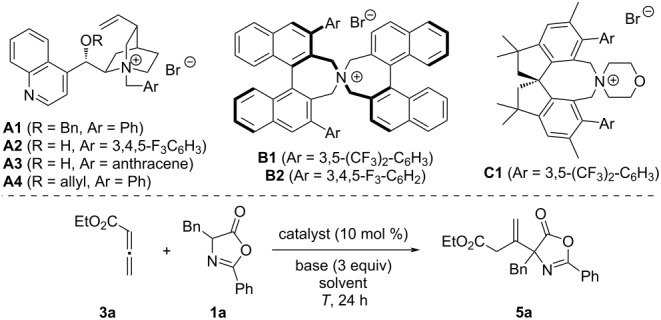						

Entry	Cat.	Base	Solvent	*T* [°C]	Yield^b^	er^c^

1	**A1**	K_2_CO_3_	toluene	25	41	58:42
2	**A2**	K_2_CO_3_	toluene	25	45	60:40
3	**A3**	K_2_CO_3_	toluene	25	40	58:42
4	**A4**	K_2_CO_3_	toluene	25	45	60:40
5	**B1**	K_2_CO_3_	toluene	25	55	75:25
6	**B1**	K_2_CO_3_ (1 equiv)	toluene	25	20	72:28
7	**B1**	K_2_CO_3_	CH_2_Cl_2_	25	33	51:49
8	**B2**	K_2_CO_3_	toluene	25	50	80:20
9	**C1**	K_2_CO_3_	toluene	25	40	68:32
10	**B2**	K_2_CO_3_	toluene	0	45	80:20
11	**B2** (5%)	K_2_CO_3_	toluene	0	41	77:23
12	**B2**	K_3_PO_4_	toluene	25	55	81:19
13	**B2**	Cs_2_CO_3_	toluene	25	61	81:19
14	**B2**	Cs_2_CO_3_	toluene (0.1 M)	25	75	73:27

^a^Unless otherwise stated, all reactions were carried out by stirring **1a** (0.1 mmol), the allenoate (2 equiv), the indicated base and the catalyst, in the given solvent (0.05 M based on **1a**) at the given temperature for 24 h. ^b^Isolated yield. ^c^Determined by HPLC using a chiral stationary phase, (−)-**5a** was obtained as the major enantiomer when using the (*R*,*R*)-configurated catalysts **B**.

**Scheme 2:**
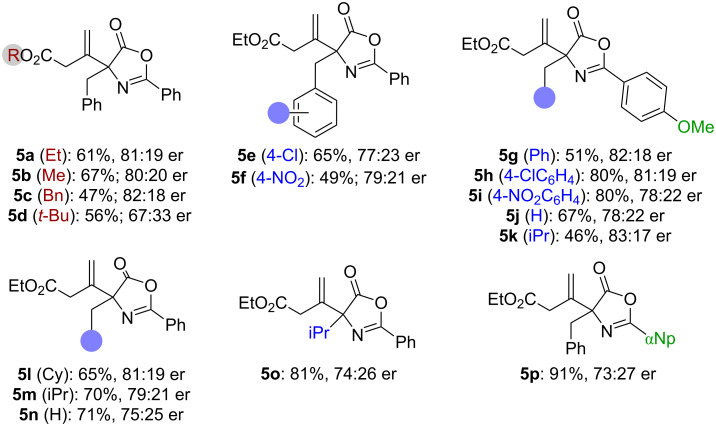
Application scope (conditions as detailed in Table 1, entry 13).

With optimized conditions for the synthesis of enantioenriched (−)-**5a** at hand, we next investigated the generality of this protocol. As outlined in Scheme 2, differently substituted allenoates were reasonably well tolerated (see products **5a**–**d**), albeit some erosion in enantioselectivity was observed when using a *tert*-butyl ester containing allenoate (product **5d**). Various α-arylmethyl-substituted azlactones **1** performed similarly as compared to the parent system **1a** (products **5e**–**i**), and analogous α-alkyl-substituted derivatives were reasonably well accepted too (**5j**–**o**). When varying the aryl substituent in position **2** of the oxazolone core (compare products **5a**, **5g**, and **5p**) we found that increasing the steric bulk (**5p**) leads to a somewhat lower enantioselectivity, while the methoxy-substituent does not have a strong impact on the yield. It should, however, be stated that some of the methoxy-containing products, i.e., the α-alkyl-substituted **5j** and **5k** tend to undergo partial nucleophilic ring opening by residual water during column chromatography. Unfortunately, attempts to assign the absolute configuration of products **5** failed, as we have not been able to obtain any crystals suited for single crystal X-ray diffraction analysis.

**Scheme 3:**
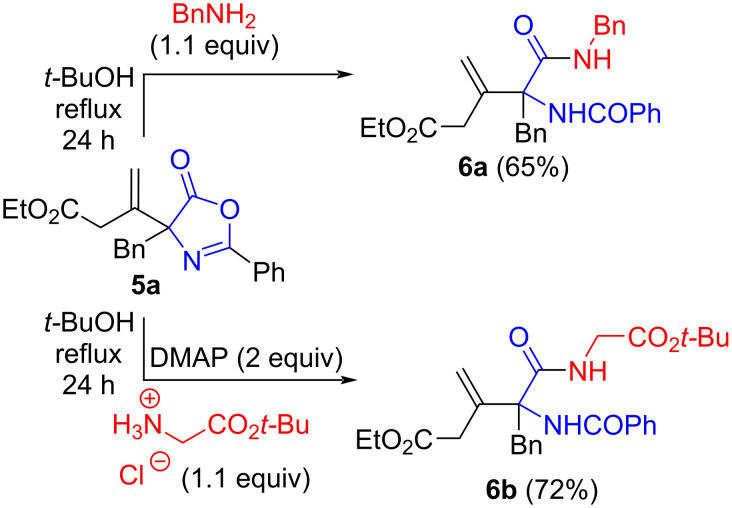
Azlactone opening reactions.

Finally, we also tested the suitability of products **5** to access acyclic α-AA derivatives by means of nucleophilic azlactone-opening reactions. Gratifyingly primary amines can be easily utilized under reflux conditions to access the amide derivatives **6a** and **6b** straightforwardly (Scheme 3), thus demonstrating the versatility of compounds **5** to access more complex acyclic α-AA derivatives in a straightforward manner.

## Conclusion

The development of novel catalytic methods for the asymmetric synthesis of non-natural amino acid derivatives is a contemporary task and we herein introduce an organocatalytic protocol for the β-selective addition of various azlactones **1** to allenoates **3**. Upon using Maruoka’s spirocyclic binaphthyl-based quaternary ammonium salts **B** as catalysts this transformation can be achieved with enantioselectivities up to 83:17 er. Furthermore, the herein accessed cyclic products **5** could be successfully engaged in ring-opening reactions with different amines, thus giving access to the acyclic α-amino acid-based amides **6** straightforwardly.

## Experimental

### General details

^1^H and ^13^C NMR spectra were recorded on a Bruker Avance III 300 MHz spectrometer with a broad band observe probe. All NMR spectra were referenced on the solvent residual peak (CDCl_3_: δ 7.26 ppm for ^1^H NMR and δ 77.16 ppm for ^13^C NMR). NMR data are reported as follows: chemical shift (δ ppm), multiplicity (s = singlet, d = doublet, t = triplet, q = quartet, m = multiplet, dd = doublet of doublet), coupling constants (Hz), relative integration value. High-resolution mass spectra were obtained using a Thermo Fisher Scientific LTQ Orbitrap XL spectrometer with an Ion Max API source and analyses were made in the positive ionization mode if not otherwise stated. Infrared (IR) spectra were recorded on a Bruker Alpha II FTIR spectrometer with diamond ATR-module using the OPUS software package and are reported in terms of frequency of absorption (cm^−1^). HPLC was performed using a Shimadzu Prominence system with a diode array detector with a CHIRALPAK AD-H, CHIRAL ART Amylose-SA, (250 × 4.6 mm, 5 µm) chiral stationary phase. Optical rotations were recorded on a Schmidt + Haensch Polarimeter Model UniPol L1000 at 589 nm ([α]_D_ values are listed in deg/(dm(g/cm^3^)); concentration *c* is given in g/100 mL).

Unless otherwise stated, all chemicals were purchased from commercial suppliers and used without further purification. Dry solvents were obtained from an MBraun-SPS-800 solvent purification system. All reactions were carried out under argon atmosphere unless stated otherwise. Azlactones **1** and allenoates **3** were synthesized according to previously published procedures [18,42–44].

### General procedure

An oven-dried Schlenk tube equipped with a stirring bar was charged with azlactone **1** (0.05–0.1 mmol), catalyst **B2** (10 mol % related to **1**), and Cs_2_CO_3_ (3 equiv). Then the respective allenoate **3** (2 equiv) and toluene (0.05 M with respect to **1**) were added and the mixture was stirred at room temperature for 24 h (Ar atmosphere). The crude product was passed through a short column of silicagel (rinsed with DCM and EtOAc), concentrated under reduced pressure, and subsequently purified by preparative TLC (silica gel, heptanes/EtOAc 4:1) to obtain the products **2** in the given yields and enantiopurities.

**Details for the parent compound 5a** (details for the other targets can be found in Supporting Information File 1). Obtained as a colorless oil in 61% yield (81:19 er) on 0.1 mmol scale. [α]_D_^22^ = −11.4 (*c* 1.1, CHCl_3_); ^1^H NMR (300 MHz, CDCl_3_, 298.0 K) δ/ppm = 7.85 (dd, *J* = 8.6, 1.4 Hz, 2H), 7.54 (t, *J* = 7.4 Hz, 1H), 7.43 (t, *J* = 7.53 Hz, 2H), 7.24–7.11 (m, 5H), 5.79 (s, 1H), 5.37 (s, 1H), 4.14–3.90 (m, 2H), 3.52–3.16 (m, 4H), 1.15 (t, *J* = 7.1 Hz, 3H); ^13^C NMR (75 MHz, CDCl_3_, 298.0 K) δ/ppm = 177.4, 171.0, 160.3, 139.1, 133.8, 132.6, 130.5, 128.6, 128.0, 127.8, 127.3, 125.6, 118.1, 75.9, 60.9, 44.9, 39.3, 13.9; IR (neat): 3080, 3070, 2917, 1815, 1732, 1656, 1480, 1175, 1093, 1059, 1030, 974, 893, 694 cm^−1^; HRESIMS *m*/*z*: [C_22_H_21_NO_4_ + H]^+^ calcd for 364.1543; found, 364.1554; HPLC: (Chiralpak SA, eluent: *n*-hexane/iPrOH = 100:2, 0.5 mL·min^−1^, 20 °C, λ = 254 nm) retention times: *t*_major_ = 16.15 min, *t*_minor_ = 17.00 min.

## Supporting Information

File 1 Full experimental and analytical details and copies of NMR spectra and HPLC traces.

## Data Availability

All data that supports the findings of this study is available in the published article and/or the supporting information to this article.
