# Supplementary material for: Towards an asymmetric β-selective addition of azlactones to allenoates
Source: Beilstein J Org Chem. 2024 Jul 4;20:1504–9. doi: 10.3762/bjoc.20.134 (PMC11228823; doi:10.3762/bjoc.20.134)
Supplement: File 1 — Full experimental and analytical details and copies of NMR spectra and HPLC traces. [file Beilstein_J_Org_Chem-20-1504-s001.pdf]

BEILSTEIN JOURNAL OF ORGANIC CHEMISTRY

Supporting Information

for

Towards an asymmetric β-selective addition of azlactones to allenoates

Behzad Nasiri, Ghaffar Pasdar, Paul Zebrowski, Katharina Röser, David Naderer and Mario Waser

Beilstein J. Org. Chem. **2024,** *20,* 1504–1509. doi:10.3762/bjoc.20.134

Full experimental and analytical details and copies of NMR spectra and HPLC traces

License and Terms: This is a supporting information file under the terms of the Creative Commons Attribution License (https://creativecommons.org/licenses/by/4.0). Please note that the reuse, redistribution and reproduction in particular requires that the author(s) and source are credited and that individual graphics may be subject to special legal provisions.
The license is subject to the *Beilstein Journal of Organic Chemistry* terms and conditions: (https://www.beilstein-journals.org/bjoc/terms)

Table of Contents

Table of Contents S1

1. General information S2

2. Asymmetric protocol S3

   General procedure S3

   Characterization of the products S3

3. Ring opening reactions S14

4. NMR Spectra S16

5. HPLC Chromatograms S50

1. General information

^{1}H-,^{13}C- spectra were recorded on a Bruker Avance III 300 MHz spectrometer with a broad band observe probe. All NMR spectra were referenced on the solvent residual peak ($CDCl_3$: δ 7.26 ppm for ^{1}H NMR and δ 77.16 ppm for ^{13}C NMR). NMR data are reported as follows: chemical shift (δ ppm), multiplicity (s = singlet, d = doublet, t = triplet, q = quartet, m = multiplet, dd = doublet of doublet), coupling constants (Hz).

High-resolution mass spectra were obtained using a Thermo Fisher Scientific LTQ Orbitrap XL with an Ion Max API Source and analyses were made in the positive ionization mode if not otherwise stated.

Infrared (IR) spectra were recorded on a Bruker Alpha II FTIR spectrometer with diamond ATR-module using OPUS software package and are reported in terms of frequency of absorption (cm^{-1}).

HPLC was performed using a Shimadzu Prominence system with a diode array detector with a CHIRALPAK AD-H, CHIRAL ART Amylose-SA, (250 × 4.6 mm, 5 µm) chiral stationary phase. Optical rotations were recorded on a Schmidt + Haensch Polarimeter Model UniPol L1000 at 589 nm ($[\alpha]_D$ values are listed in deg/(dm(g/cm^3)); concentration c is given in g/100 mL).

Unless otherwise stated, all chemicals were purchased from commercial suppliers and used without further purification.

Azlactones[1] and allenoates[2] were synthesized according to known procedures.

Dry solvents were obtained from an MBraun-SPS-800 solvent purification system. All reactions were carried out under argon atmosphere unless stated otherwise.

1) a) Macovei, C.; Vicennati, P.; Quinton, J.; Nevers, M.-C.; Volland, H.; Créminon, C.; Taran, F. *Chem. Commun*. **2012**, *48*, 4411-4413; b) de Mello, A. C.; Momo, P. B.; Burtoloso, A. C. B.; Amarante, G. W. *J. Org. Chem*. **2018**, *83*, 11399-11406; c) Žabka, M.; Kocian, A.; Bilka, S.; Andrejčák, S.; Šebesta, R. *Eur. J. Org. Chem*. **2019**, 6077-6087.
2) Zebrowski, P.; Röser, K.; Chrenko, D.; Pospíšil, J.; Waser, M. *Synthesis* **2023**, *55*, 1706-1713.

2. Asymmetric Protocol

General procedure

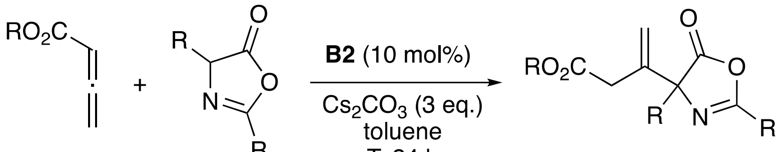

An oven-dried Schlenk tube equipped with a stirring bar was charged with the azlactone **1** (0.05–0.1 mmol), catalyst **B2** (10 mol % related to **1**), and Cs_2CO_3 (3 equiv). Then the respective allenoate **3** (2 equiv) and toluene (0.05 M with respect to **1**) were added and the mixture was stirred at room temperature for 24 h (Ar atmosphere). The crude product was passed through a short column of silicagel (rinsed with DCM and EtOAc), concentrated under reduced pressure, and subsequently purified by preparative TLC (silica gel, heptanes/EtOAc = 4:1) to obtain the products **2** in the given yields and enantiopurities.

Characterization of the products

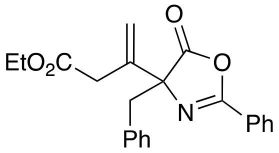

5a

Compound 5a: Obtained as a colorless oil in 61% yield with er = 81:19.

$[\alpha]_D^{22}$ (c = 1.1, $CHCl_3$) = –11.4°.

^{1}H NMR (300 MHz, δ, $CDCl_3$, 298 K): 7.85 (2H, dd, *J* = 8.6, 1.4 Hz), 7.54 (1H, t, *J* = 7.4 Hz), 7.43 (2H, t, *J* = 7.53 Hz), 7.11-7.24 (5H, m), 5.79 (1H, s), 5.37 (1H, s), 3.90-4.14 (2H, m), 3.16-3.52 (4H, m), 1.15 (3H, t, *J* = 7.1 Hz).

^{13}C{1H} NMR (75 MHz, δ, $CDCl_3$, 298 K): 177.4, 171.0, 160.3, 139.1, 133.8, 132.6, 130.5, 128.6, 128.0, 127.8, 127.3, 125.6, 118.1, 75.9, 60.9, 44.9, 39.3, 13.9.

IR (neat): 3080, 3070, 2917, 1815, 1732, 1656, 1480, 1175, 1093, 1059, 1030, 974, 893, 694 cm^{-1}.

HRMS for $C_{22}H_{21}NO_4$ [M+H]+: m/z calcd: 364.1543, found: 364.1554.

HPLC (Chiralpak SA, eluent: *n*-hexane:iPrOH = 100/2, 0.5 mL· min^{-1}, 20 °C, λ = 254 nm) retention times: t_{major} = 16.15 min, t_{minor} = 17.00 min.

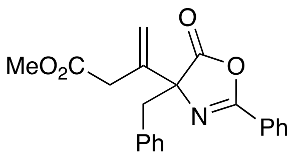

5b

Compound 5b: Obtained as a colorless oil in 67% yield with er = 80:20.

$[\alpha]_D^{22}$ (c = 0.93, $CHCl_3$) = –13.8°.

^{1}H NMR (300 MHz, δ, $CDCl_3$, 298 K): 7.81-7.88 (2H, m), 7.49-7.58 (1H, m), 7.38-7.48 (2H, m), 7.13-7.23 (5H, m), 5.78 (1H, s), 5.36 (1H, s), 3.56 (3H, s), 3.20-3.48 (4H, m).

^{13}C{1H} NMR (75 MHz, δ, $CDCl_3$, 298 K): 177.4, 171.4, 160.3 138.9, 133.7, 132.6, 130.4, 128.6, 128.0, 127.8, 127.3, 125.5, 118.2, 75.9, 51.9, 44.8, 39.1.

IR (neat): 3046, 2940, 1814, 1736, 1656, 1451, 1167, 1093, 972, 892, 779, 694 cm^{-1}.

HRMS for $C_{21}H_{19}NO_4$[M+H]+: m/z calcd: 350.1387, found: 350.1377.

HPLC (Chiralpak AD-H, eluent: *n*-hexane:iPrOH = 100/1, 0.5 mL· min^{-1}, 20 °C, λ = 254 nm) retention times: t_{major} = 52.66 min, t_{minor}= 56.59 min.

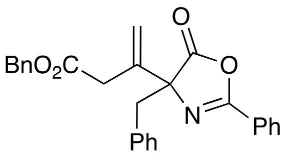

5c

Compound 5c: Obtained as a colorless oil in 47% yield with er = 82:18.

$[\alpha]_D^{22}$ (c = 1.05, $CHCl_3$) = –21.4°.

^{1}H NMR (300 MHz, δ, $CDCl_3$, 298 K): 7.76-7.83 (2H, m), 7.49-7.57 (1H, m), 7.35-7.45 (2H, m), 7.23-7.35 (5H, m), 7.12-7.21 (5H, m), 5.79 (1H, s), 5.37 (1H, s), 5.01 (1H, d, *J* = 0.57 Hz), 3.26-3.51 (4H, m).

^{13}C{1H} NMR (75 MHz, δ, $CDCl_3$, 298 K): 177.4, 170.9, 160.3, 138.9, 135.5, 133.7, 132.6, 130.4, 128.6, 128.5, 128.5, 128.3, 128.0, 127.8, 127.3, 125.5, 118.3, 75.9, 66.6, 44.9, 39.2.

IR (neat): 3057, 2944, 1820, 1732, 1656, 1155, 1093, 1059, 971, 746, 693 cm^{-1}.

HRMS for $C_{27}H_{23}NO_4$ [M+H]+: m/z calcd: 426.1700, found: 426.1690.

HPLC (Chiralpak AD-H, eluent: *n*-hexane:iPrOH = 100/3, 0.5 mL· min^{-1}, 20 °C, λ = 254 nm) retention times: t_{major} = 37.04 min, t_{minor} = 41.42 min.

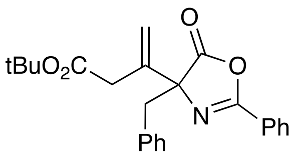

5d

Compound 5d: Obtained as a colorless oil in 56% yield with er = 67:33.

$[\alpha]_D^{22}$ (c = 1.1, $CHCl_3$) = +7.9°.

^{1}H NMR (300 MHz, δ, $CDCl_3$, 298 K): 7.82-7.88 (2H, m), 7.49-7.56 (1H, m), 7.38-7.46 (2H), 7.10-7.24 (5H, m), 5.76 (1H, s), 5.34 (1H, s), 3.14-3.42 (4H, m), 1.35 (9H, s).

^{13}C{1H} NMR (75 MHz, δ, $CDCl_3$, 298 K): 177.5, 170.3, 160.1, 139.5, 132.5, 130.4, 128.5, 128.0, 127.2, 125.7, 117.8, 81.0, 76.0, 44.9, 40.3, 27.8.

IR (neat): 3040, 2932, 1814, 1718, 1655, 1148, 1096, 1058, 969, 699 cm^{-1}.

HRMS for $C_{24}H_{25}NO_4$ [M+H]+: m/z calcd: 392.1856, found: 392.1860.

HPLC (Chiralpak AD-H, eluent: *n*-hexane:iPrOH = 100/3, 0.5 mL· min^{-1}, 20 °C, λ = 254 nm) retention times: t_{major} = 13.73 min, t_{minor} = 14.74 min.

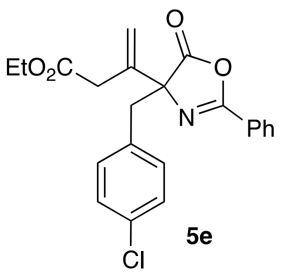

5e

Compound 5e: Obtained as a yellow oil in 65% yield with er = 77:23.

$[\alpha]_D^{22}$ (c = 0.97, $CHCl_3$) = –20.1°.

^{1}H NMR (300 MHz, δ, $CDCl_3$, 298 K): 8.06 (2H, d, *J* = 8.76 Hz), 7.82-7.90 (1H, m), 7.52-7.60 (2H, m), 7.35-7.50 (4H, m), 5.79 (1H, s), 5.37 (1H, s), 5.01 (1H, d, *J* = 0.57 Hz), 3.98-4.22 (2H, m), 3.26-3.51 (4H, m), 1.15 (3H, t, *J* = 7.14 Hz).

^{13}C{1H} NMR (75 MHz, δ, $CDCl_3$, 298 K): 177.2, 170.9, 160.5, 138.9, 133.3, 132.8, 132.3, 131.7, 128.7, 128.2, 127.8, 125.4, 118.3, 75.6, 61.0, 44.1, 39.3, 13.9.

IR (neat): 3055, 2923, 1810, 1731, 1657, 1509, 1480, 1158, 1095, 1016, 803, 712 cm^{-1}.

HRMS for $C_{22}H_{20}ClNO_4$ [M+H]+: m/z calcd: 398.1154, found: 398.1149.

HPLC (Chiralpak AD-H, eluent: *n*-hexane:iPrOH = 150/1, 0.5 mL· min^{-1}, 20 °C, λ = 254 nm) retention times: t_{minor}= 39.63 min, t_{major} = 41.52 min.

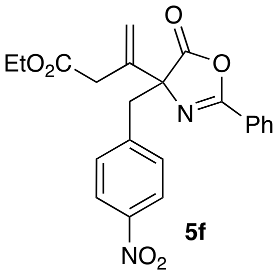

5f

Compound 5f: Obtained as a yellow oil in 49% yield with er = 79:21.

$[\alpha]_D^{22}$ (c = 0.98, $CHCl_3$) = –13.7°.

^{1}H NMR (300 MHz, δ, $CDCl_3$, 298 K): 8.06 (2H, d, *J* = 8.76 Hz), 7.82-7.90 (2H, m), 7.53-7.61 (1H, m), 7.35-7.50 (4H, m), 5.78 (1H, s), 5.39 (1H, s), 3.93-4.09 (2H, m), 3.18-3.56 (4H, m), 1.15 (3H, t, *J* = 7.14 Hz).

^{13}C{1H} NMR (75 MHz, δ, $CDCl_3$, 298 K): 176.9, 170.8, 160.8, 147.3, 141.5, 138.6, 133.1, 131.4, 128.8, 127.8, 125.0, 123.2, 118.7, 75.2, 61.0, 44.3, 39.2, 13.9.

IR (neat): 3053, 2919, 1812, 1731, 1657, 1513, 1473, 1345, 1257, 1180, 1110, 1028, 833, 699, 523 cm^{-1}.

HRMS for $C_{22}H_{20}N_2O_6$[M+H]+: m/z calcd: 409.1394, found: 409.1398.

HPLC (Chiralpak AD-H, eluent: *n*-hexane:iPrOH = 100/3, 0.5 mL· min^{-1}, 20 °C, λ = 254 nm) retention times: t_{minor} = 52.67 min, t_{major} = 55.12 min.

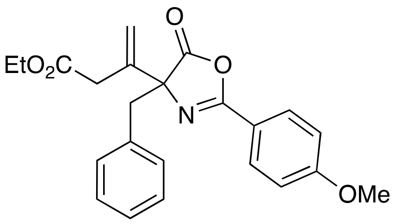

5g

Compound 5g: Obtained as a colorless oil in 51% yield with er = 82:18.

$[\alpha]_D^{22}$ (c = 1.00, $CHCl_3$) = –36.9°.

^{1}H NMR (300 MHz, δ, $CDCl_3$, 298 K): 7.79 (2H, d, *J* = 8.9 Hz), 7.13-7.22 (5H, m), 6.92 (2H, d, *J* = 8.9 Hz), 5.77 (1H, s), 5.35 (1H, s), 4.01 (2H, m), 3.86 (3H, s), 3.18-3.45 (4H, m), 1.15 (3H, t, *J* = 7.1 Hz).

^{13}C{1H} NMR (75 MHz, δ, $CDCl_3$, 298 K): 177.6, 171.0, 163.0, 159.9, 139.3, 133.9, 130.4, 129.7, 128.0, 127.2, 117.9, 117.9, 114.0, 75.8, 60.8, 55.4, 44.9, 39.4, 13.9.

IR (neat): 3020, 3048, 2943, 1813, 1731, 1654, 1607, 1511, 1256, 1170, 1093, 1028, 974, 840, 700 cm^{-1}.

HRMS for $C_{23}H_{23}NO_5$ [M+H]+: m/z calcd: 394.1649, found: 394.1665.

HPLC (Chiralpak AD-H, eluent: *n*-hexane:iPrOH = 100/3, 0.5 mL· min^{-1}, 20 °C, λ = 254 nm) retention times: t_{major} = 38.93 min, t_{minor} = 48.14 min.

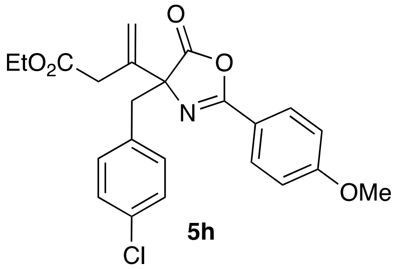

5h

Compound 5h: Obtained as a colorless oil in 80% yield with er = 81:19.

$[\alpha]_D^{22}$ (c = 0.90, $CHCl_3$) = –73°.

^{1}H NMR (300 MHz, δ, $CDCl_3$, 298 K): 7.81 (2H, d, *J* = 8.9 Hz), 7.09-7.19 (4H, m), 6.94 (2H, d, *J* = 8.9 Hz), 5.35 (1H, s), 5.75 (1H, s), 3.91-4.10 (2H, m), 3.87 (3H, s), 3.15-3.44 (4H, m), 1.16 (3H, t, *J* = 7.1 Hz).

^{13}C{1H} NMR (75 MHz, δ, $CDCl_3$, 298 K): 177.4, 170.9, 163.2, 160.2, 139.2, 133.2, 132.5, 131.8, 129.7, 128.2, 118.1, 117.7, 114.1, 75.5, 60.9, 55.4, 44.1, 39.3, 13.9.

IR (neat): 3010, 2727, 1813, 1731, 1653, 1607, 1511, 1034, 1257, 1171, 1068, 1027, 975, 838, 742, 604, 511 cm^{-1}.

HRMS for $C_{23}H_{22}ClNO_5$ [M+H]+: m/z calcd: 428.1259, found: 428.1275.

HPLC (Chiralpak AD-H, eluent: *n*-hexane:iPrOH = 100/3, 0.5 mL· min^{-1}, 20 °C, λ = 254 nm) retention times: t_{major} = 40.07 min, t_{minor} = 42.30 min.

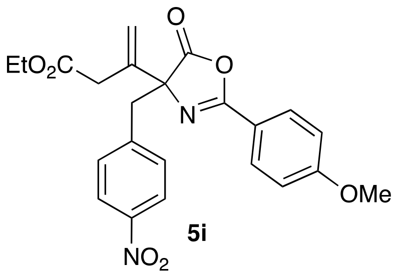

5i

Compound 5i: obtained as a pale-yellow oil in 80% yield with er = 78:22.

$[\alpha]_D^{22}$ (c = 1.1, $CHCl_3$) = –22°.

^{1}H NMR (300 MHz, δ, $CDCl_3$, 298 K): 8.06 (2H, d, *J* = 8.76 Hz), 7.80 (2H, d, *J* = 8.97 Hz), 7.39 (2H, d, *J* = 8.76 Hz), 6.93 (2H, d, *J* = 8.97 Hz), 5.77 (1H, s), 5.38 (1H, s), 3.95-4.07 (2H, m), 3.88 (3H, s), 3.18-3.52 (4H, m), 1.16 (3H, t, *J* = 7.1 Hz).

^{13}C{1H} NMR (75 MHz, δ, $CDCl_3$, 298 K): 177.1, 170.8, 163.4, 160.5, 147.2, 141.7, 138.9, 131.4, 129.7, 123.1, 118.5, 117.3, 114.2, 75.1, 61.0, 55.4, 44.3, 39.3, 13.9.

IR (neat): 3040, 2946, 2765, 1814, 1727, 1656, 1606, 1511, 1341, 1299, 1254, 1171, 1084, 1033, 840, 713, 498 cm^{-1}.

HRMS for $C_{23}H_{22}N_2O_7$ [M+H]+: m/z calcd: 439.1500., found: 439.1496.

HPLC (Chiralpak AD-H, eluent: *n*-hexane:iPrOH = 100/3, 0.8 mL· min^{-1}, 20 °C, λ = 254 nm) retention times: t_{major} = 55.99 min, t_{minor} = 59.93 min.

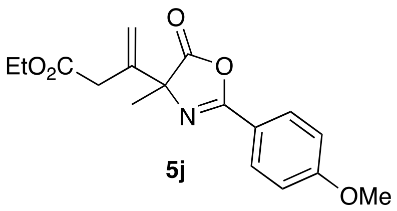

5j

Compound 5j: Obtained as a colorless oil in 67% yield with er = 78:22.

$[\alpha]_D^{22}$ (c = 0.97, $CHCl_3$) = +19.7°.

^{1}H NMR (300 MHz, δ, $CDCl_3$, 298 K): 7.96 (2H, d, *J* = 8.9 Hz), 7.01 (2H, d, *J* = 9.0 Hz), 5.60 (1H, s), 5.29 (1H, s), 3.97-4.10 (2H, m), 3.90 (3H, s), 3.37 (1H, dd, *J* = 16.2, 0.9 Hz), 3.17 (1H, dd, *J* = 16.2, 0.8 Hz), 1.17 (3H, t, *J* = 7.14 Hz).

^{13}C{1H} NMR (75 MHz, δ, $CDCl_3$, 298 K): 178.7, 170.9, 163.3, 160.3, 139.9, 129.9, 118.0, 117.5, 114.2, 71.0, 60.9, 55.5, 38.8, 25.2, 13.9.

IR (neat): 3080, 2911, 1973, 1750, 1814, 1733, 1655, 1449, 1157, 1031, 928, 692 cm^{-1}.

HRMS for $C_{17}H_{19}NO_5$ [M+H]+: m/z calcd: 288.1230, found: 288.1238.

HPLC (Chiralpak AD-H, eluent: *n*-hexane:iPrOH = 100/3, 0.5 mL· min^{-1}, 20 °C, λ = 254 nm) retention times: t_{major} = 31.90 min, t_{minor} = 45.37 min.

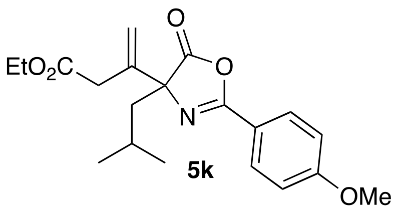

5k

Compound 5k: Obtained as a colorless oil in 46% yield with er = 83:17.

$[\alpha]_D^{22}$ (c = 0.91 $CHCl_3$) = +5.4°.

^{1}H NMR (300 MHz, δ, $CDCl_3$, 298 K): 7.95 (2H, d, *J* = 8.9 Hz), 6.97 (2H, d, *J* = 8.9 Hz), 5.57 (1H, s), 5.21 (1H, s), 3.90-4.09 (2H, m), 3.87 (3H, s), 3.35 (1H, d, *J* = 16.4 Hz), 3.16 (1H, d, *J* = 16.2 Hz), 2.08 (1H, dd, *J* = 13.8, 5.1 Hz), 1.83 (1H, dd, *J* = 13.8, 7.4 Hz), 1.58-1.73 (1H, m), 1.13 (3H, t, *J* =7.1 Hz), 0.82-0.91 (6H, m).

^{13}C{1H} NMR (75 MHz, δ, $CDCl_3$, 298 K): 178.9, 171.0, 163.2, 159.8, 140.1, 129.8, 118.2, 117.1, 114.1, 74.4, 60.8, 55.5, 47.3, 39.0, 24.9, 24.2, 23.0, 13.9.

IR (neat): 3045, 2955, 1810, 1733, 1654, 1607, 1511, 1305, 1256, 1170, 1028, 970, 885, 840, 734, 604 cm^{-1}.

HRMS for $C_{20}H_{25}NO_5$ [M+H]+: m/z calcd: 360.1805, found: 360.1806.

HPLC (Chiralpak AD-H, eluent: *n*-hexane:iPrOH = 100/3, 0.5 mL· min^{-1}, 20 °C, λ = 254 nm) retention times: t_{major} = 23.55 min, t_{minor}= 31.88 min.

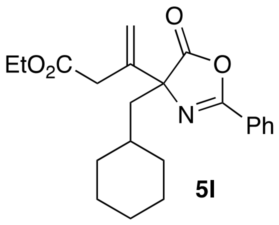

5l

Compound 5l: Obtained as a colorless oil 65% yield with er = 81:19.

$[\alpha]_D^{22}$ (c = 0.94, $CHCl_3$) = +6.9°.

^{1}H NMR (300 MHz, δ, $CDCl_3$, 298 K): 7.89-7.98 (2H, m), 7.47-7.54 (1H, m), 7.37-7.46 (2H, m), 5.51 (1H, s), 5.16 (1H, s), 3.81-4.01 (2H, m), 2.96-3.35 (2H, m), 1.99 (1H, dd, *J* = 14.2, 4.8 Hz), 1.76 (1H, dd, *J* = 7.0, 14 Hz), 1.38-1.71 (13H, m), 1.05 (1H, t, *J* = 7.1 Hz).

^{13}C{1H} NMR (75 MHz, δ, $CDCl_3$, 298 K): 178.7, 171.0, 160.1, 139.7, 132.7, 128.7, 127.9, 125.9, 117.3, 74.4, 60.8, 46.0, 39.0, 34.6, 34.1, 33.6, 26.1, 26.1, 26.0, 13.9.

IR (neat): 3040, 2922, 2852, 1816, 1735, 1656, 1449, 1320, 1156, 1031, 972, 693 cm^{-1}.

HRMS for $C_{23}H_{27}NO_4$ [M+H]+: m/z calcd: 370.2013, found: 370.2014.

HPLC (Chiralpak AD-H, eluent: *n*-hexane:iPrOH = 100/3, 0.5 mL· min^{-1}, 20 °C, λ = 254 nm) retention times: t_{minor} = 13.73 min, t_{major} = 15.60 min.

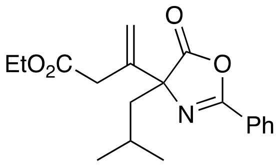

5m

Compound 5m: Obtained as a colorless oil in 70% yield with er = 79:21.

$[\alpha]_D^{22}$ (c = 0.95, $CHCl_3$) = +19.7°.

^{1}H NMR (300 MHz, δ, $CDCl_3$, 298 K): (2H, m), 7.56-764 (1H, m), 7.46-7.55 (2H, m), 5.62 (1H, s), 5.26 (1H, s), 3.90-4.06 (2H, m), 3.38 (1H, dd, *J* = 16.4, 0.9 Hz), 3.20 (1H, d, *J* = 16.3 Hz), 2.13 (1H,

dd, J = 13.9, 5.0 Hz), 1.87 (1H, dd, J = 13.9, 7.4 Hz), 1.61-1.77 (1H, m), 1.15 (3H, t, J = 7.14 Hz), 0.81-0.94 (6H, m).

$^{13}C\{1H\}$ NMR (75 MHz, δ, $CDCl_3$, 298 K): 178.7, 170.9, 160.1, 139.8, 132.7, 128.7, 128.4, 127.9, 117.3, 74.5, 60.8, 47.3, 38.9, 24.9, 24.2, 22.5.

IR (neat): 3049, 2827, 2923, 2712, 1814, 1734, 1656, 1451, 1321, 1258, 1155, 1030, 970, 779, 700 cm^{-1}.

HRMS for $C_{19}H_{23}NO_4$ [M+H]+: m/z calcd: 330.1700, found: 330.1707.

HPLC (Chiralpak AD-H, eluent: *n*-hexane:iPrOH = 100/3, 0.5 mL· min^{-1}, 20 °C, λ = 254 nm) retention times: t_{major} = 12.38 min, t_{minor} = 14.14 min.

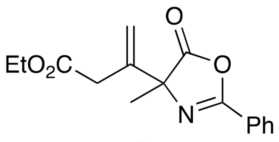

5n

Compound 5n: Obtained as a colorless oil in 71% yield with er = 75:25.

$[\alpha]_D^{22}$ (c = 0.98, $CHCl_3$) = +19.7°.

^1H NMR (300 MHz, δ, $CDCl_3$, 298 K): 7.93 (2H, dd, J = 8.6, 1.4 Hz), 7.51 (1H, t, J = 7.5 Hz), 7.42 (2H, t, J = 7.4 Hz), 5.52 (1H, s), 5.21 (1H, s), 3.86-4.04 (2H, m), 3.09 (1H, d, J = 16.3 Hz), 3.29 (1H, d, J = 16.3 Hz), 1.61 (3H, s), 1.07 (3H, t, J = 7.14 Hz).

$^{13}C\{1H\}$ NMR (75 MHz, δ, $CDCl_3$, 298 K): 178.55, 170.90, 160.62, 139.65, 132.86, 129.20, 128.78, 128.50, 127.99, 119.06, 117.67, 71.17, 60.94, 38.84, 25.18, 13.95.

IR (neat): 3012, 2818, 2714, 1815, 1712, 1646, 1515, 1484, 1369, 1161, 1027, 915, 711 cm^{-1}.

HRMS for $C_{16}H_{17}NO_4$ [M+H]+: m/z calcd: 288.1230, found: 288.1238.

HPLC (Chiralpak AD-H, eluent: *n*-hexane:iPrOH = 100/3, 0.5 mL·min-1, 20 °C, λ = 254 nm) retention times: t_{major} = 16.47 min, t_{minor} = 19.96 min.

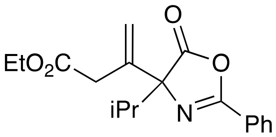

5o

Compound 5o: Obtained as a colorless oil in 81% yield with er = 74:26.

$[\alpha]_D^{22}$ (c = 0.97 $CHCl_3$) = +25.7.

^{1}H NMR (300 MHz, δ, $CDCl_3$, 298 K): 7.99-8.08 (2H, m), 7.55-7.63 (1H, m), 7.45-7.50 (2H, m), 5.64 (1H, s), 5.31 (1H, s), 3.87-4.03 (2H, m), 3.38 (1H, dd, *J* = 16.3, 1.0 Hz), 3.22 (1H, dd, *J* = 16.3, 0.6 Hz), 2.50-2.45 (1H, m), 1.14 (3H, t, *J* = 7.1 Hz), 1.07 (3H, d, *J* = 6.7 Hz), 0.89 (3H, d, *J* = 6.7 Hz).

^{13}C{1H} NMR (75 MHz, δ, $CDCl_3$, 298 K): 178.3, 171.0, 160.5, 138.9, 132.7, 128.7, 128.0, 125.8, 117.7, 78.3, 60.8, 39.3, 36.5, 16.7, 16.7, 13.9.

IR (neat):3042, 2945, 1840, 1734, 1657, 1322, 1156, 1059, 1026, 964, 882, 798, 692 cm^{-1}.

HRMS for $C_{18}H_{21}NO_4$ [M+H]+: m/z calcd: 316.1543, found: 316.1558.

HPLC (Chiralpak AD-H, eluent: *n*-hexane:iPrOH = 100/3, 0.5 mL· min^{-1}, 20 °C, λ = 254 nm) retention times: t_{major} = 12.21 min, t_{minor} = 16.53 min.

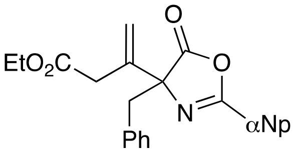

5p

Compound 5p: Obtained as a colorless oil 91% yield with er = 73:27

$[\alpha]_D^{22}$ (c = 1.00, $CHCl_3$) = –35.2°.

^{1}H NMR (300 MHz, δ, $CDCl_3$, 298 K): 8.19 (1H, s), 7.89 (1H, dd, *J* = 8.6, 1.6 Hz), 7.75-7.83 (3H, m), 7.40-7.51 (2H, m), 7.00-7.16 (5H, m), 5.73 (1H, s), 5.30 (1H, s), 3.81-4.00 (2H, m), 3.11-3.43 (4H, m), 1.05 (3H, t, *J* = 7.14 Hz).

^{13}C{1H} NMR (75 MHz, δ, $CDCl_3$, 298 K): 177.4, 171.0, 160.4, 139.2, 135.3, 133.8, 132.4, 130.4, 129.2, 129.1, 128.6, 128.3, 128.1, 127.9, 127.3, 126.9, 123.4, 122.8, 118.1, 76.1, 60.9, 45.0, 39.4, 13.9.

IR (neat): 3060, 2958, 1817, 1731, 1653, 1233, 1157, 1093, 1030, 986, 943, 901, 864, 822, 755, 700, 475 cm^{-1}.

HRMS for $C_{26}H_{23}NO_4$ [M+H]+: m/z calcd: 414.1700, found: 414.1712.

HPLC (Chiralpak AD-H, eluent: *n*-hexane:iPrOH = 100/3, 0.5 mL· min^{-1}, 20 °C, λ = 254 nm) retention times: t_{major} = 30.22 min, t_{minor}= 33.44 min.

3. Ring opening reactions

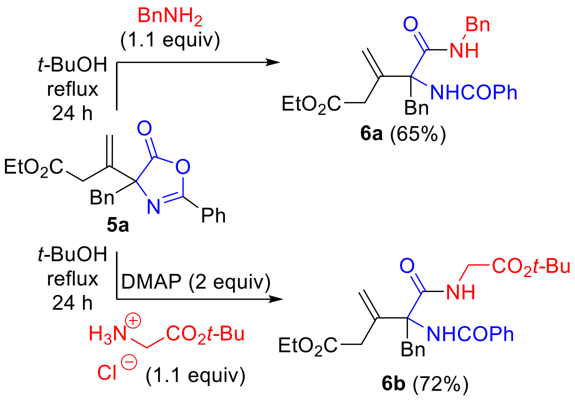

Compound 6a:

In a flame-dried Schlenk tube under Ar atmosphere at room temperature, 0.08 mmol of compound **5a** in 0.5 mL *tert*-butyl alcohol and 0.09 mmol of benzylamine (1.1 equivalents), were added. This mixture was refluxed for 24 hours in a preheated oil bath. After cooling to room temperature and evaporation of solvent under vacuum, the crude reaction mixture was purified through PTLC silica gel chromatography, using 33% ethyl acetate in heptane as eluent, yielding 65% of the pure product (0.024 g).

^{1}H NMR (300 MHz, δ, $CDCl_3$, 298 K): 7.96 (1H, s), 7.66-7.78 (3H, m), 7.46-7.54 (1H, m), 7.30-7.45 (7H, m), 7.06-7.22 (3H, m), 6.87-6.94 (2H, m), 5.54 (1H, s), 5.43 (1H, s), 4.42-4.60 (2H, m), 4.16 (2H, q, J = 7.1 Hz), 4.03 (1H, d, J = 12.9 Hz), 3.71 (1H, d, J = 17.3 Hz), 3.27 (1H, d, J = 17.4 Hz), 3.13 (1H, d, J = 12.9 Hz), 1.27 (3H, t, J = 7.14 Hz).

^{13}C{1H} NMR (75 MHz, δ, $CDCl_3$, 298 K): 173.4, 171.3, 165.9, 143.5, 137.7, 135.3, 134.6, 131.5, 130.0, 128.7, 128.5, 128.1, 128.0, 127.6, 127.0, 126.9, 119.6, 65.9, 61.3, 44.3, 39.1, 38.4, 14.1.

IR (neat): 3347, 3070, 2662, 2980, 1733, 1670, 1643, 1504, 1474, 1243, 1028, 695, 600 cm^{-1}.

HRMS for $C_{29}H_{30}N_2O_4$ [M+H]+: m/z calcd. 471.2278, found: 471.2283.

Compound 6b:

In a flame-dried Schlenk tube under argon atmosphere 0.08 mmol of compound **5a** in 0.5 mL *tert*-butyl alcohol were combined with 0.16 mmol DMAP (2 equiv) and 0.09 mmol of glycine *tert*-butyl ester hydrochloride (1.1 equivalents). This mixture was refluxed for 24 hours in a preheated oil bath. After cooling to room temperature and evaporation of solvent under vacuum, the crude reaction mixture was purified through PTLC silica gel chromatography using 33% ethyl acetate in heptane as eluent, yielding 72% of the pure product (0.028 g).

1H NMR (300 MHz, δ, $CDCl_3$, 298 K): 7.80 (1H, s), 7.65-7.74 (2H, m), 7.45-7.57 (2H, m), 7.36-7.45 (2H), 7.12-7.23 (3H, m), 7.03-7.11 (2H, m), 5.66 (1H, s), 5.52 (1H, s), 3.88-4.20 (5H, m), 3.60 (1H, d, J = 17.0 Hz), 3.20-3.31 (2H, m), 1.53 (9H, s), 1.25 (3H, t, J = 7.1 Hz).

$^{13}C\{1H\}$ NMR (75 MHz, δ, $CDCl_3$, 298 K): 172.9, 171.2, 168.3, 166.0, 143.0, 135.4, 134.6, 131.5, 130.1, 128.5, 128.0, 127.0, 119.6, 82.4, 66.2, 61.2, 42.5, 39.1, 38.6, 28.0, 14.0.

IR (neat): 3359, 1749, 1735, 1646, 1508, 1477, 1368, 1248, 1217, 1152, 1031, 745, 689, 597, 451 cm^{-1}.

HRMS for $C_{28}H_{34}N_2O_6$ [M+H]+: m/z calcd. 495.2489, found: 495.248.

4. NMR Spectra

NMR spectra of compound 5a

^{1}H NMR (300 MHz, $CDCl_3$, 298 K)

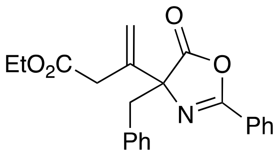

5a

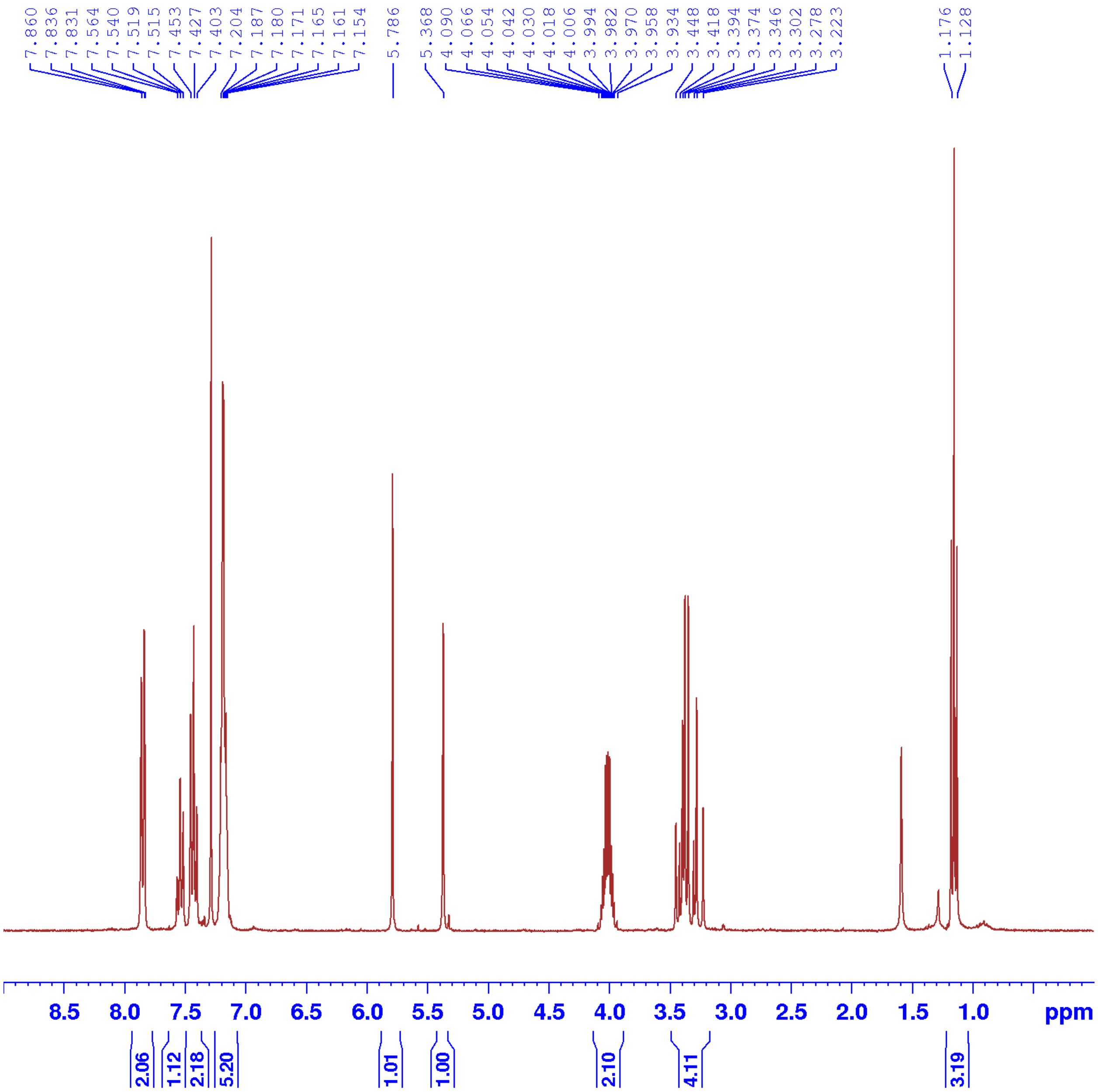

^{13}C NMR (75 MHz, $CDCl_3$, 298 K)

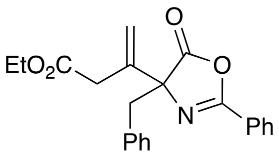

5a

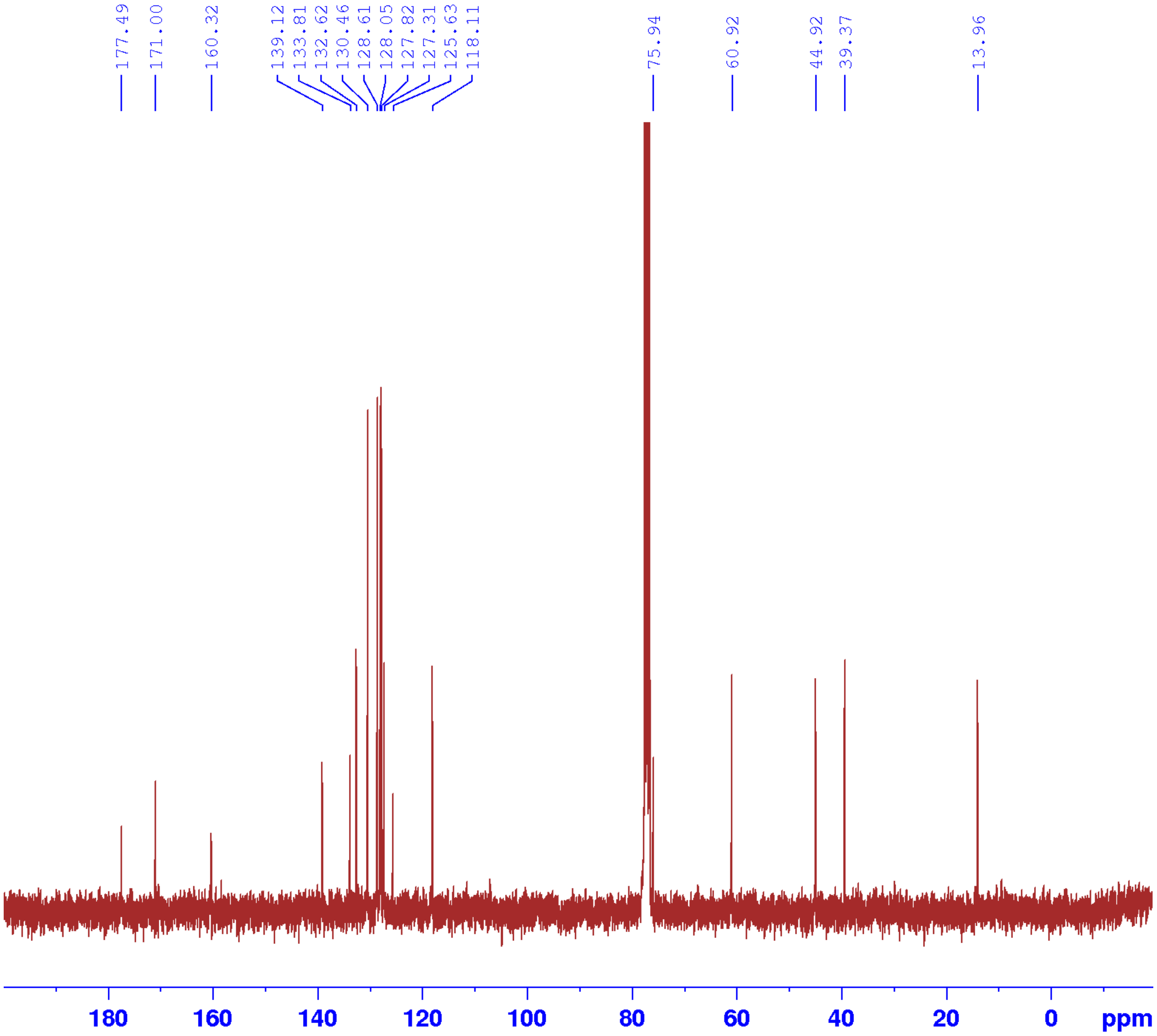

NMR spectra of compound 5b

^{1}H NMR (300 MHz, $CDCl_3$, 298 K)

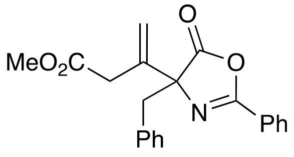

5b

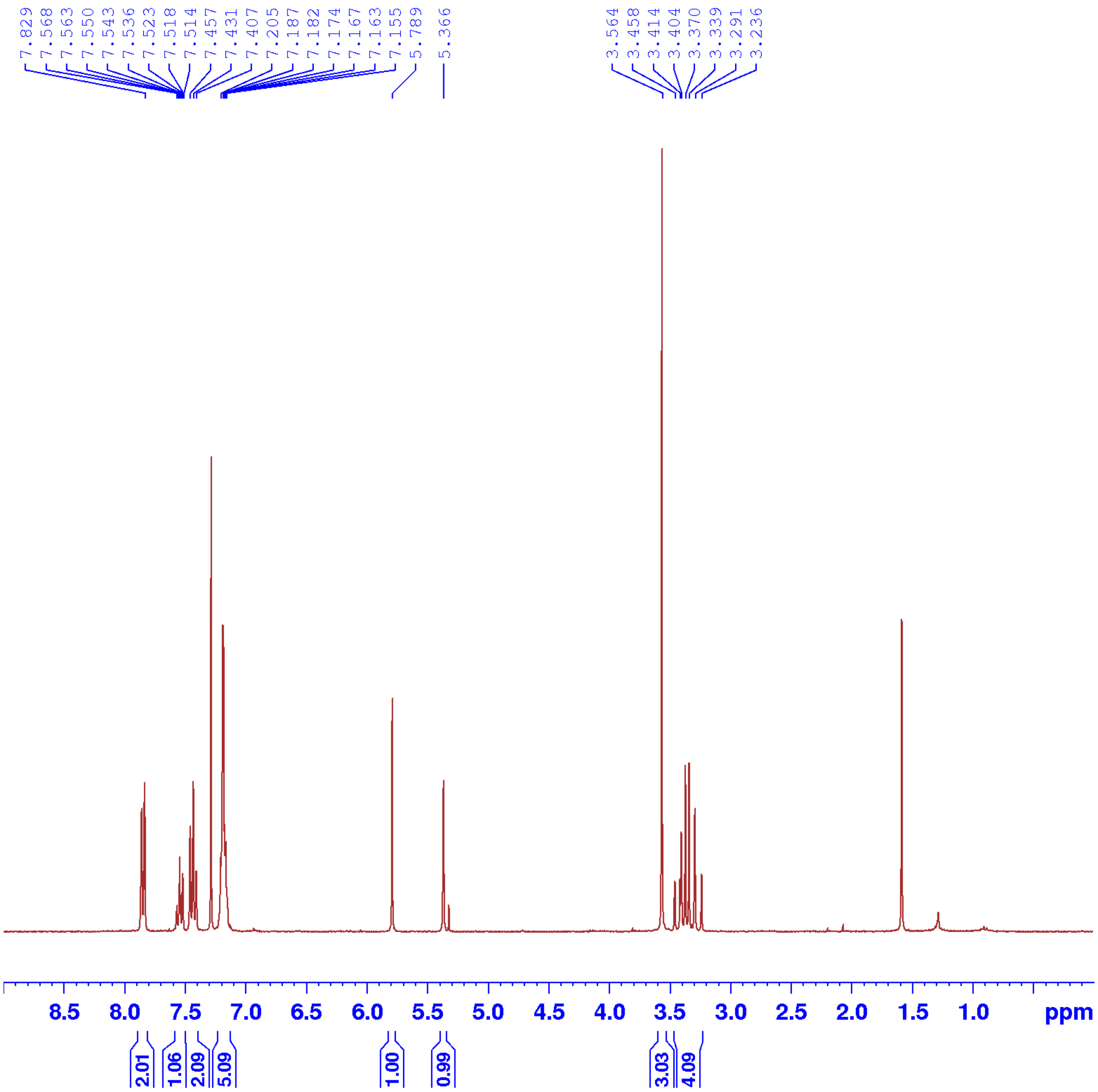

^{13}C NMR (75 MHz, $CDCl_3$, 298 K)

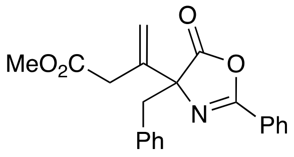

5b

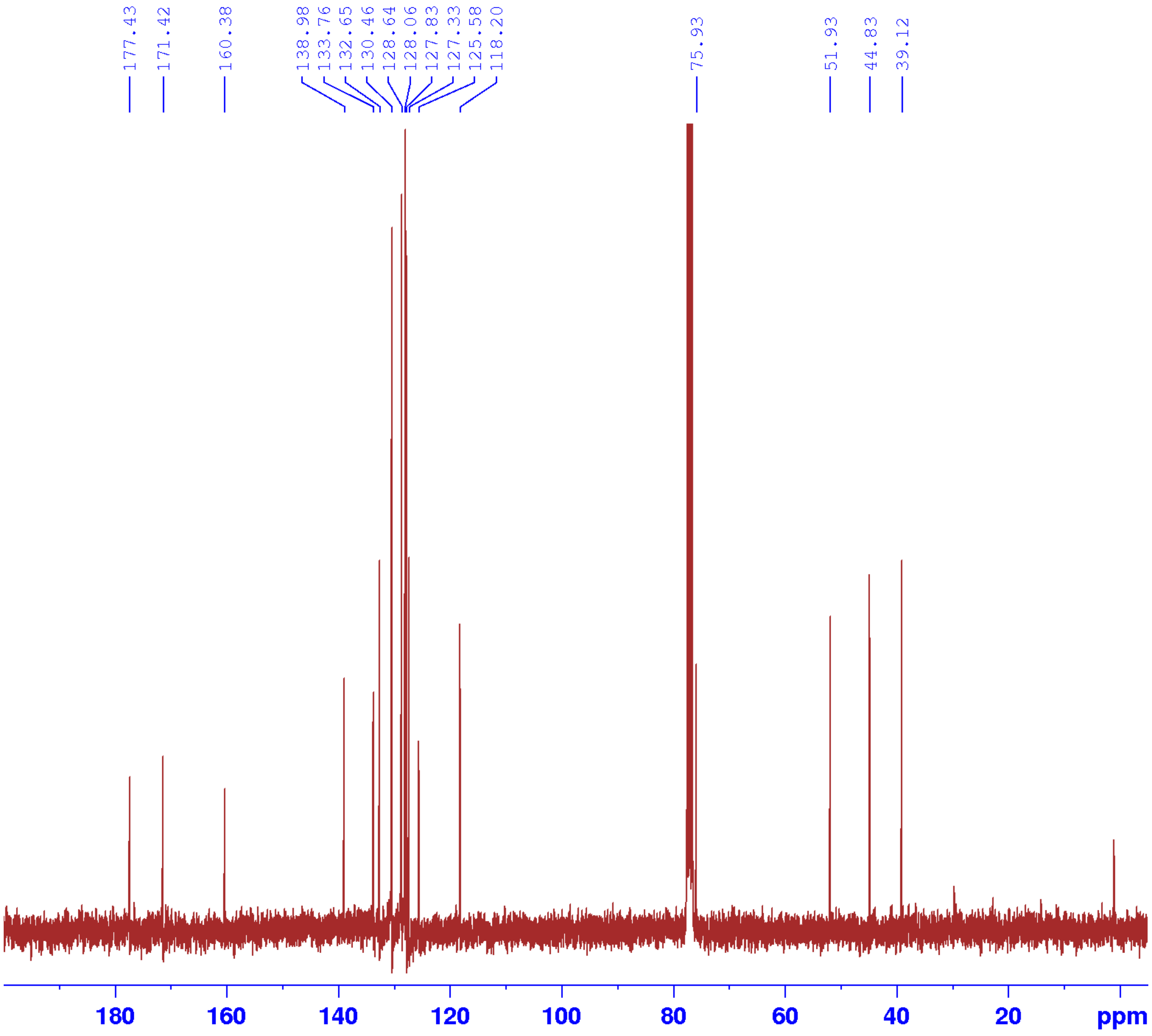

NMR spectra of compound 5c

^{1}H NMR (300 MHz, $CDCl_3$, 298 K)

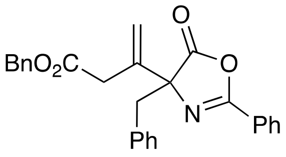

5c

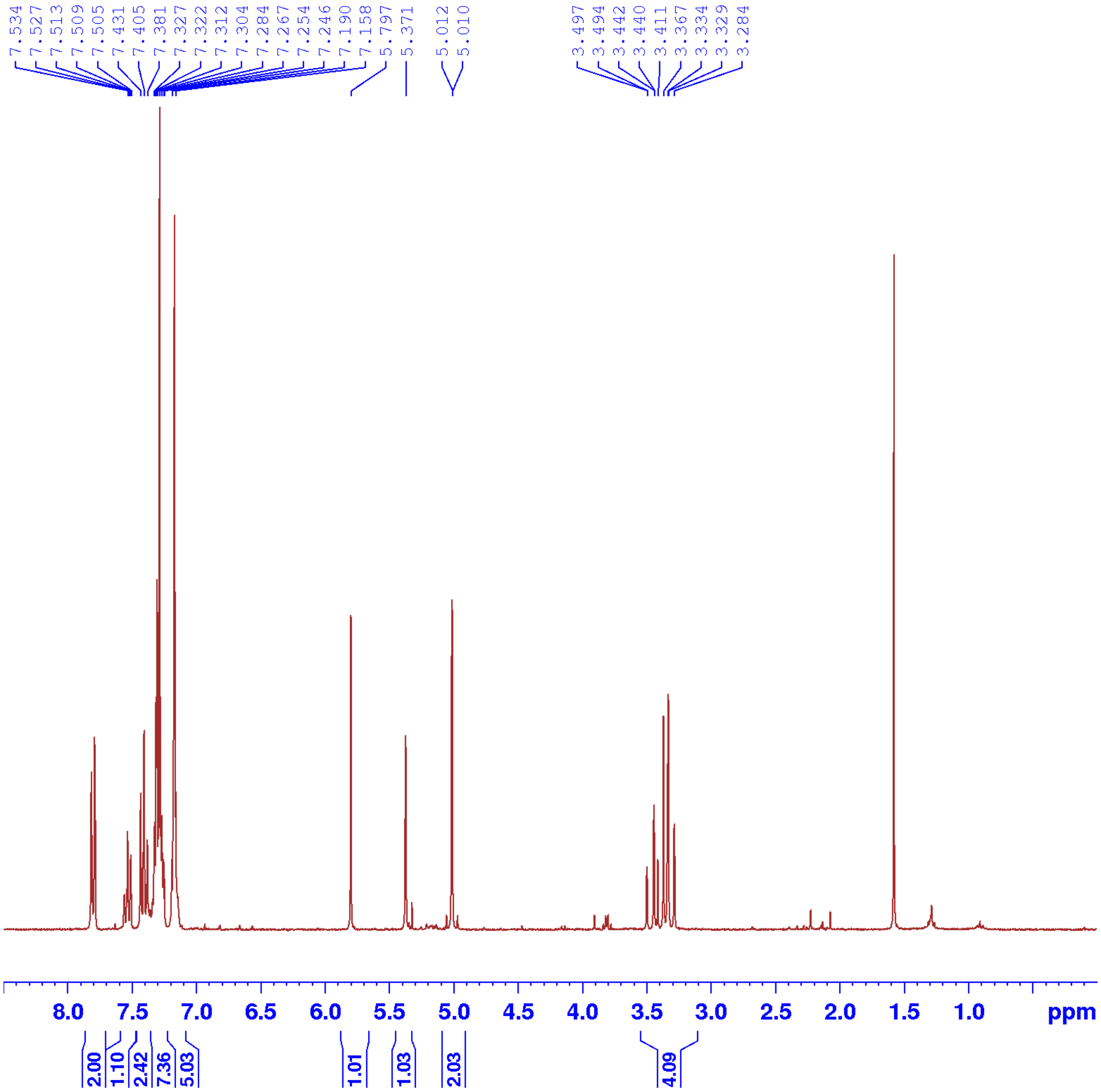

^{13}C NMR (75 MHz, $CDCl_3$, 298 K)

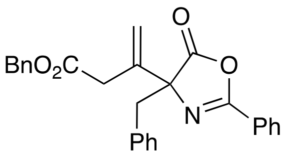

5c

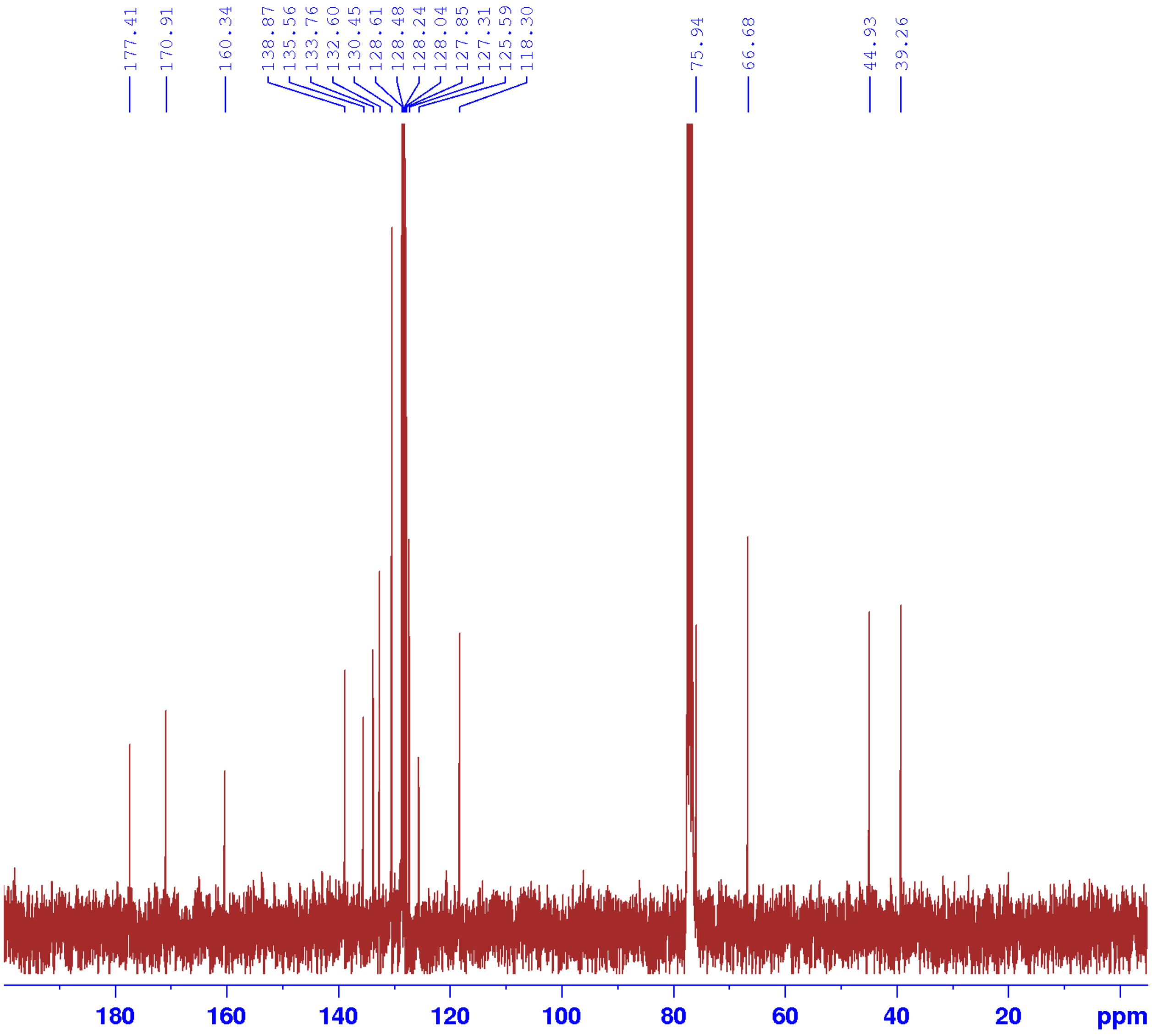

NMR spectra of compound 5d

^{1}H NMR (300 MHz, $CDCl_3$, 298 K)

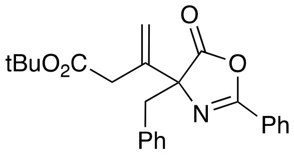

5d

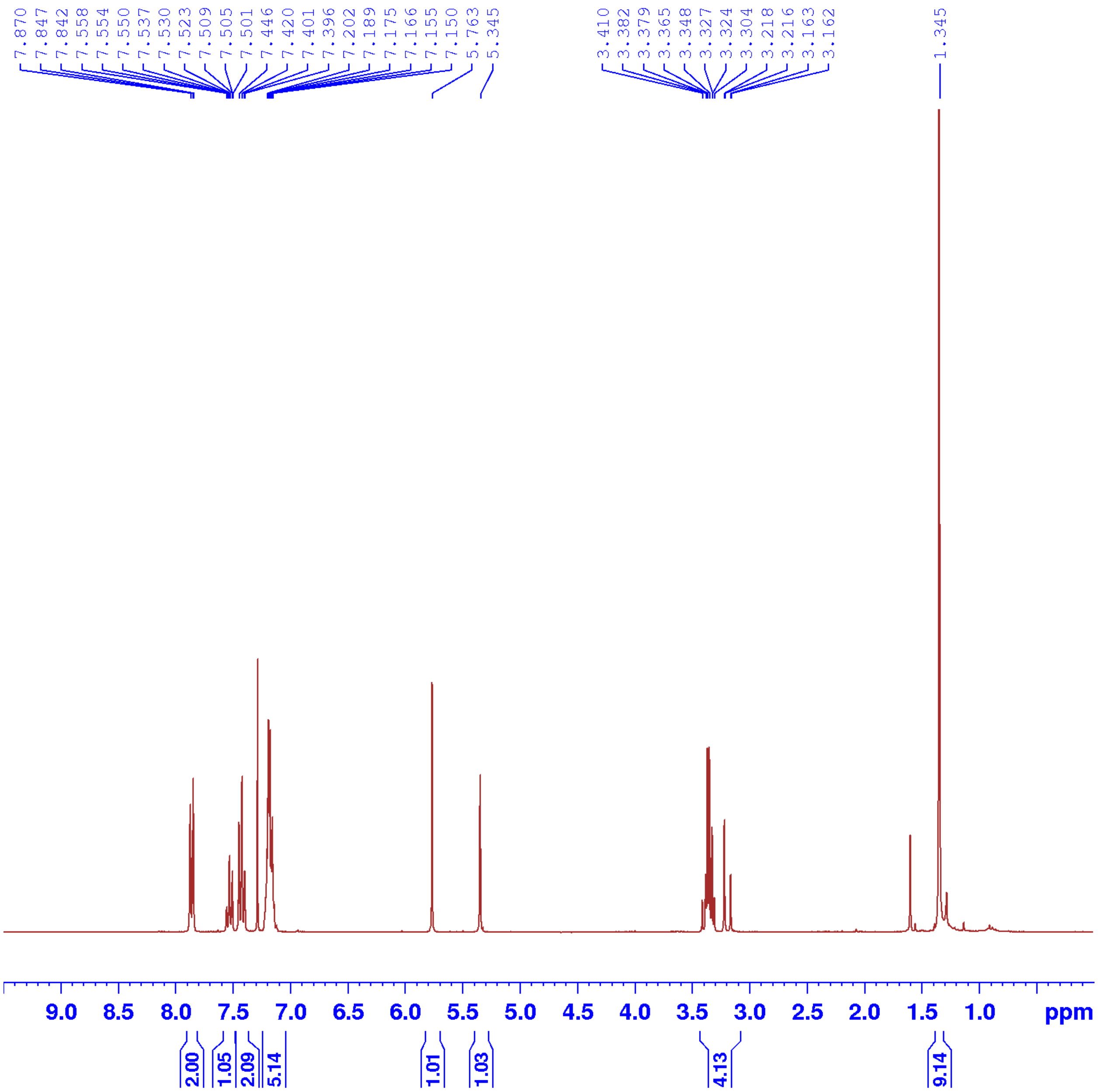

^{13}C NMR (75 MHz, $CDCl_3$, 298 K)

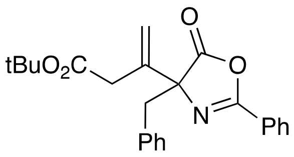

5d

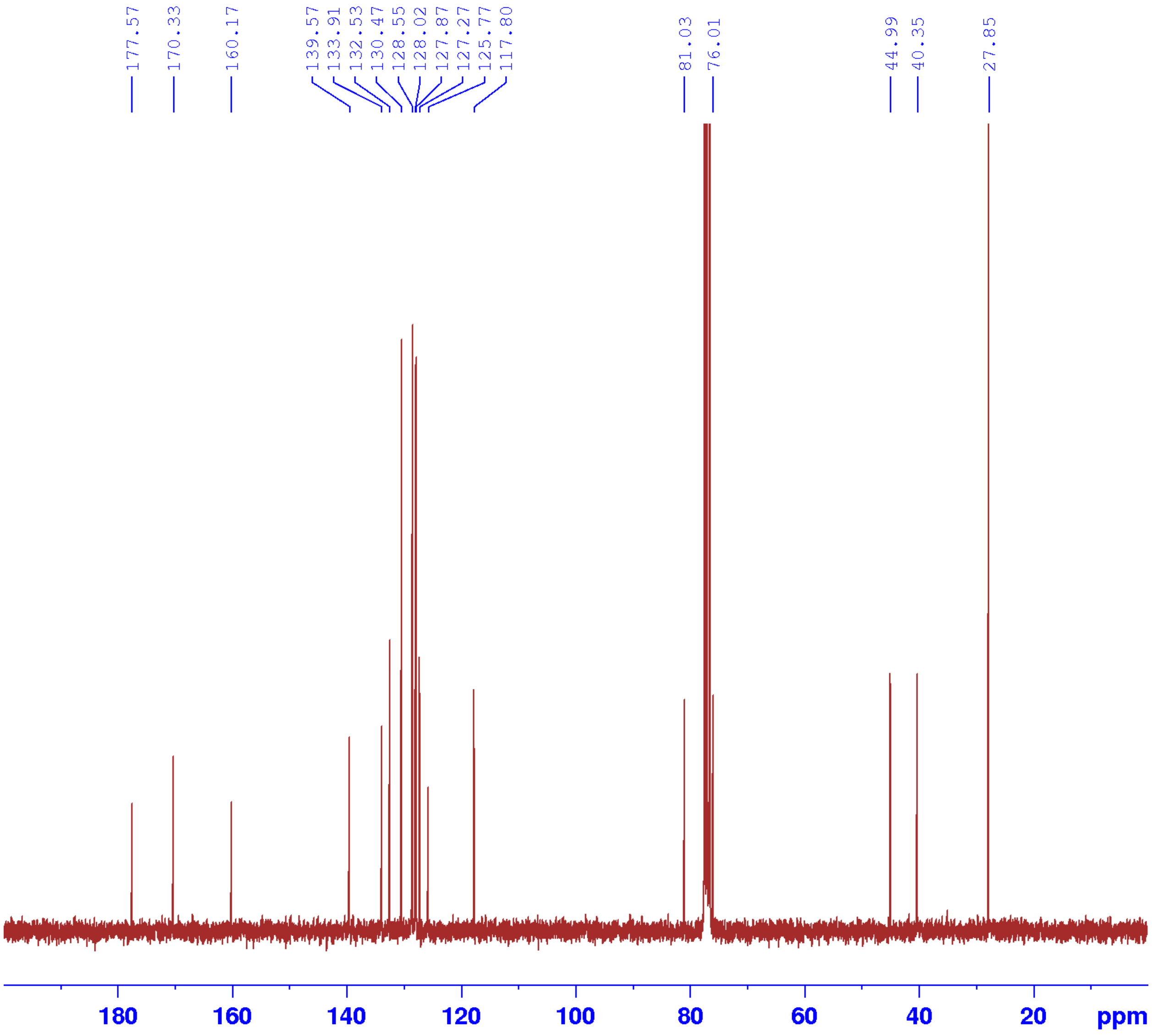

NMR spectra of compound 5e

^{1}H NMR (300 MHz, $CDCl_3$, 298 K)

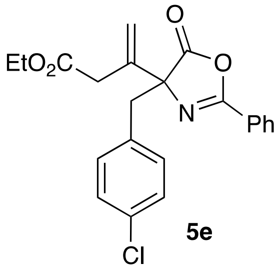

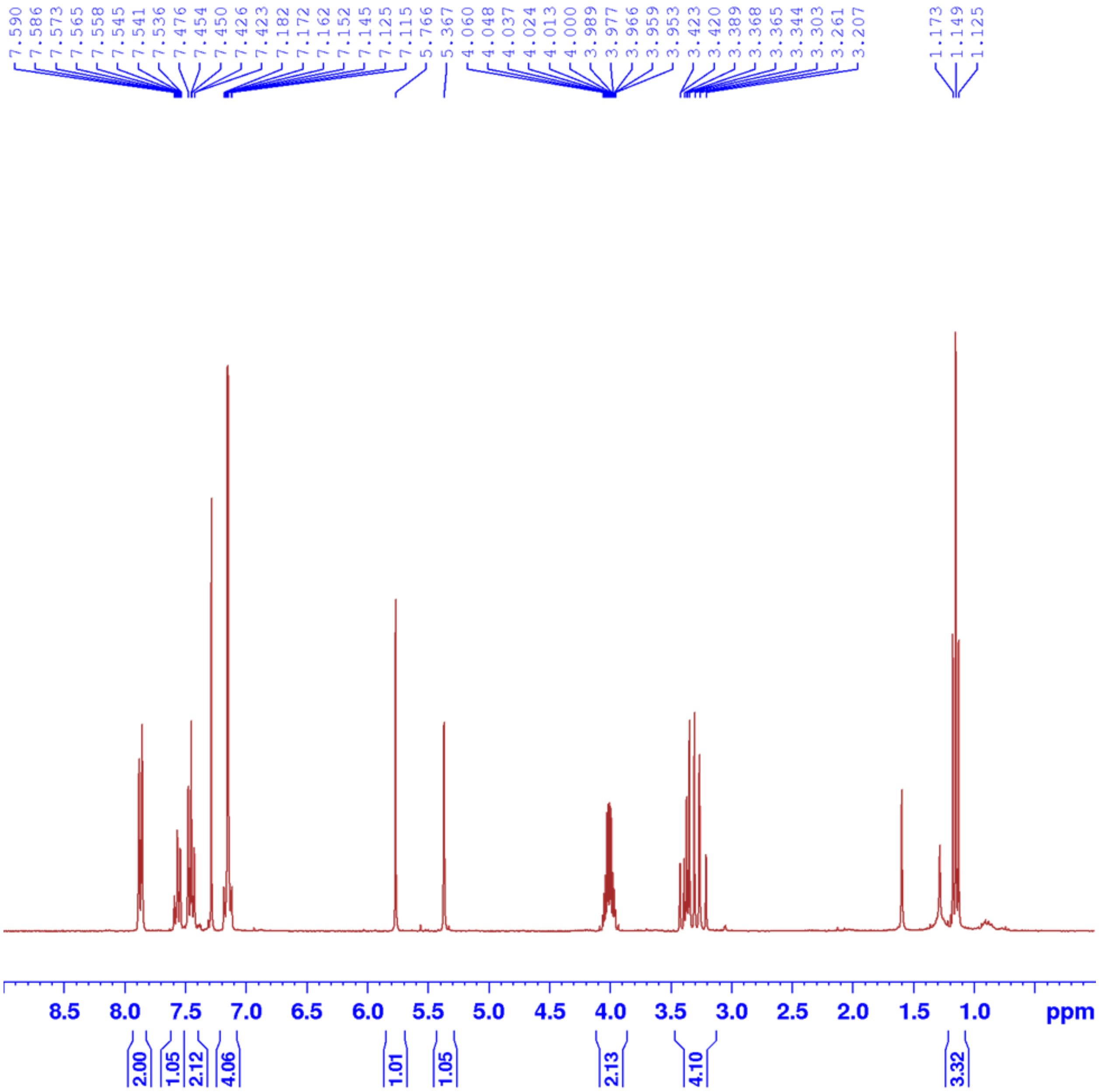

^{13}C NMR (75 MHz, $CDCl_3$, 298 K)

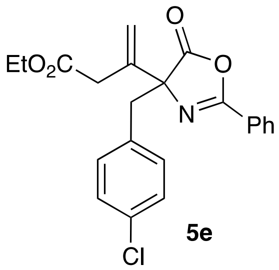

5e

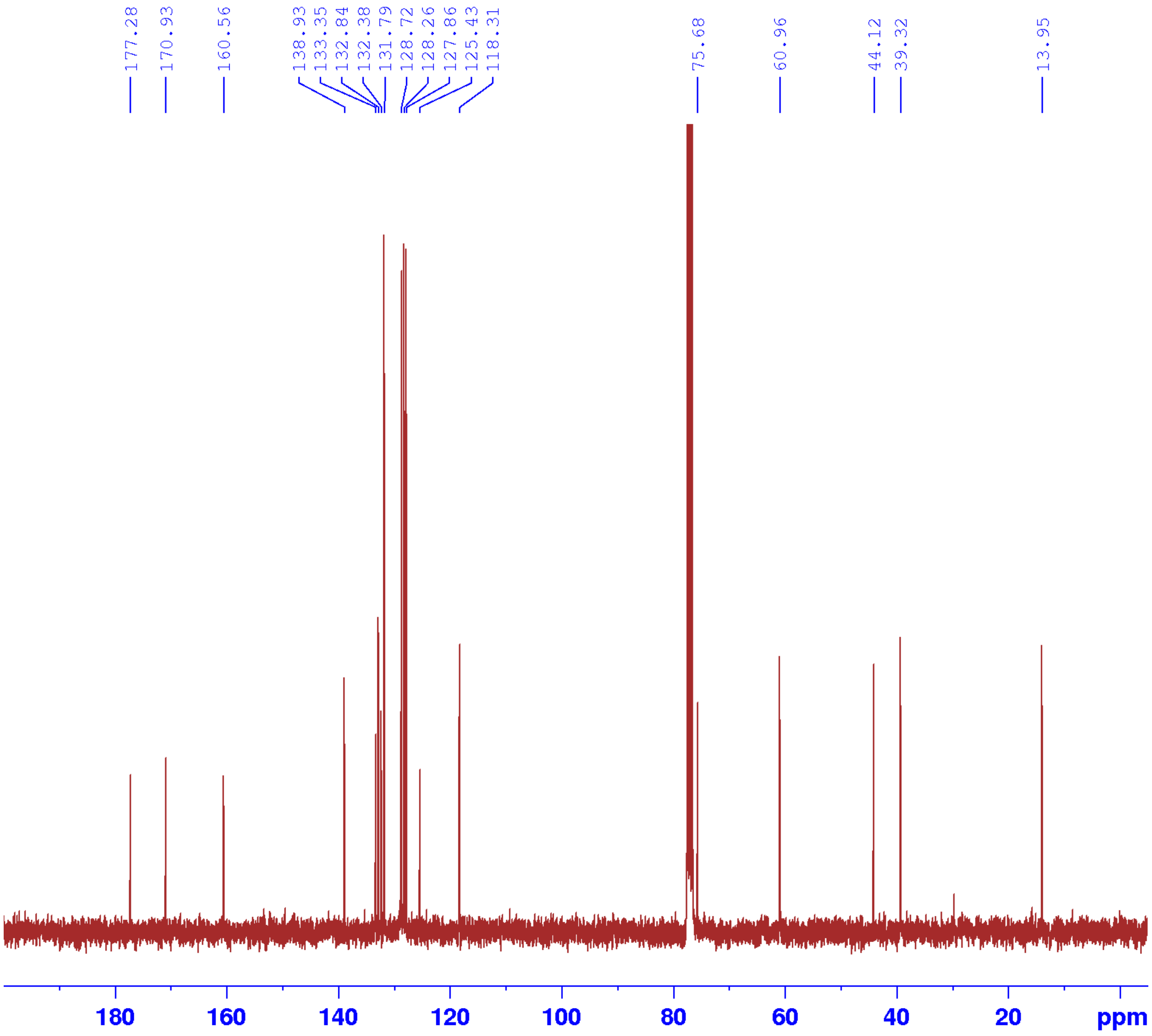

NMR spectra of compound 5f

^{1}H NMR (300 MHz, $CDCl_3$, 298 K)

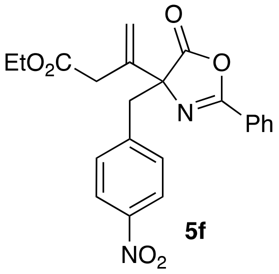

5f

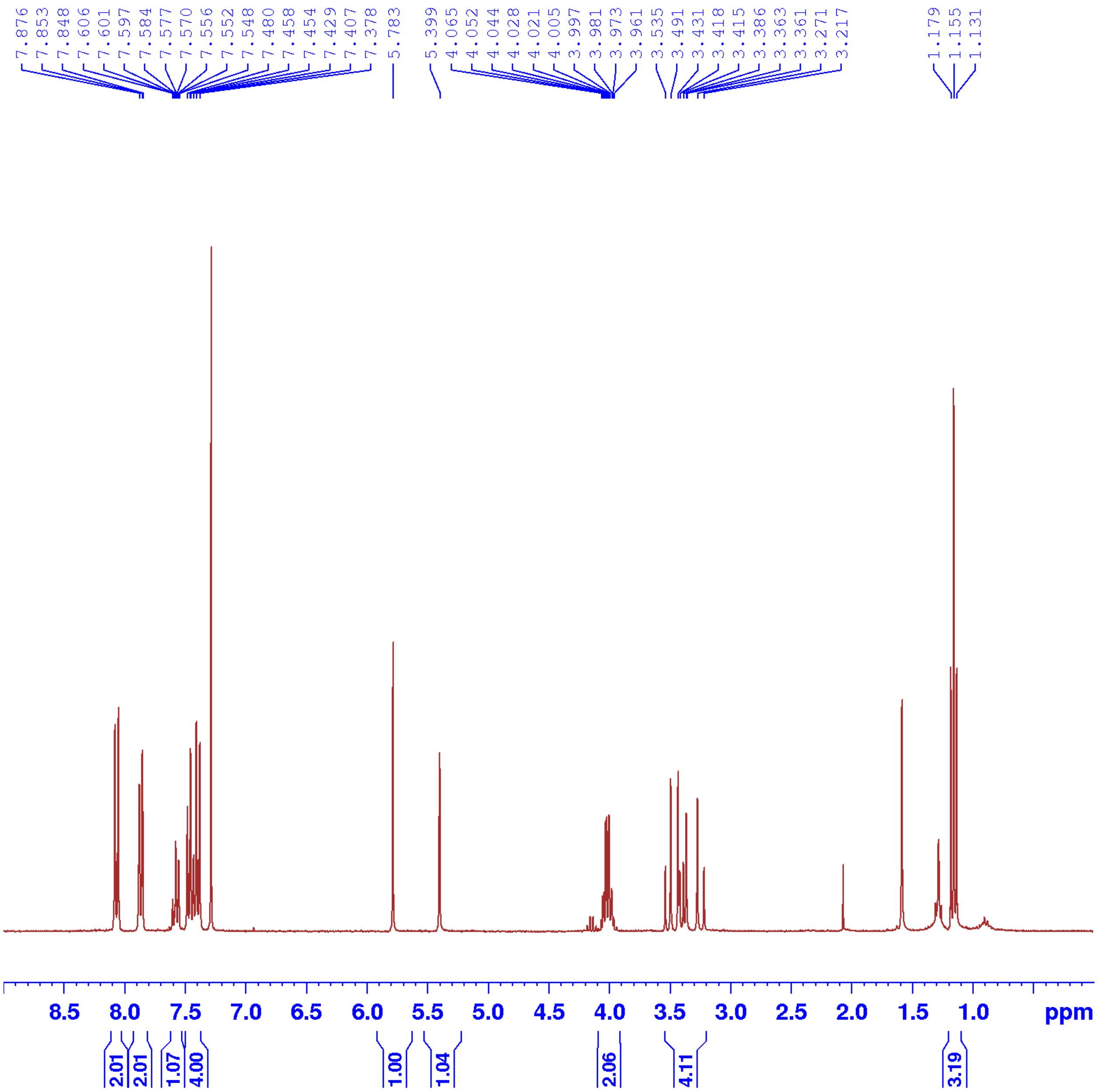

^{13}C NMR (75 MHz, $CDCl_3$, 298 K)

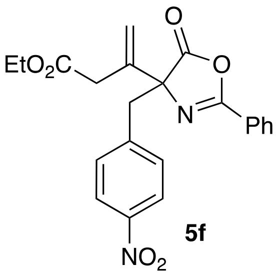

5f

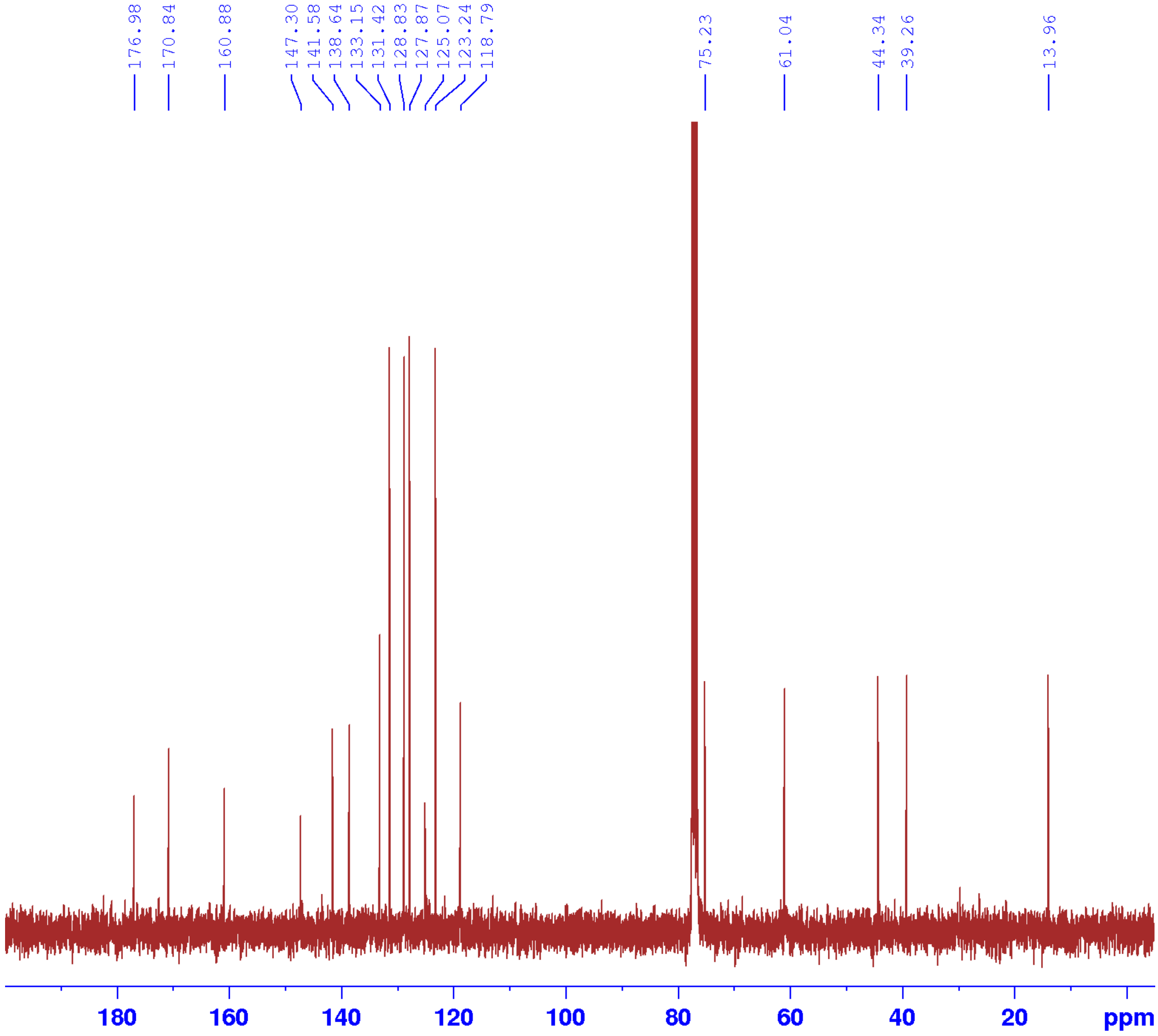

NMR spectra of compound 5g

^{1}H NMR (300 MHz, $CDCl_3$, 298 K)

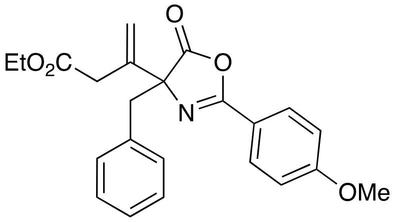

5g

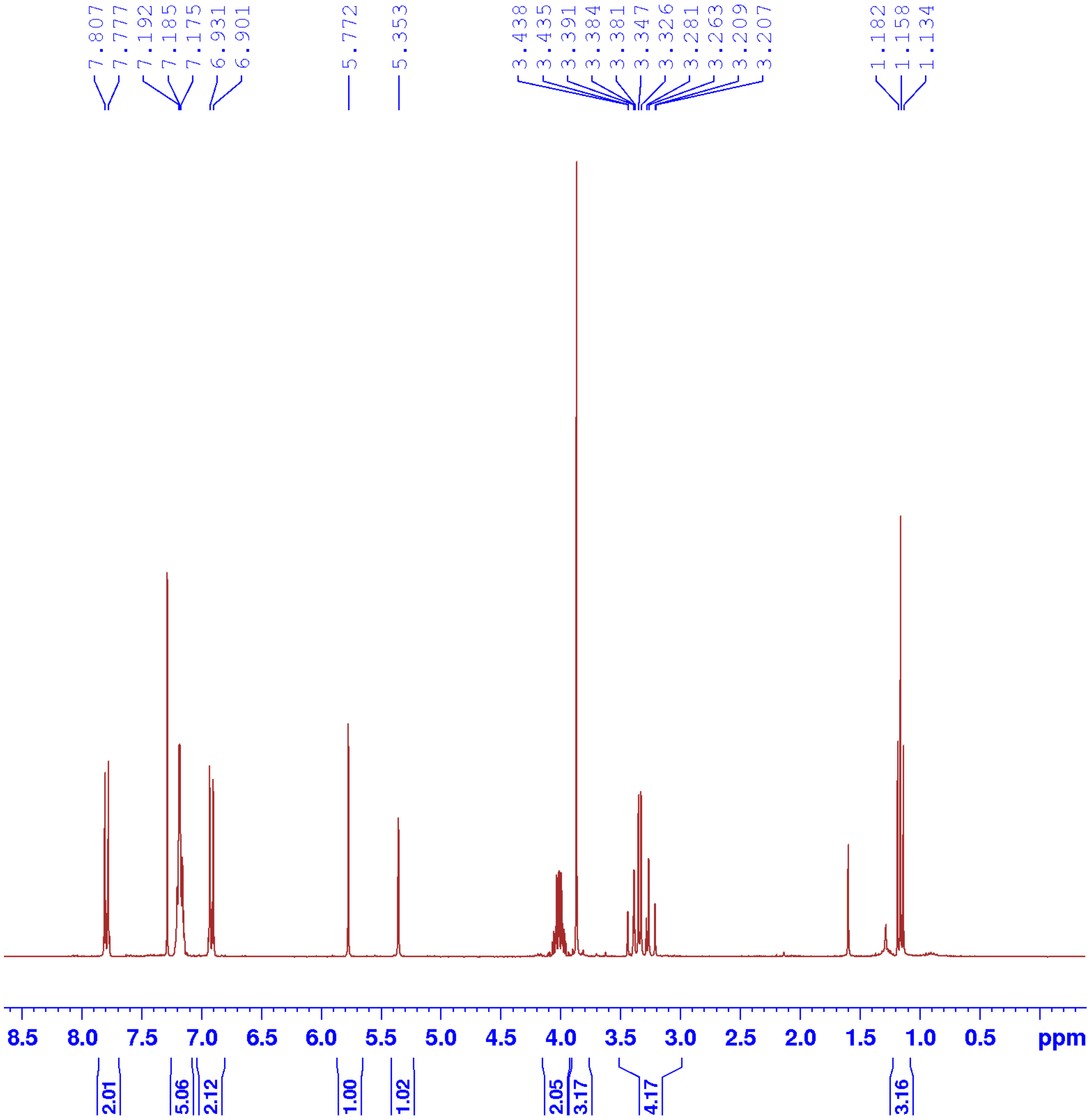

^{13}C NMR (75 MHz, $CDCl_3$, 298 K)

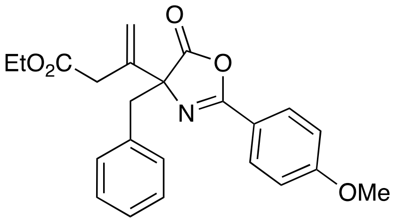

5g

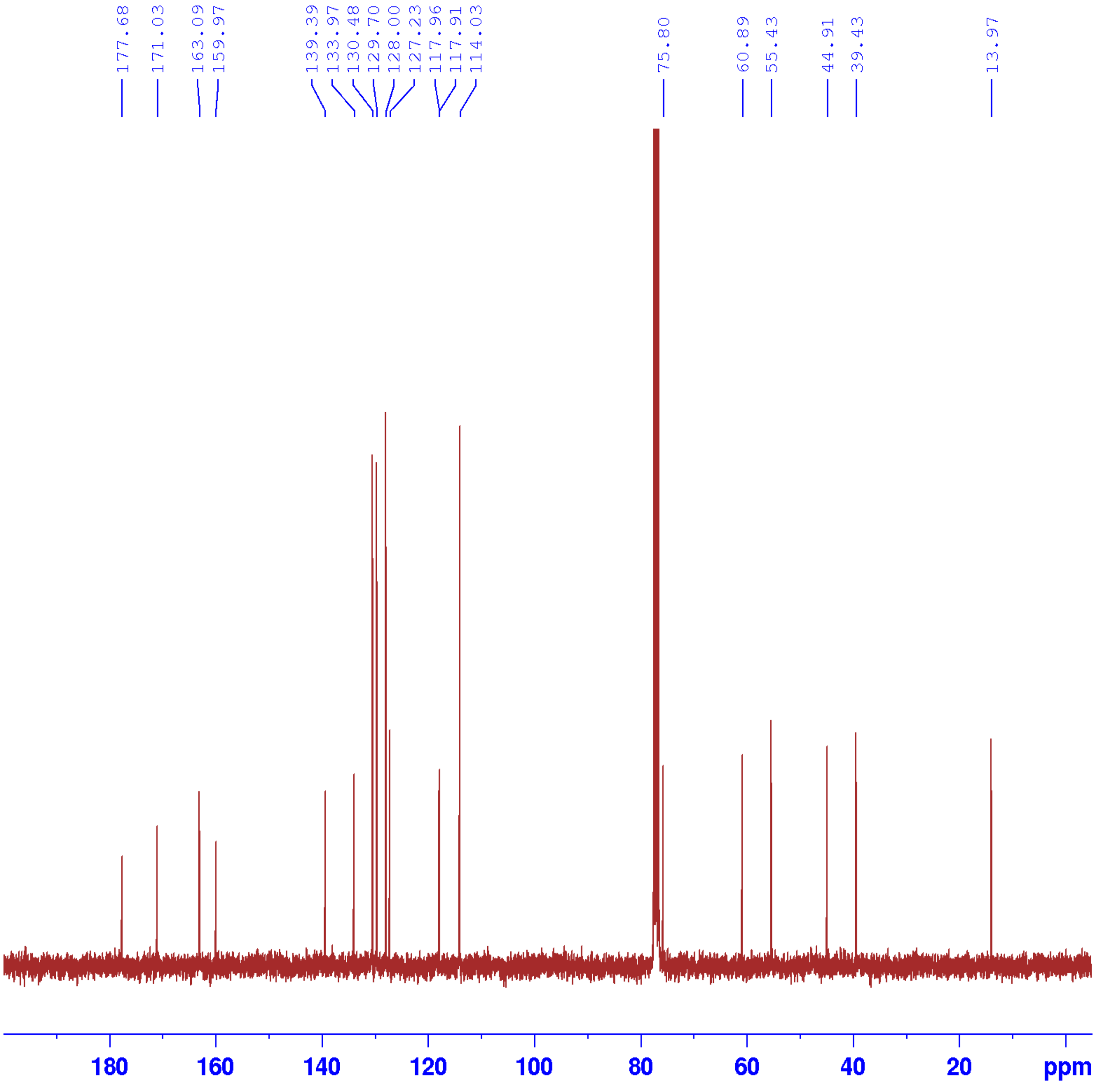

NMR spectra of compound 5h

^{1}H NMR (300 MHz, $CDCl_3$, 298 K)

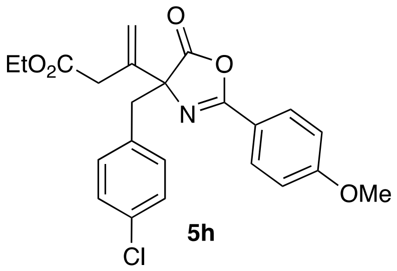

5h

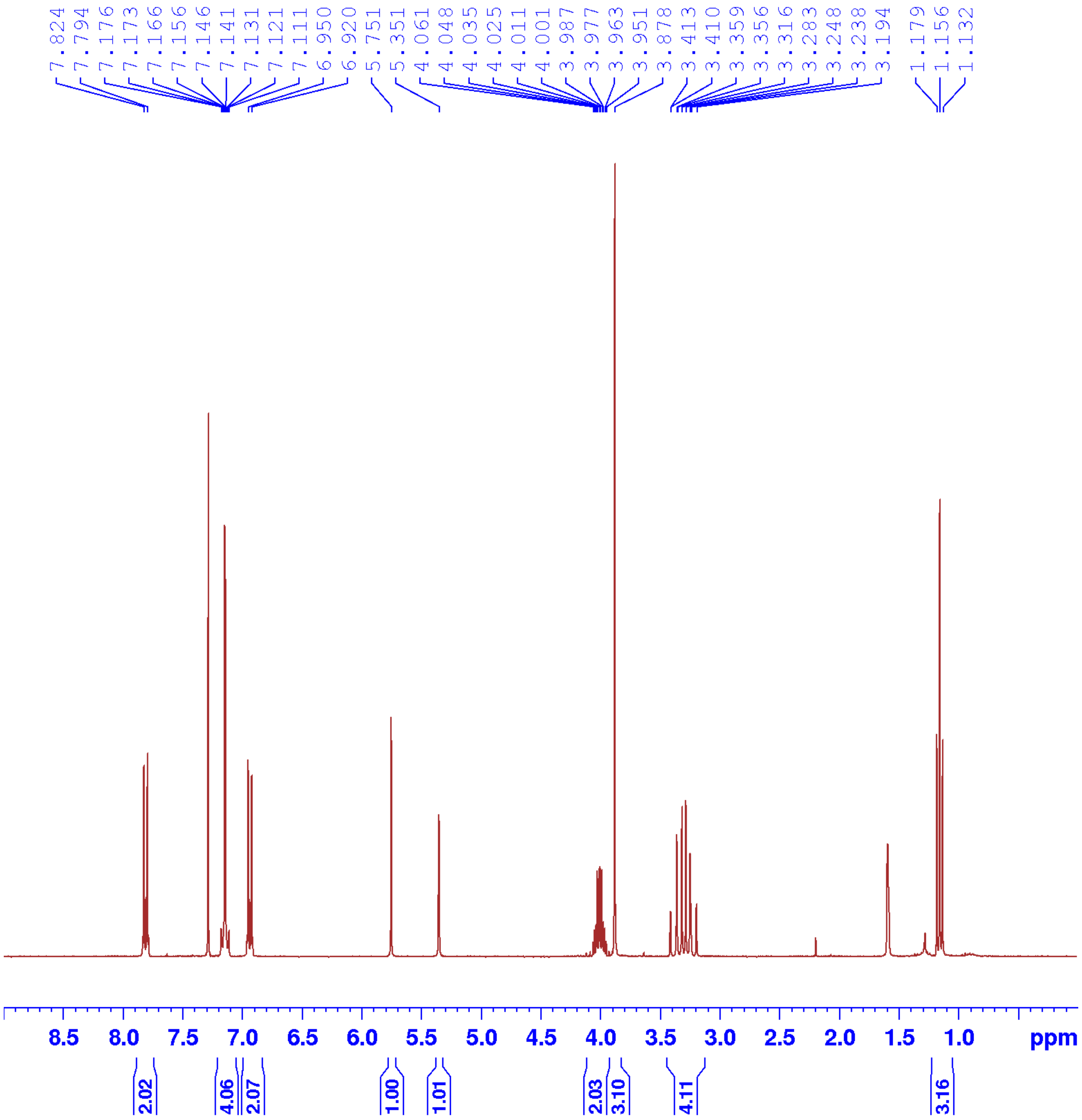

^{13}C NMR (75 MHz, $CDCl_3$, 298 K)

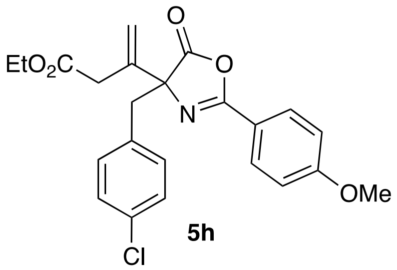

5h

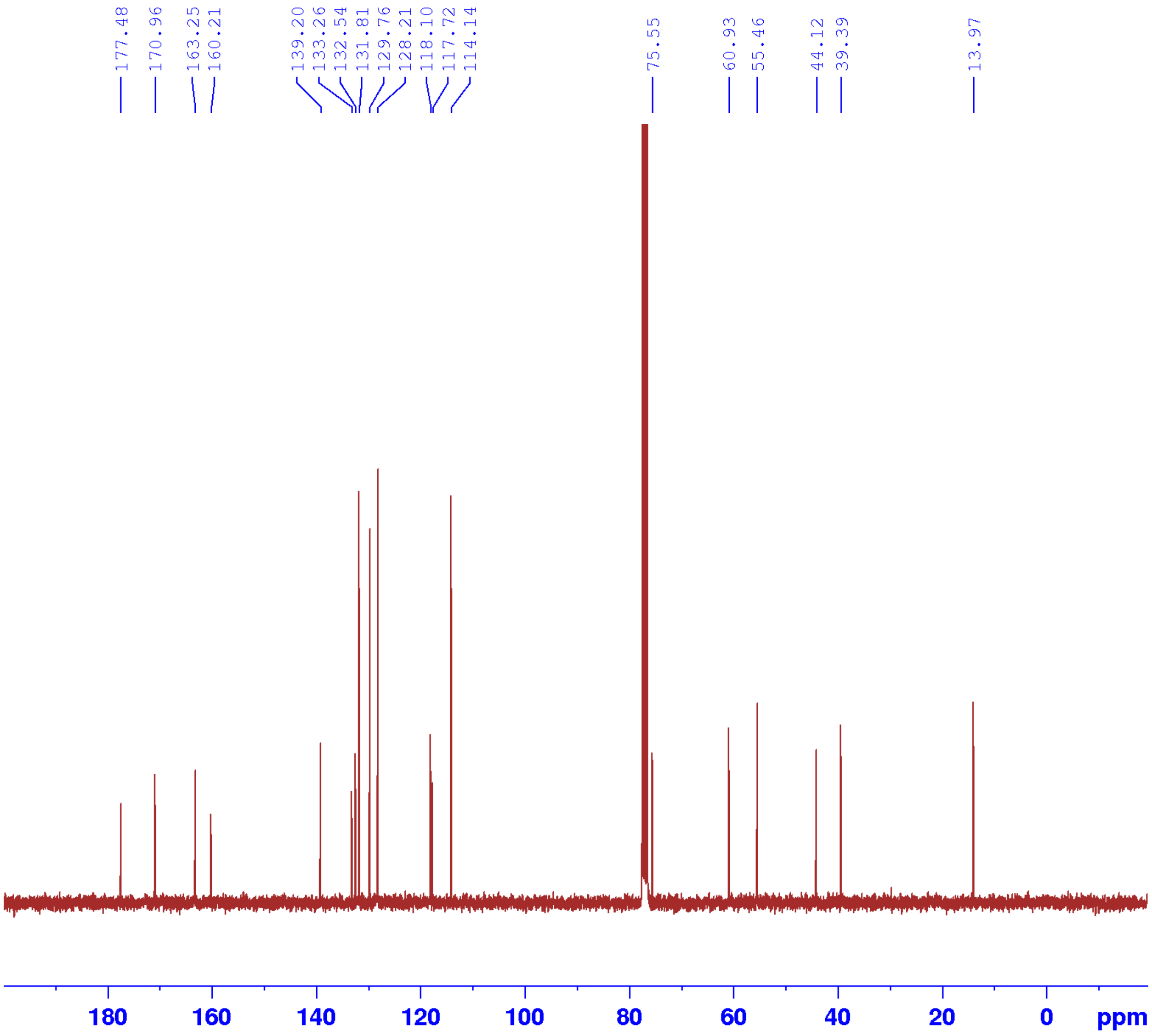

NMR spectra of compound 5i

^{1}H NMR (300 MHz, $CDCl_3$, 298 K)

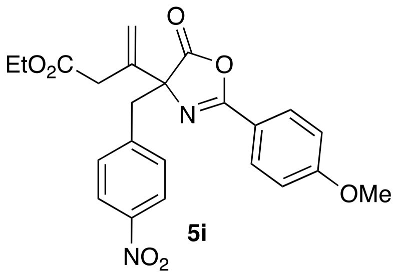

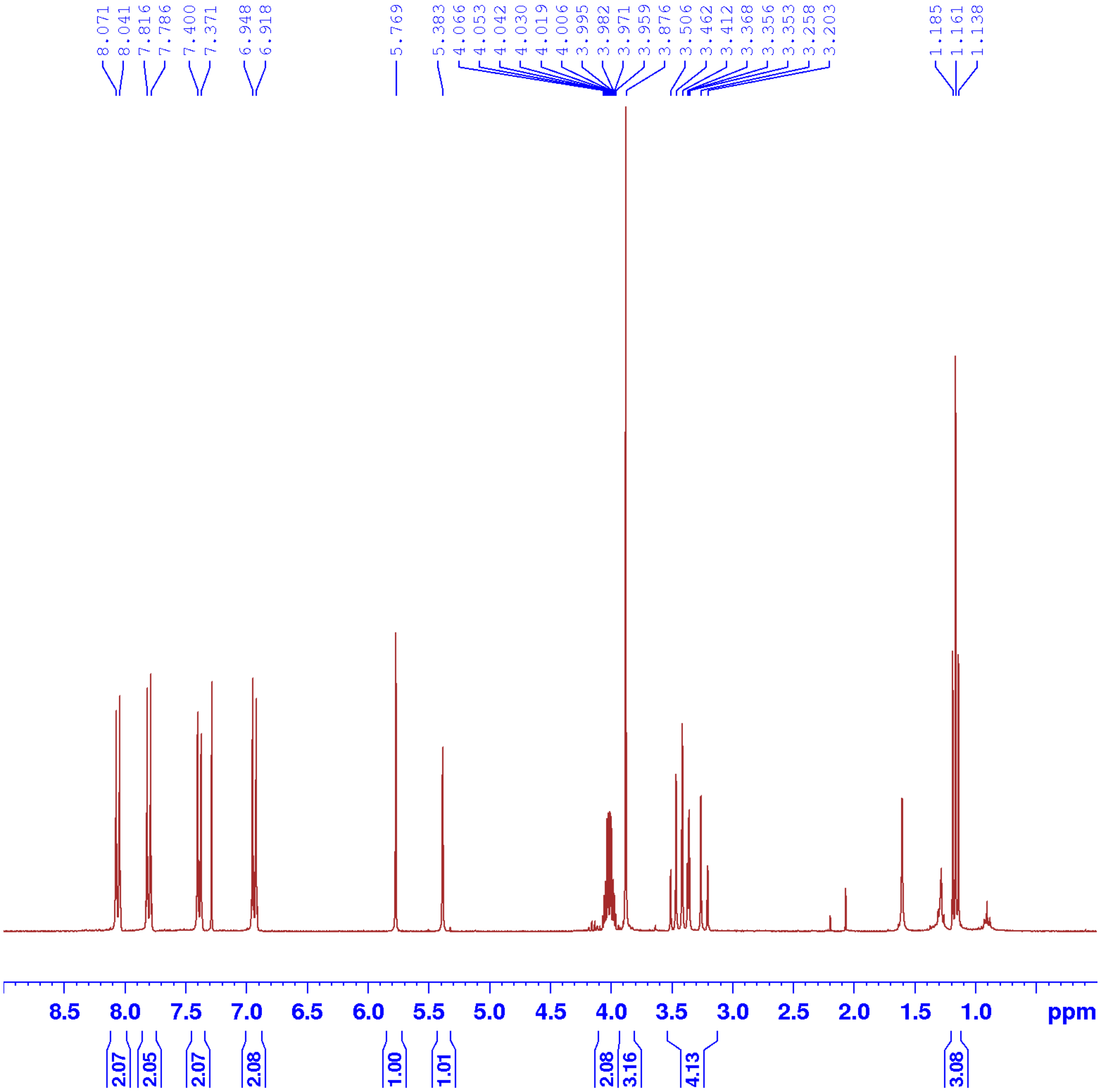

^{13}C NMR (75 MHz, $CDCl_3$, 298 K)

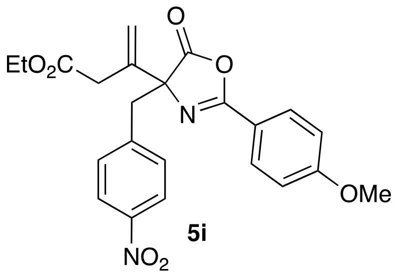

5i

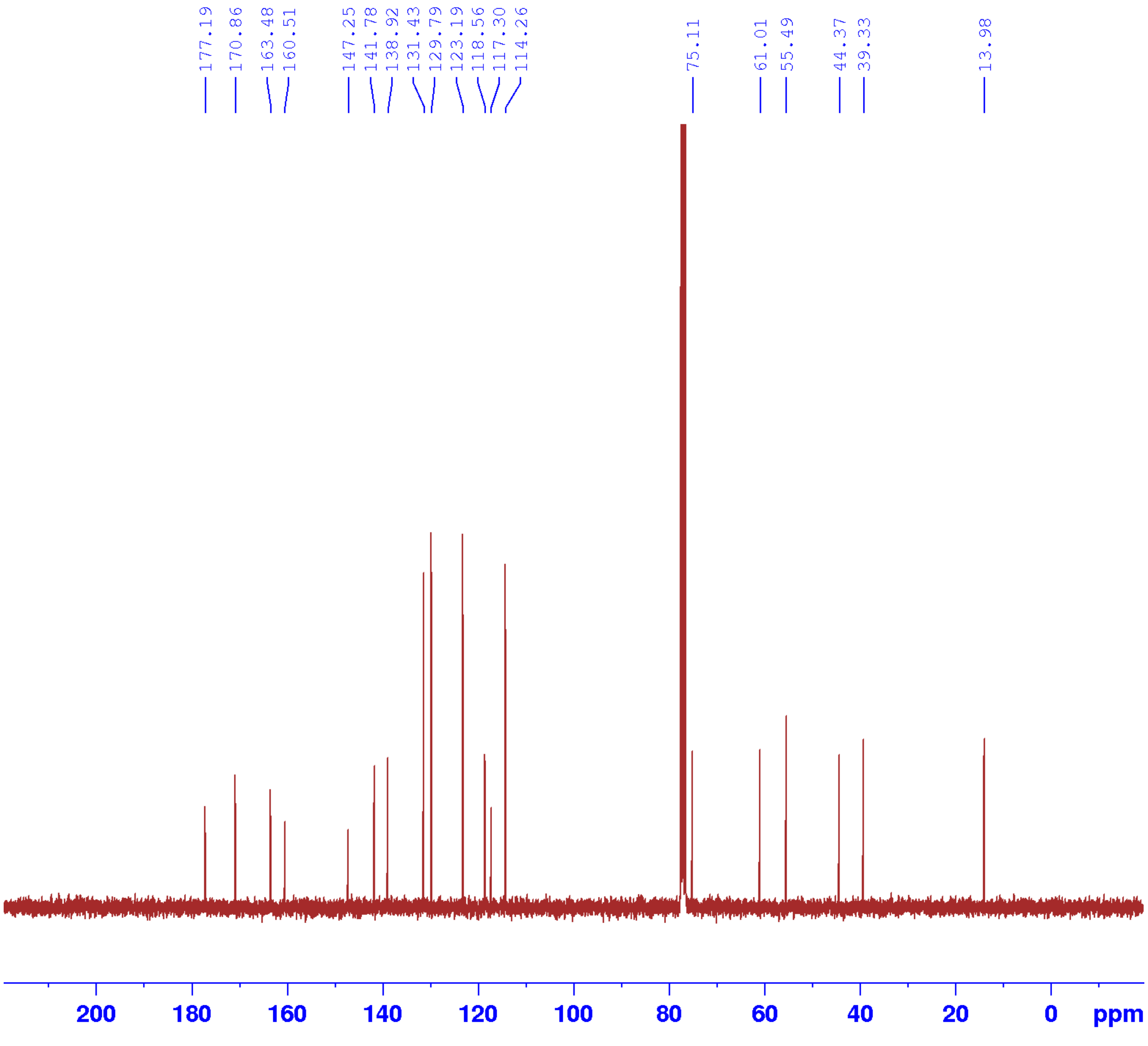

NMR spectra of compound 5j (*containing traces of ring-opened product*)

^{1}H NMR (300 MHz, $CDCl_3$, 298 K)

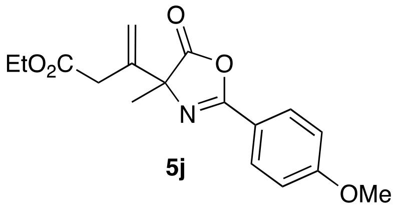

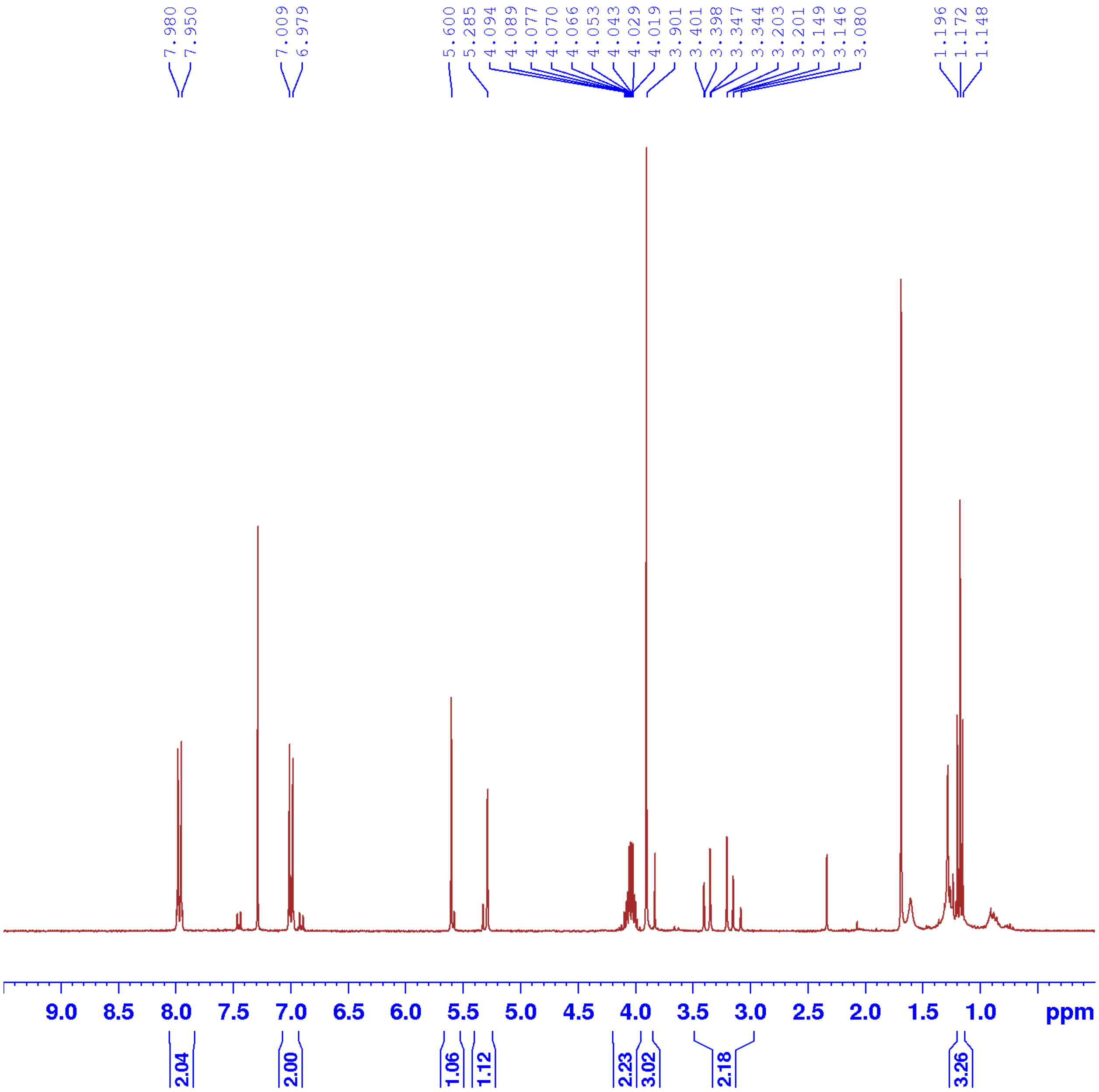

^{13}**C NMR** (75 MHz, $CDCl_3$, 298 K)

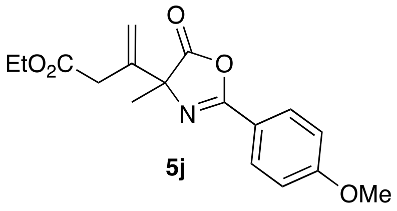

5j

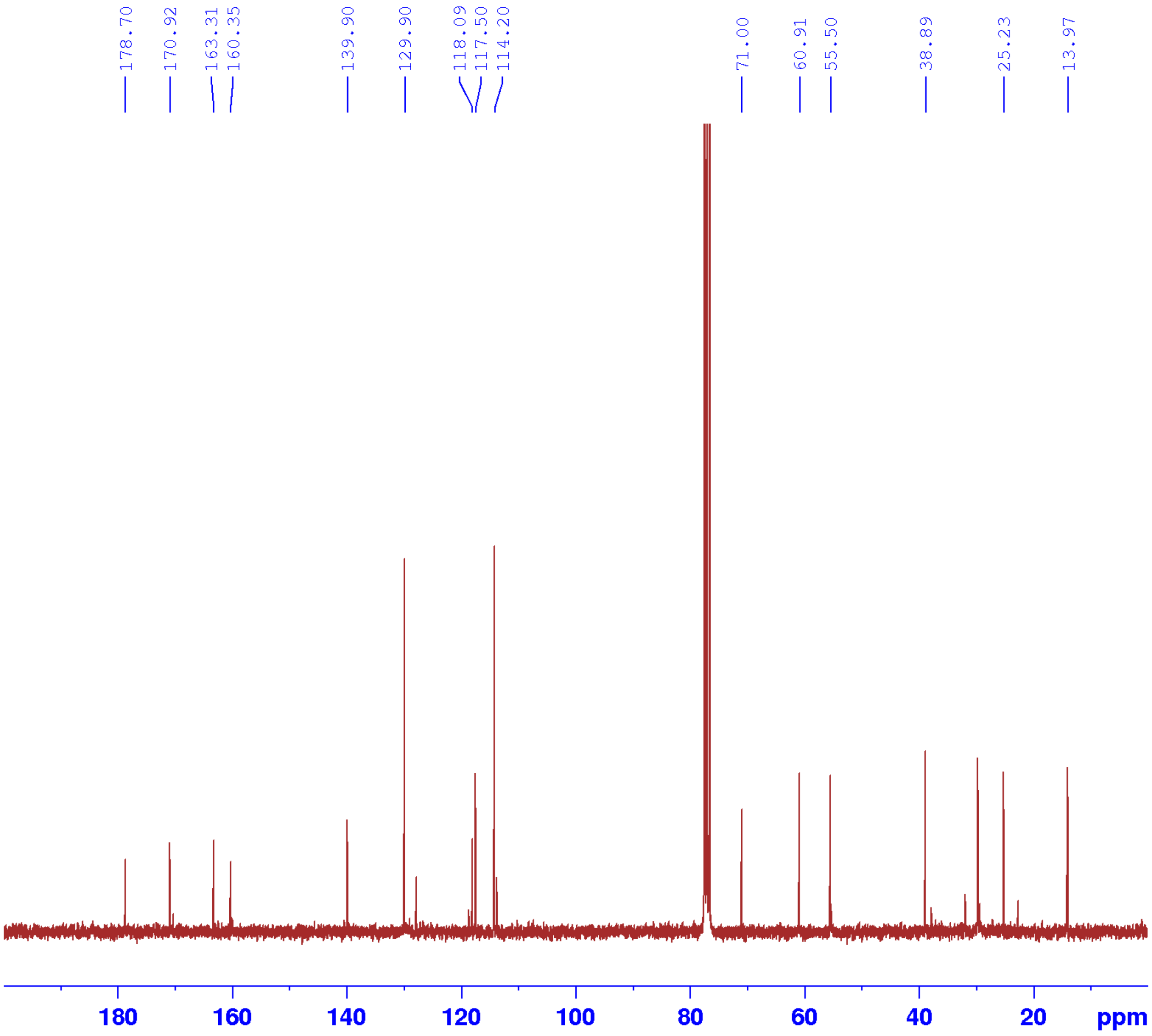

NMR spectra of compound 5k (*containing traces of ring-opened product*)

^{1}H NMR (300 MHz, $CDCl_3$, 298 K)

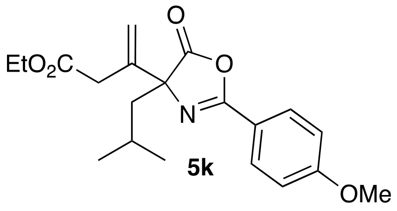

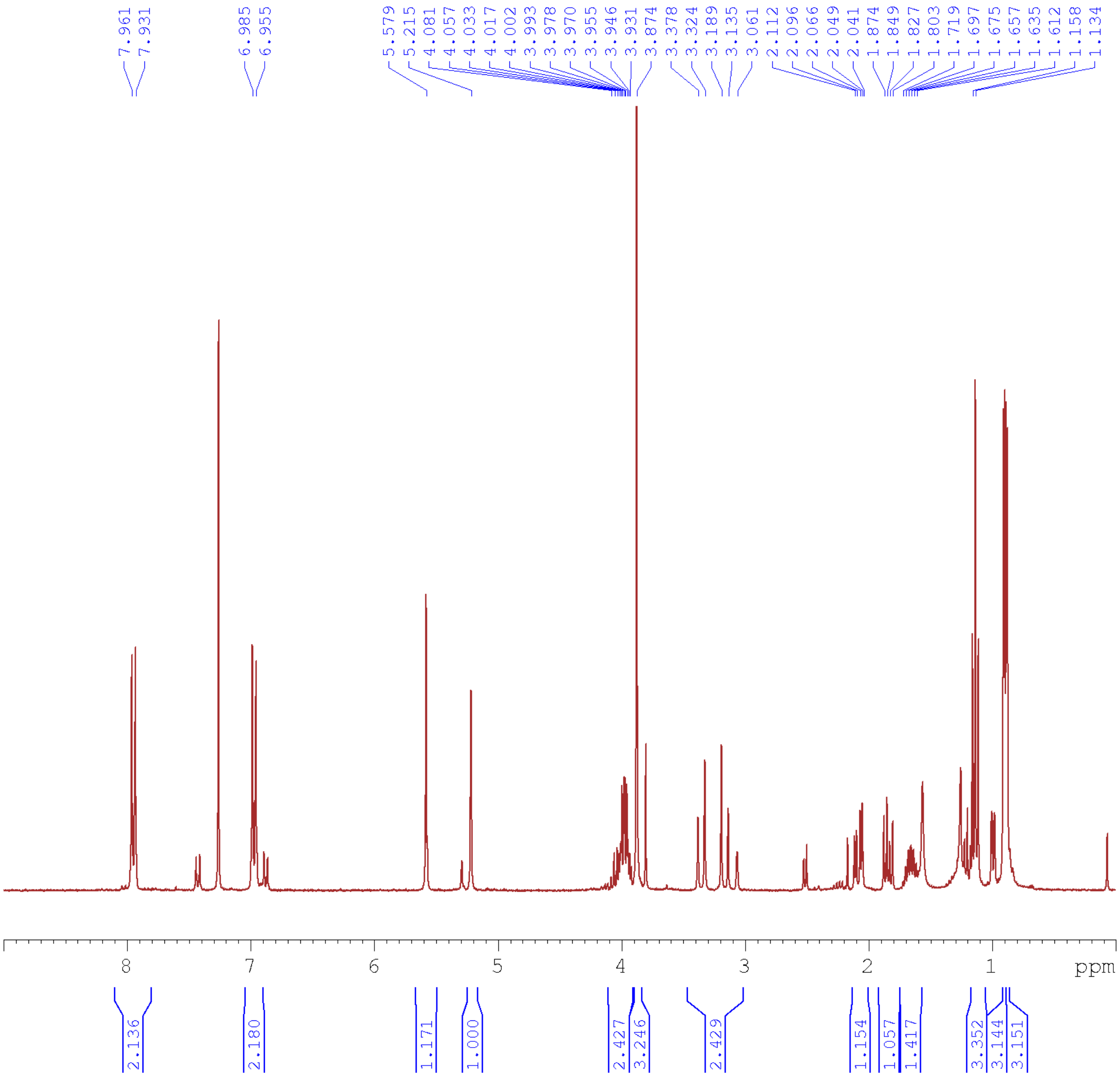

^{13}C NMR (75 MHz, $CDCl_3$, 298 K)

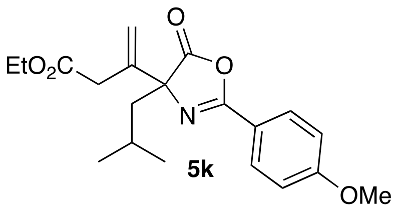

5k

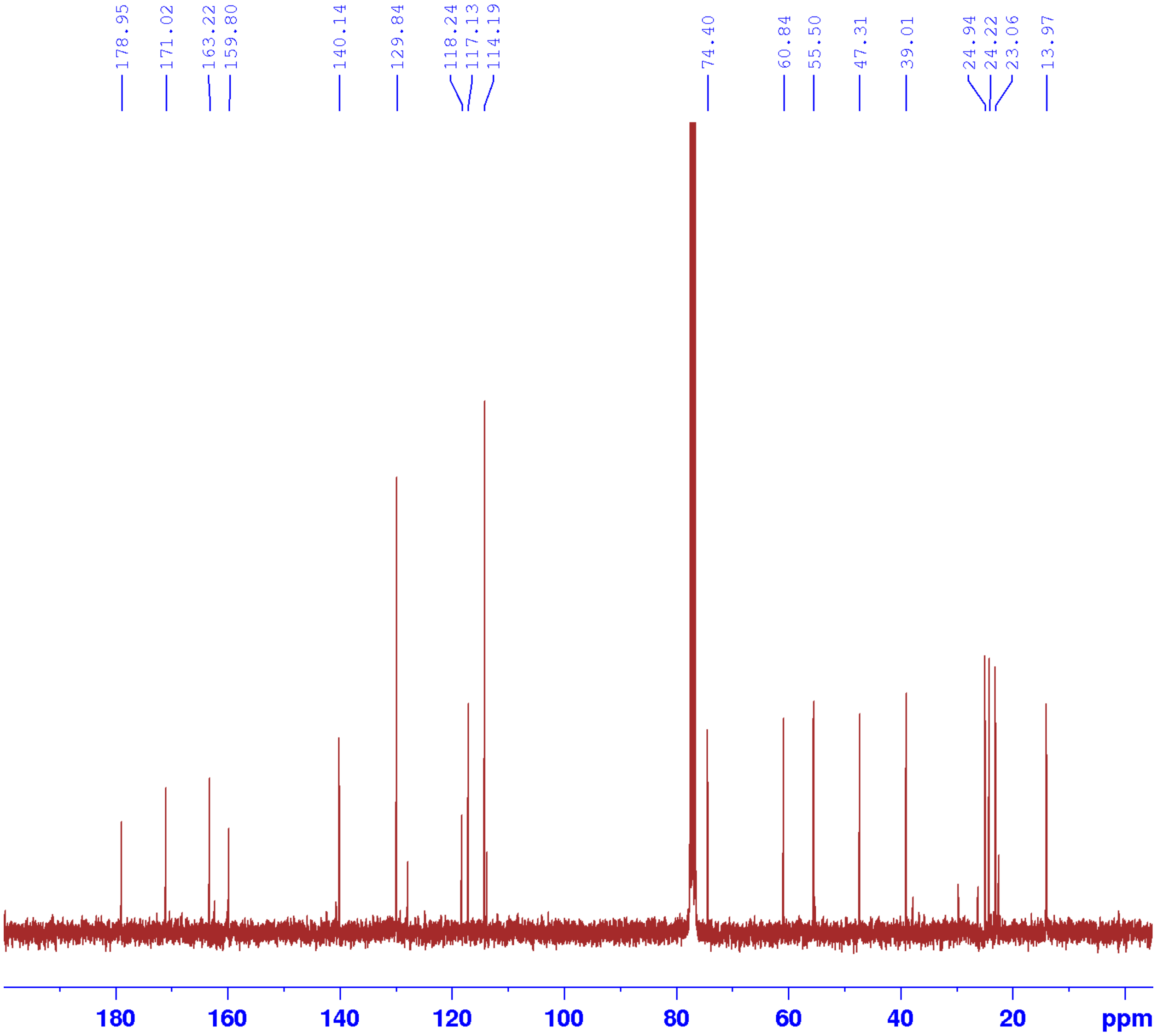

NMR spectra of compound 5l

^{1}H NMR (300 MHz, $CDCl_3$, 298 K)

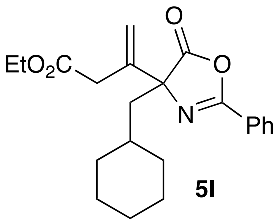

5l

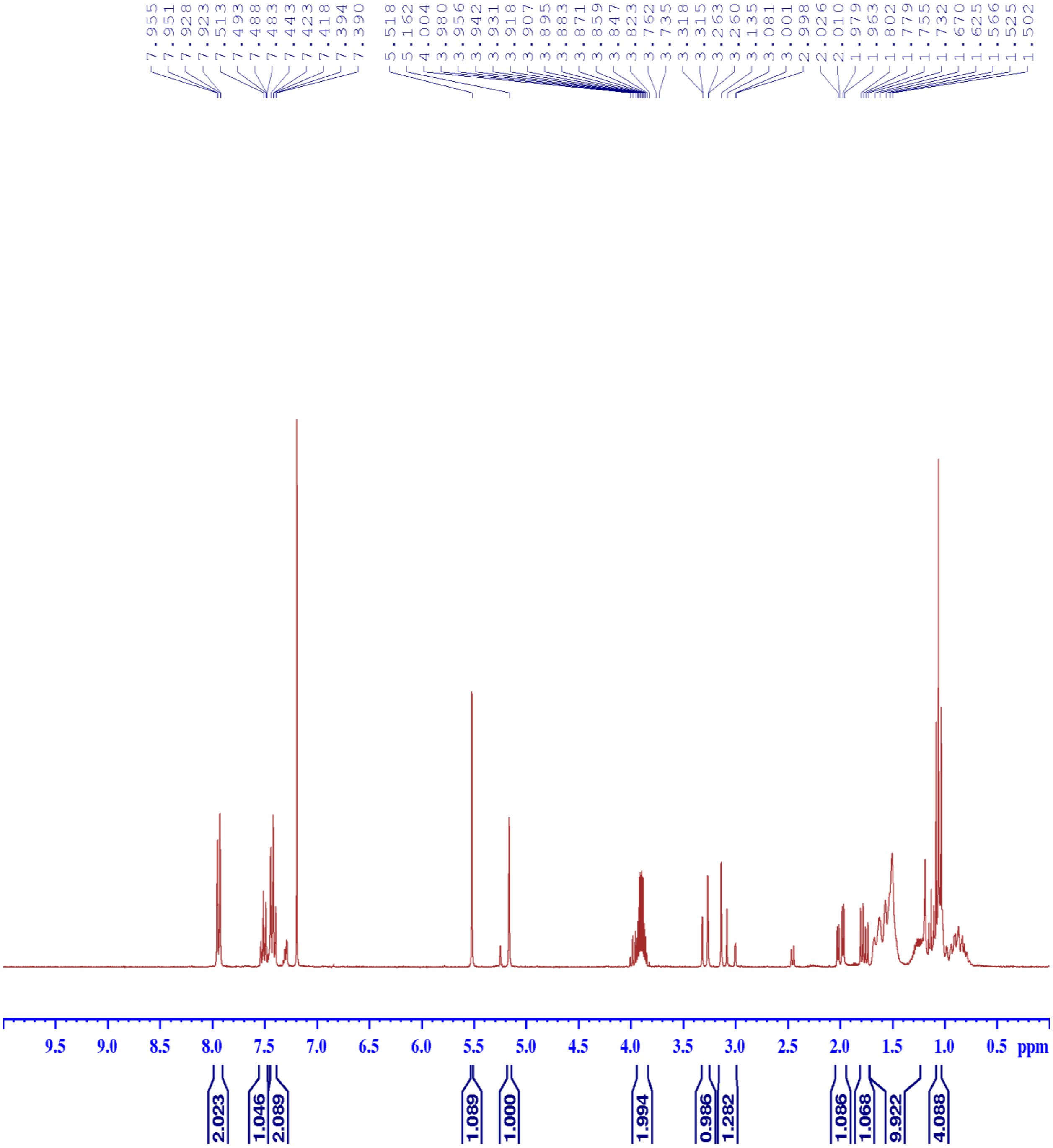

^{13}C NMR (75 MHz, $CDCl_3$, 298 K)

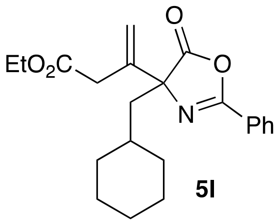

5l

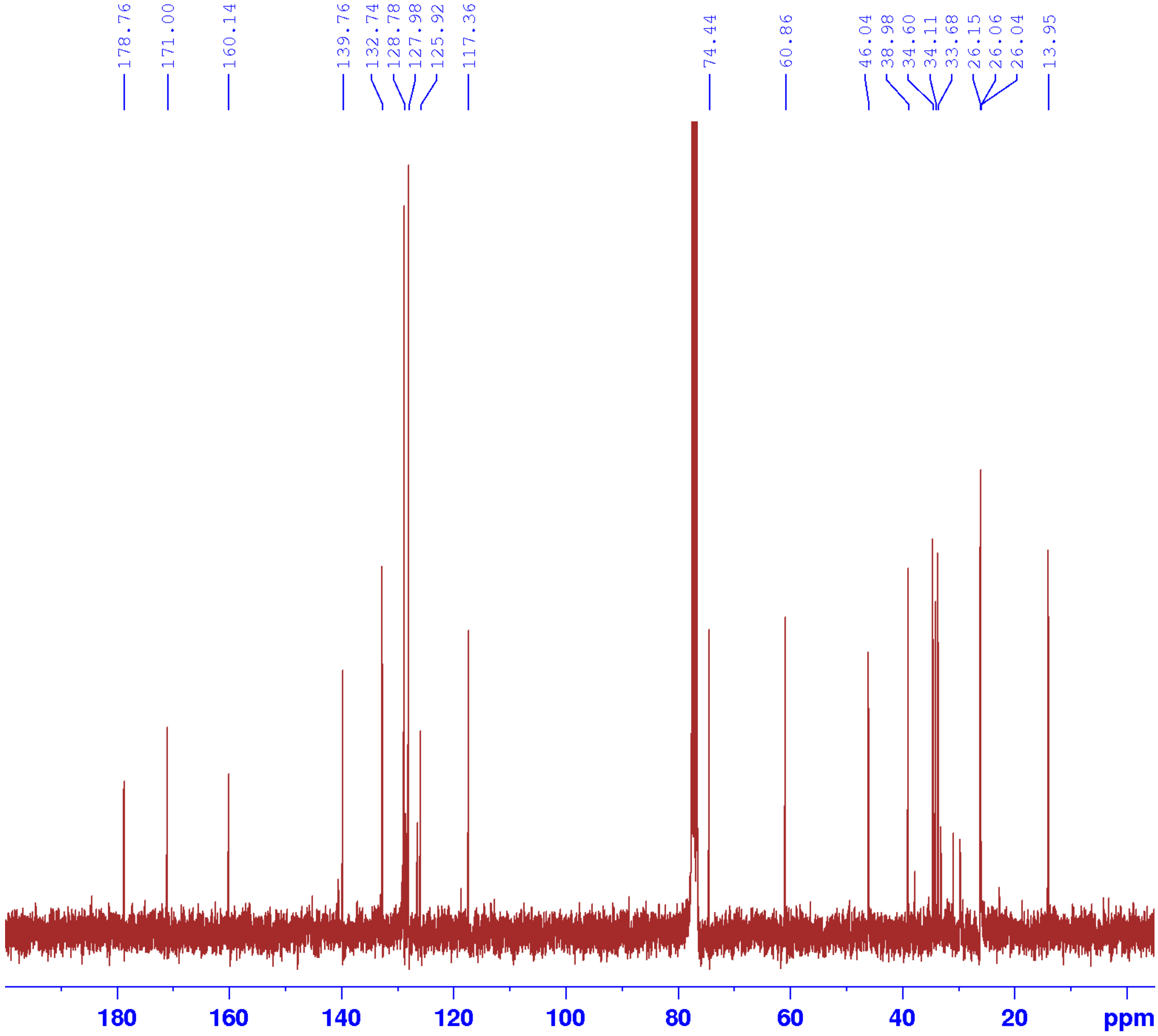

NMR spectra of compound 5n

^{1}H NMR (300 MHz, $CDCl_3$, 298 K)

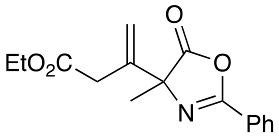

5n

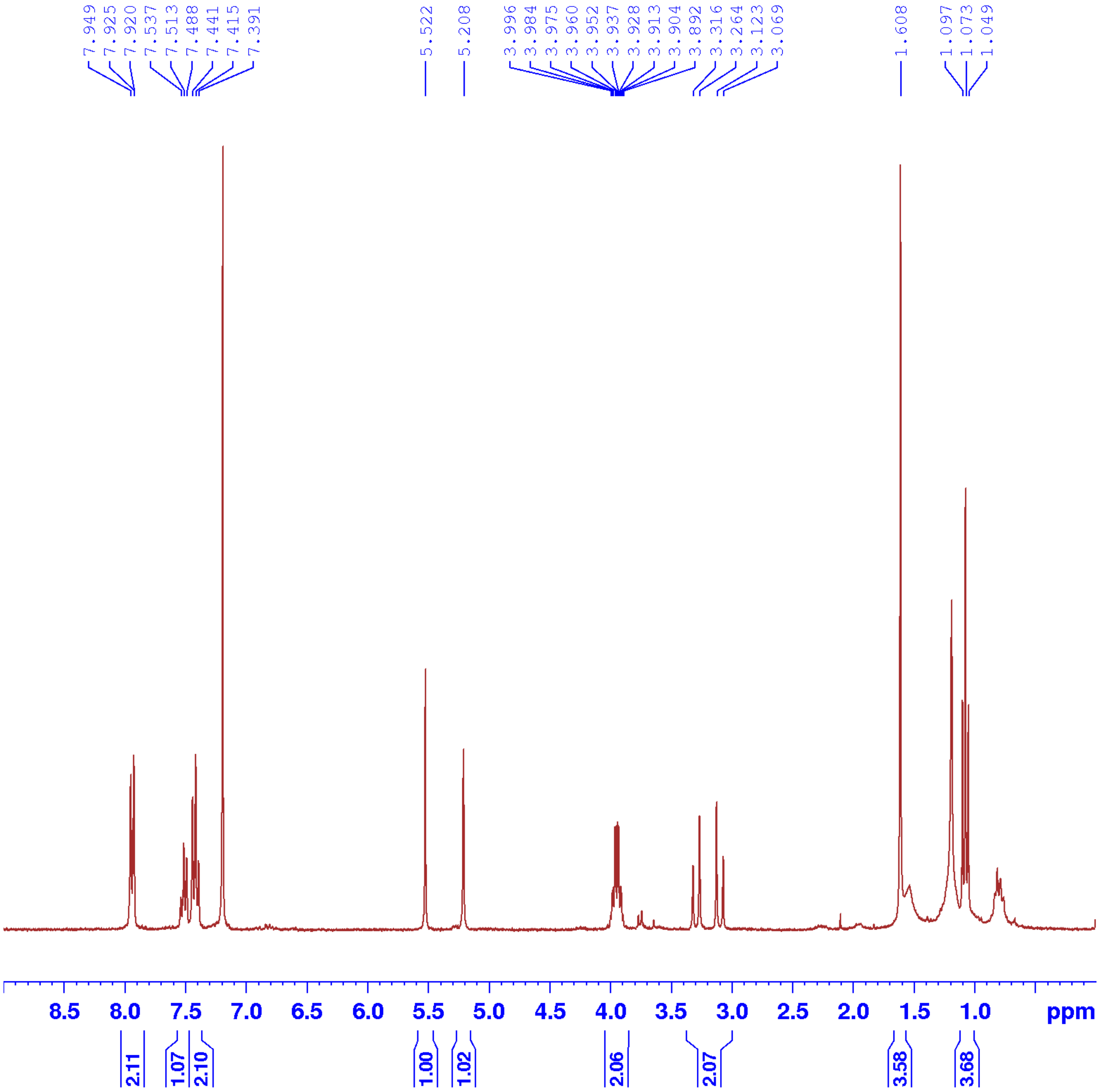

^{13}C NMR (75 MHz, $CDCl_3$, 298 K)

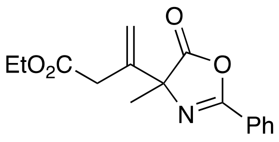

5n

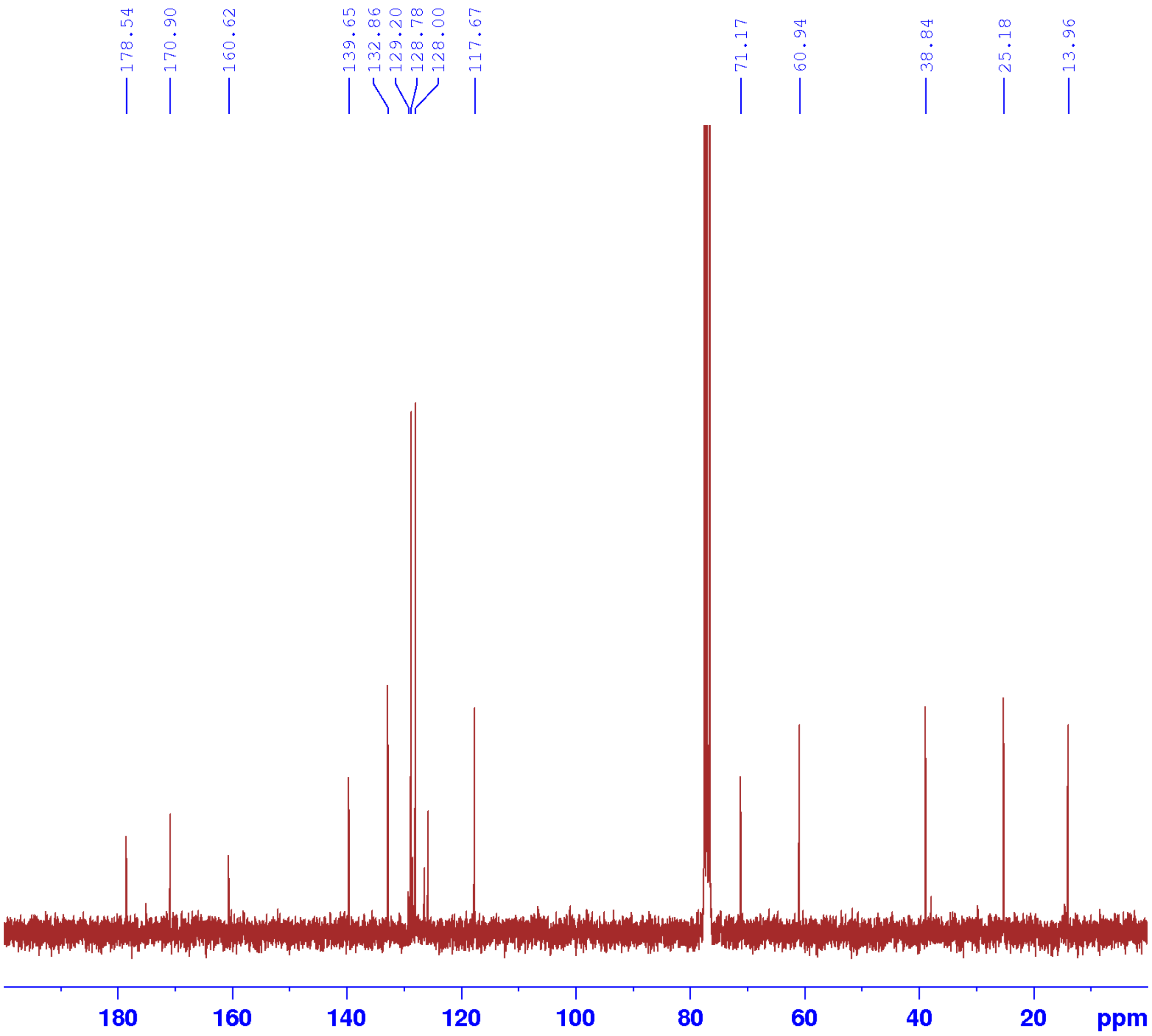

NMR spectra of compound 5o (*containing traces of ring-opened product*)

^{1}H NMR (300 MHz, $CDCl_3$, 298 K)

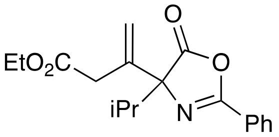

5o

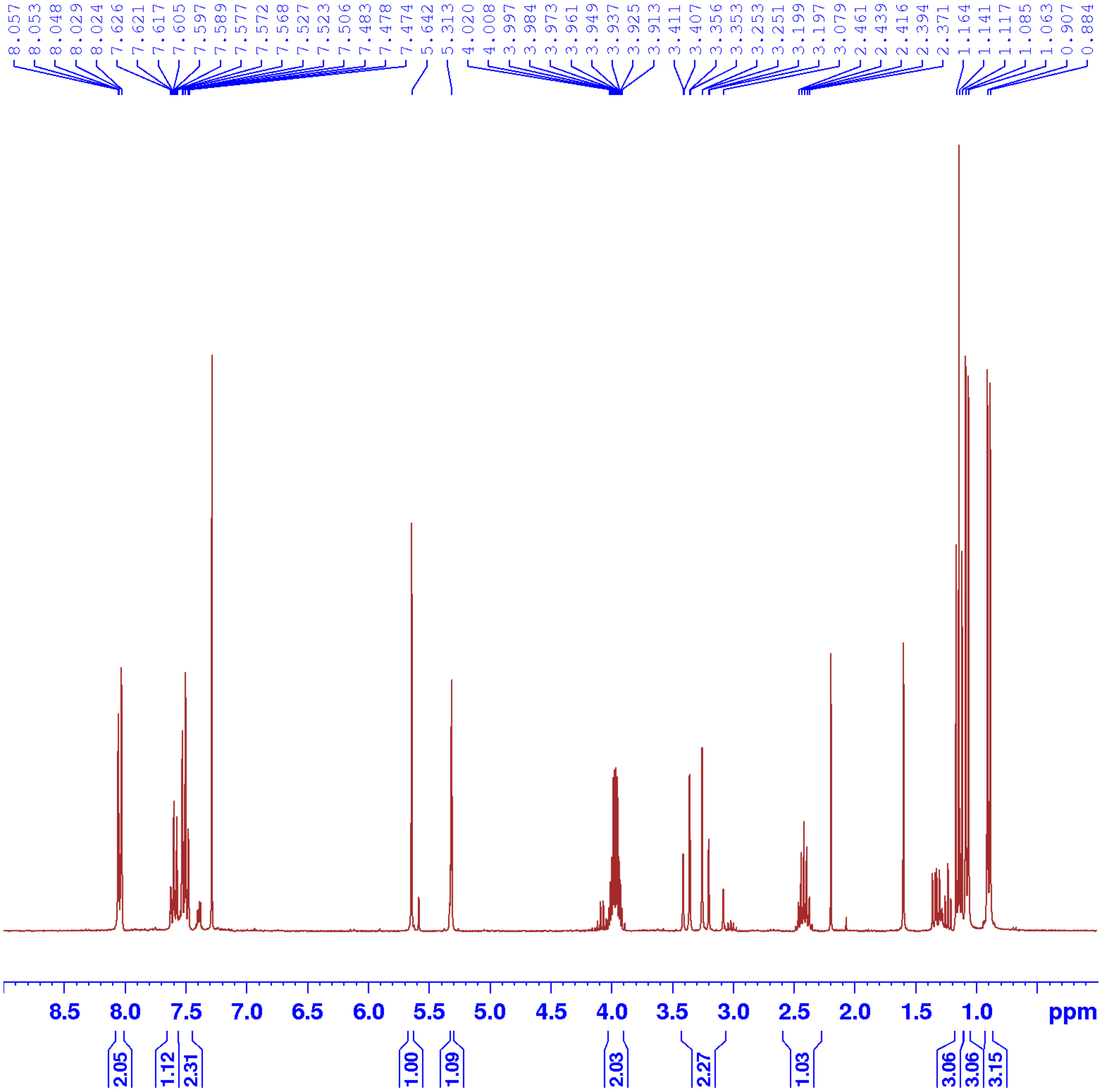

^{13}C NMR (75 MHz, $CDCl_3$, 298 K)

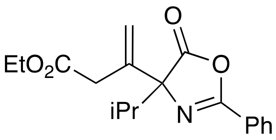

5o

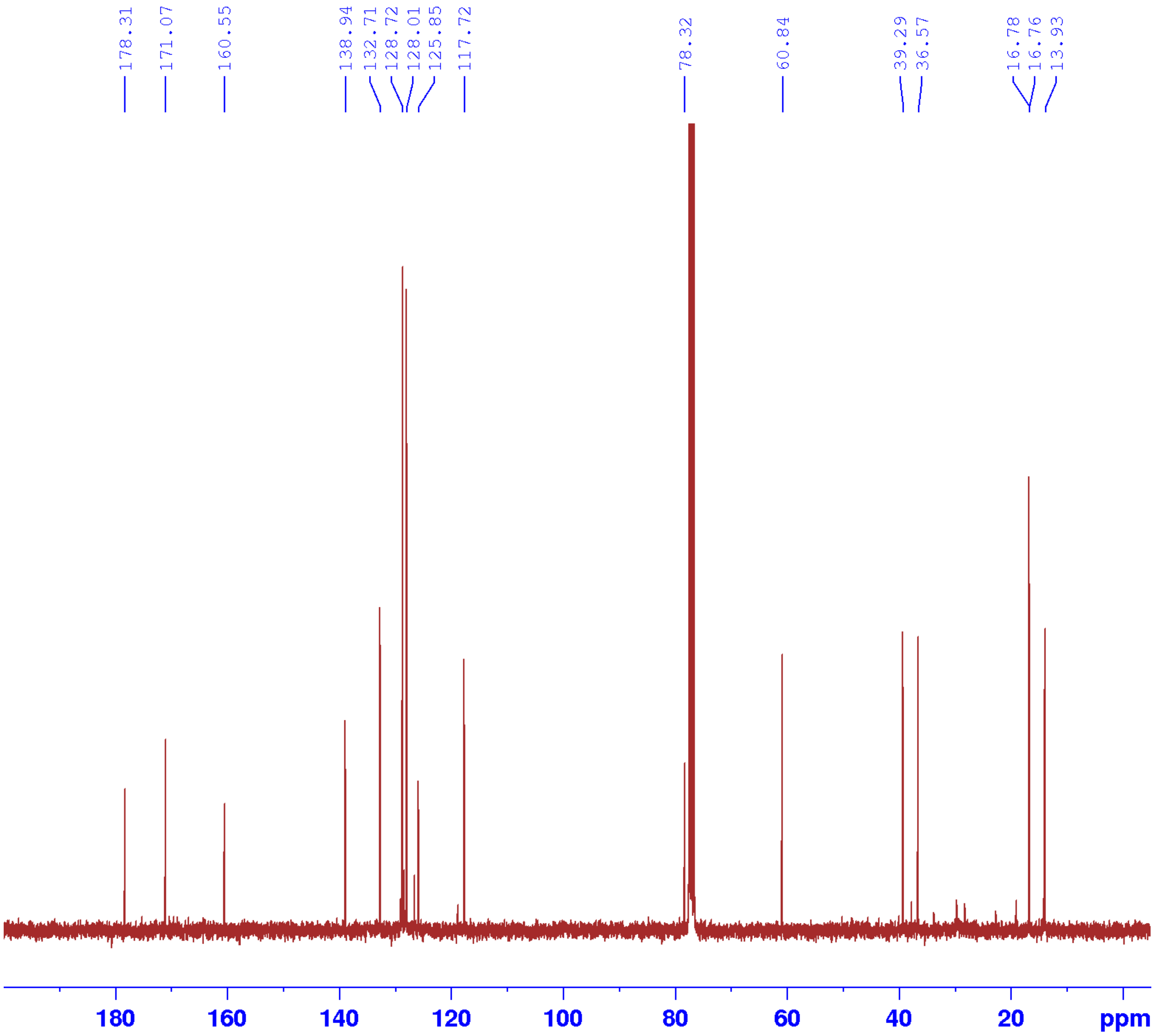

NMR spectra of compound 5p

^{1}H NMR (300 MHz, $CDCl_3$, 298 K)

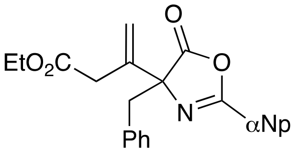

5p

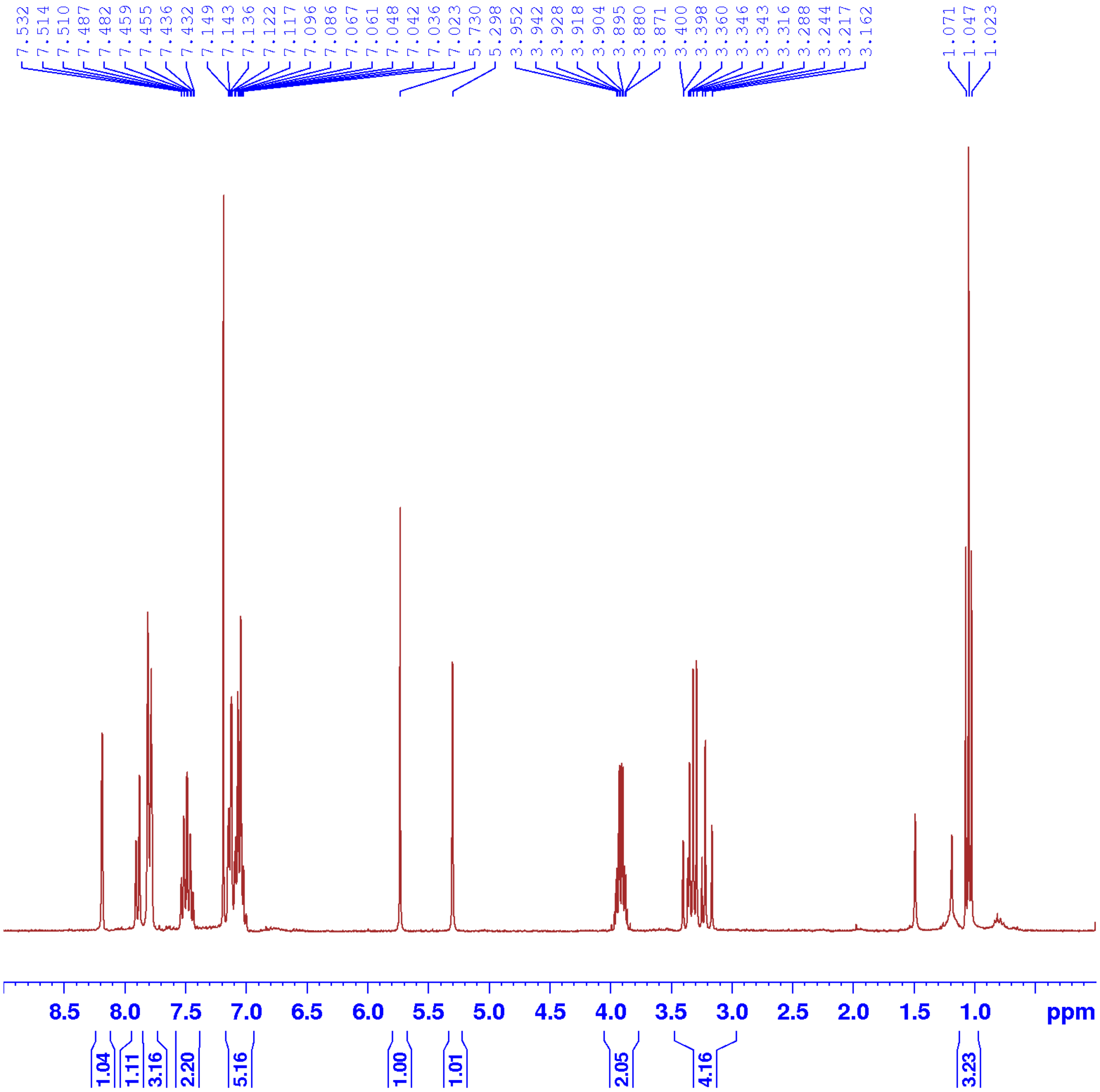

^{13}C NMR (75 MHz, $CDCl_3$, 298 K)

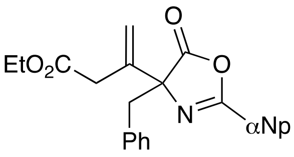

5p

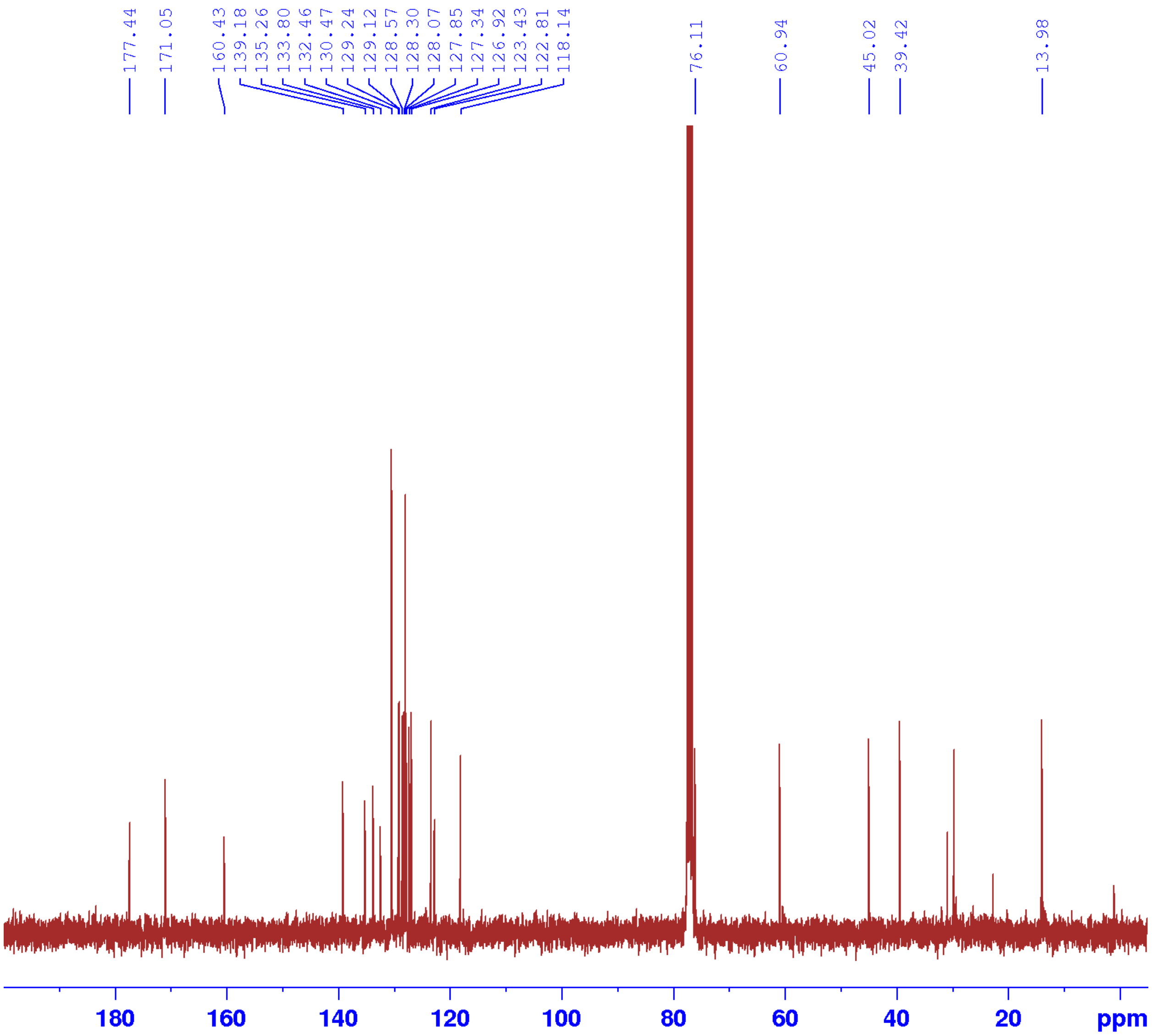

NMR spectra of compound 6a

^{1}H NMR (300 MHz, $CDCl_3$, 298 K)

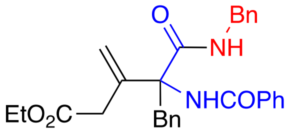

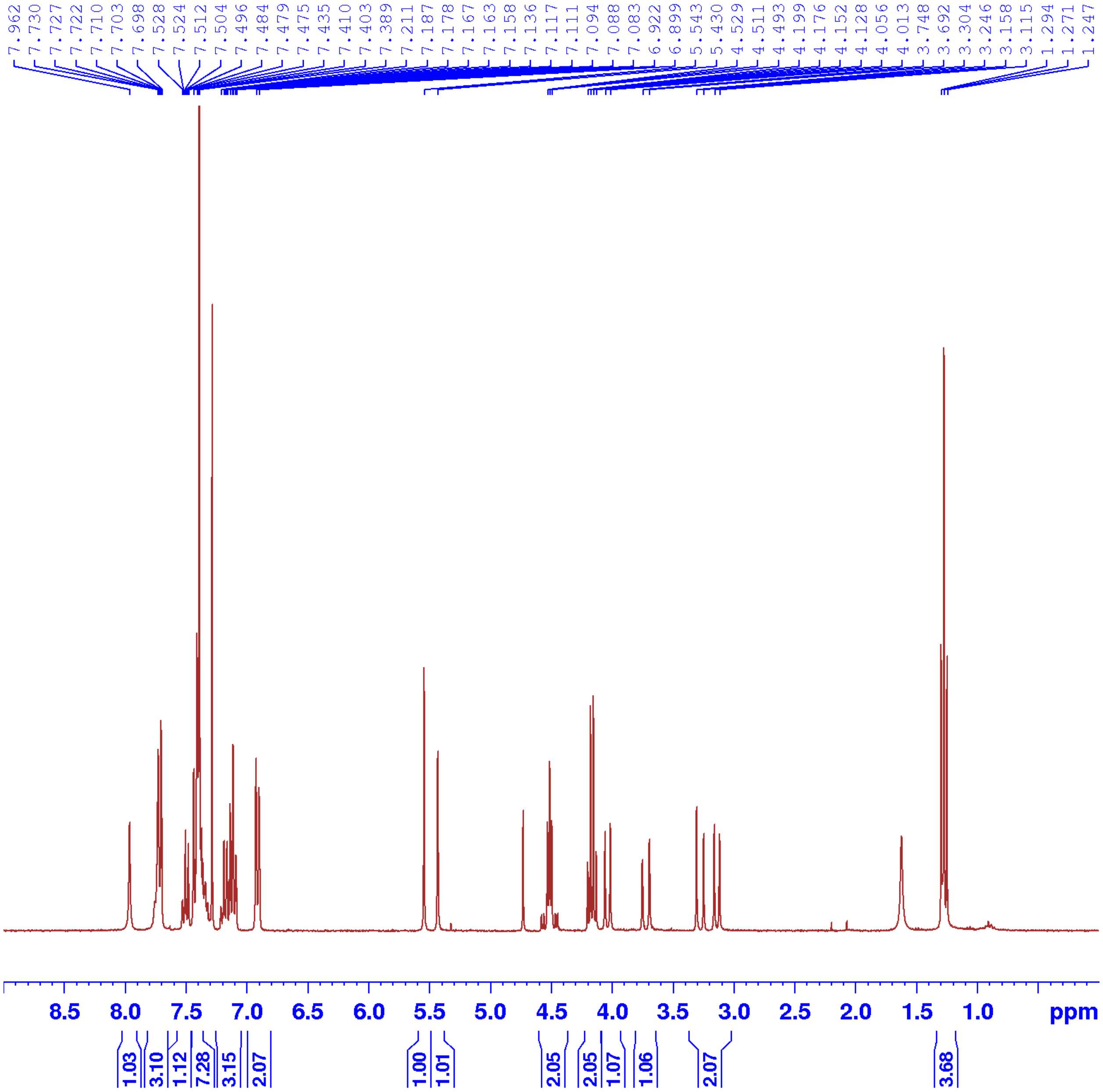

^{13}C NMR (75 MHz, $CDCl_3$, 298 K)

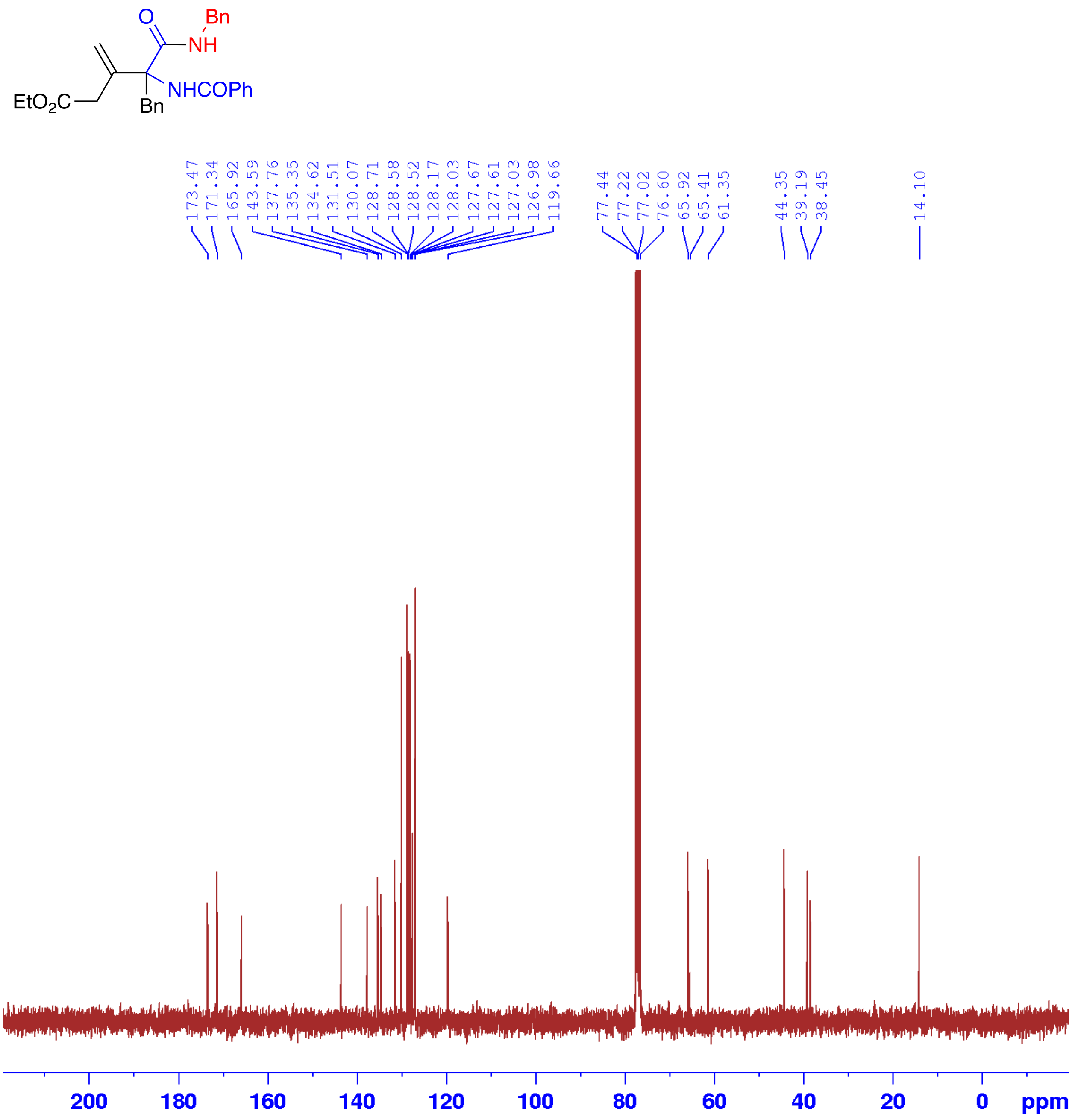

NMR spectra of compound 6b

^{1}H NMR (300 MHz, $CDCl_3$, 298 K)

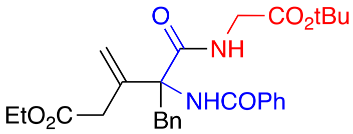

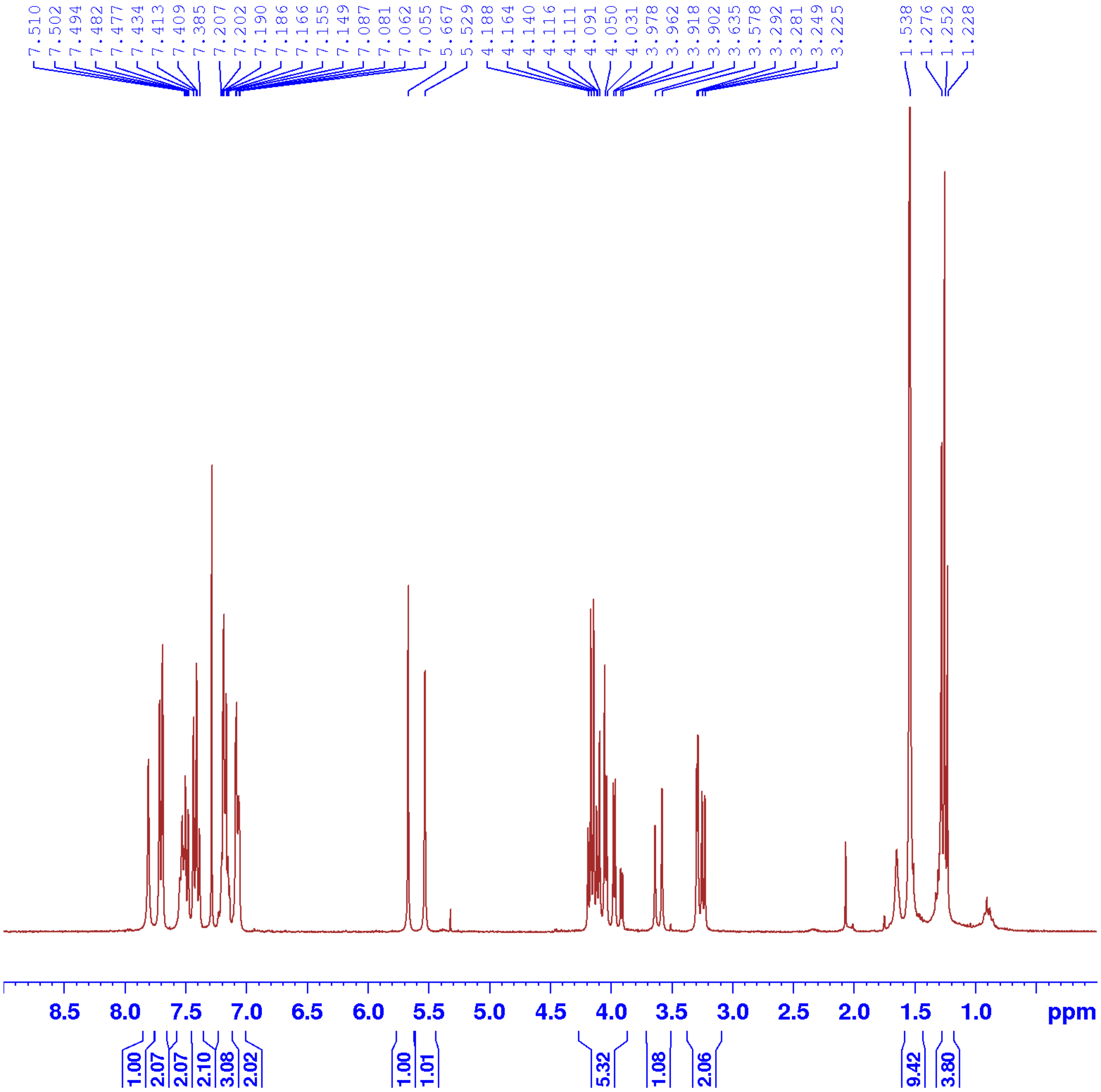

^{13}C NMR (75 MHz, $CDCl_3$, 298 K)

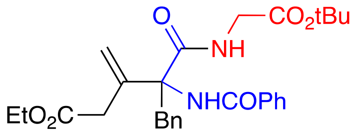

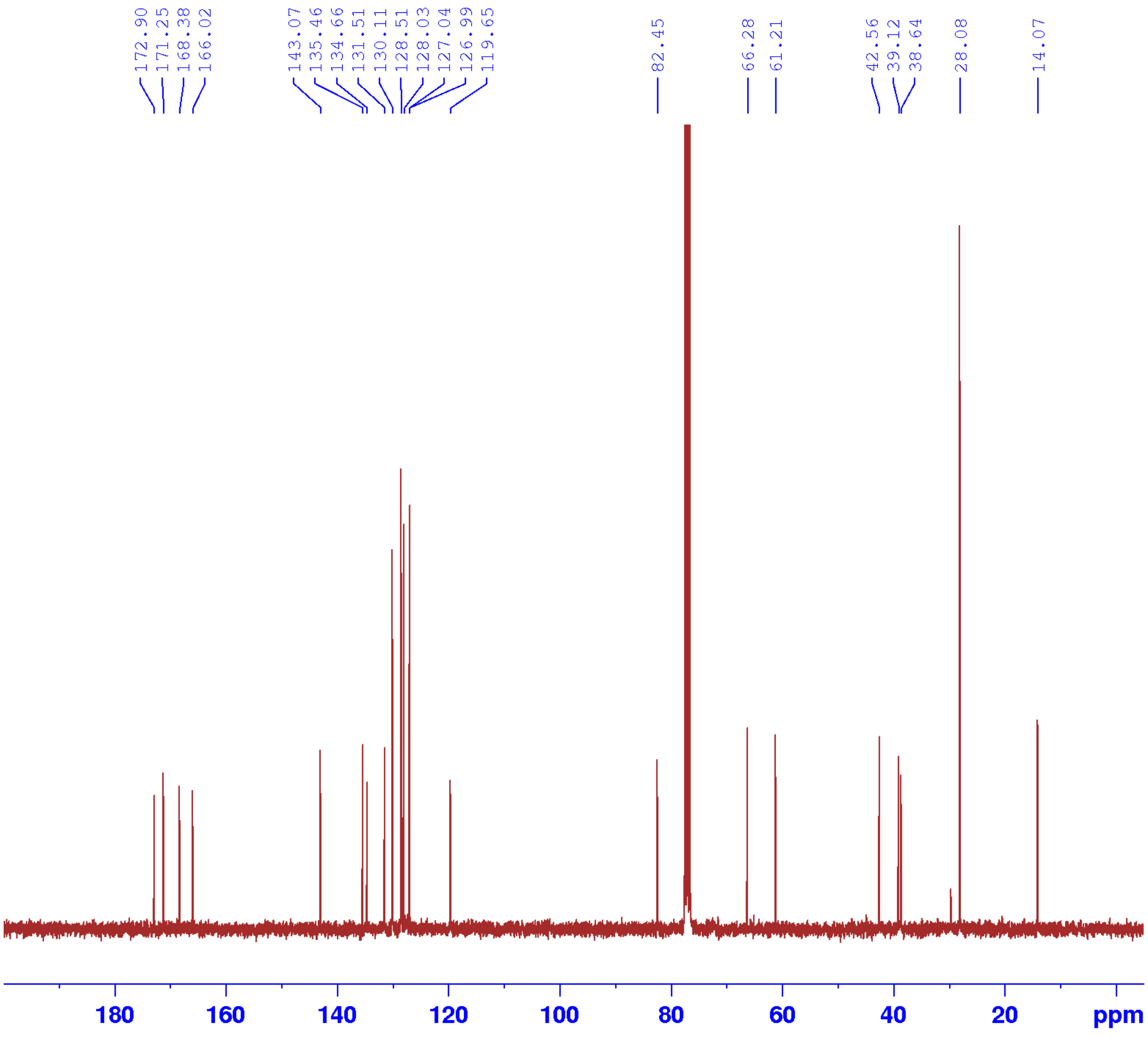

5. HPLC Chromatograms

HPLC traces of compound 5a

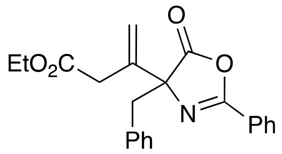

5a

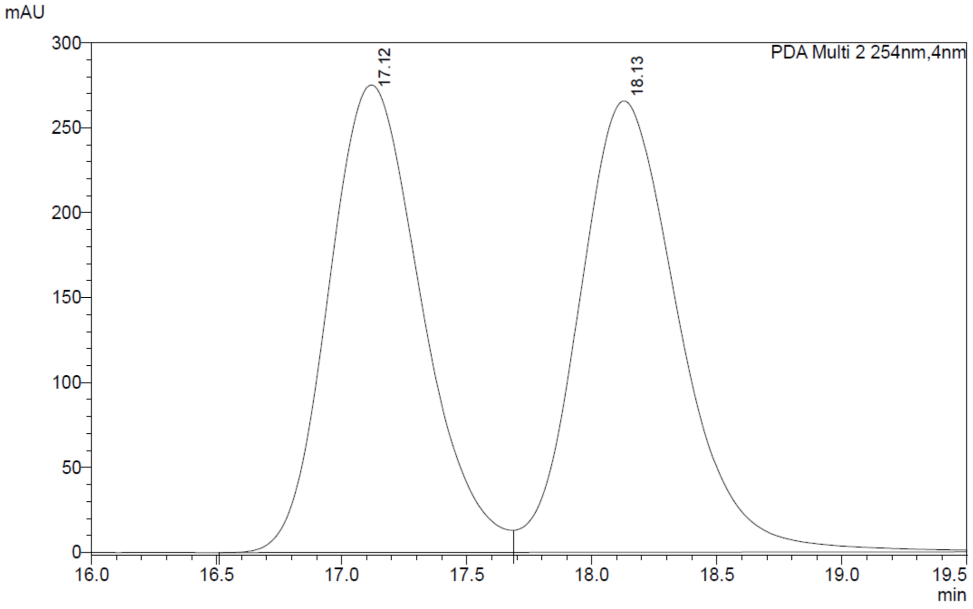

Peak Table

PDA Ch2 254nm

Peak#	Ret. Time	Area	Area%
1	17.12	7090681	48.98
2	18.13	7387342	51.02
Total		14478023	100.00

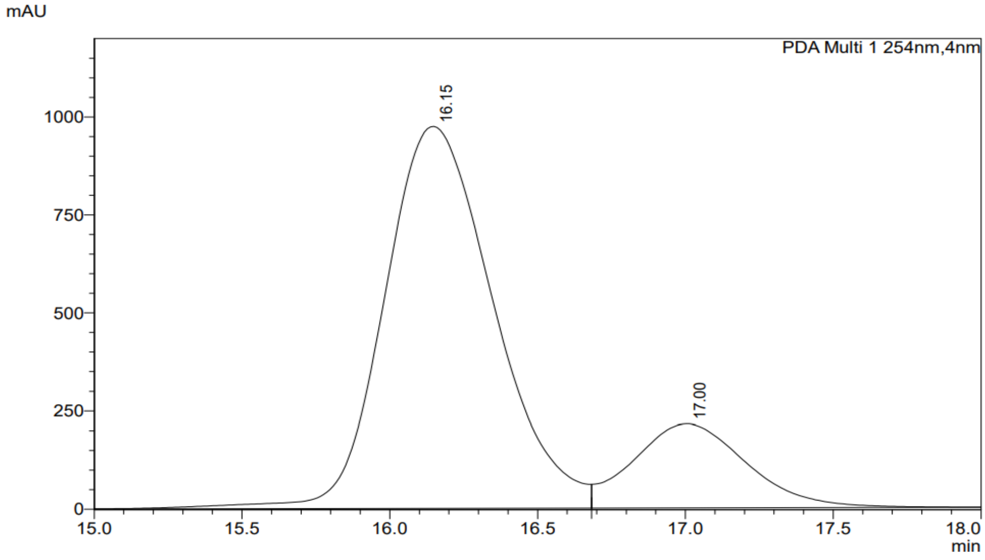

Peak Table

PDA Ch1 254nm

Peak#	Ret. Time	Area	Area%
1	16.15	24915611	81.41
2	17.00	5689961	18.59
Total		30605572	100.00

HPLC traces of compound 5b

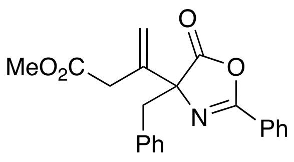

5b

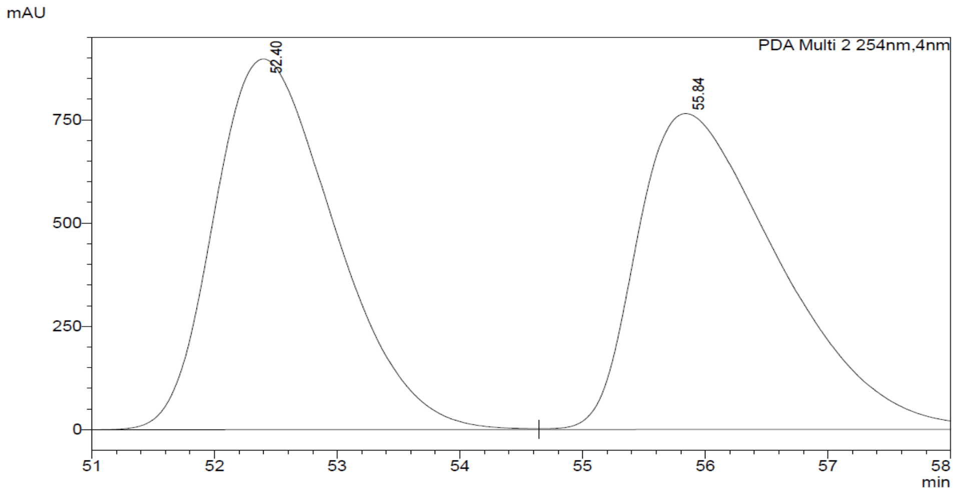

Peak Table

PDA Ch2 254nm

Peak#	Ret. Time	Area	Area%
1	52.40	61451526	49.72
2	55.84	62152914	50.28
Total		123604440	100.00

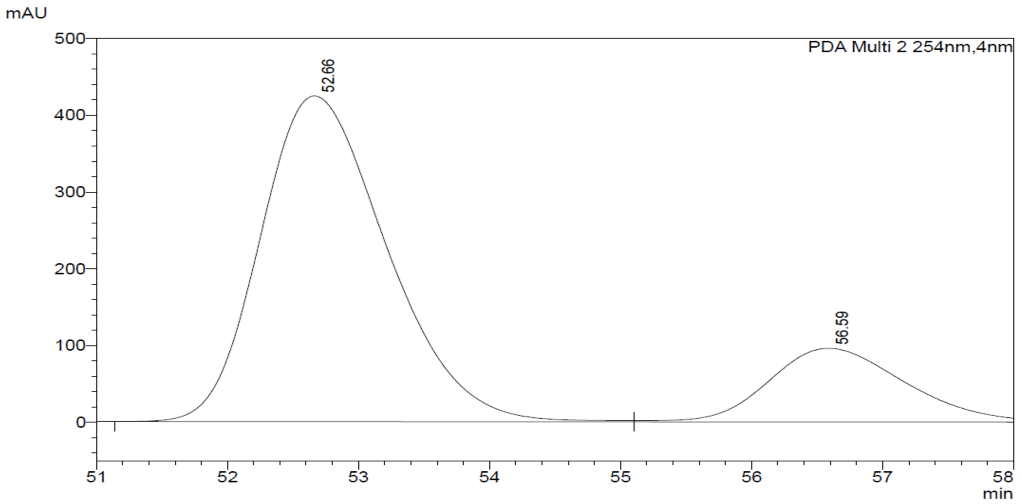

Peak Table

PDA Ch2 254nm

Peak#	Ret. Time	Area	Area%
1	52.66	28371001	80.41
2	56.59	6913381	19.59
Total		35284382	100.00

<u>HPLC traces of compound 5c</u>

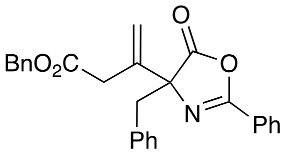

5c

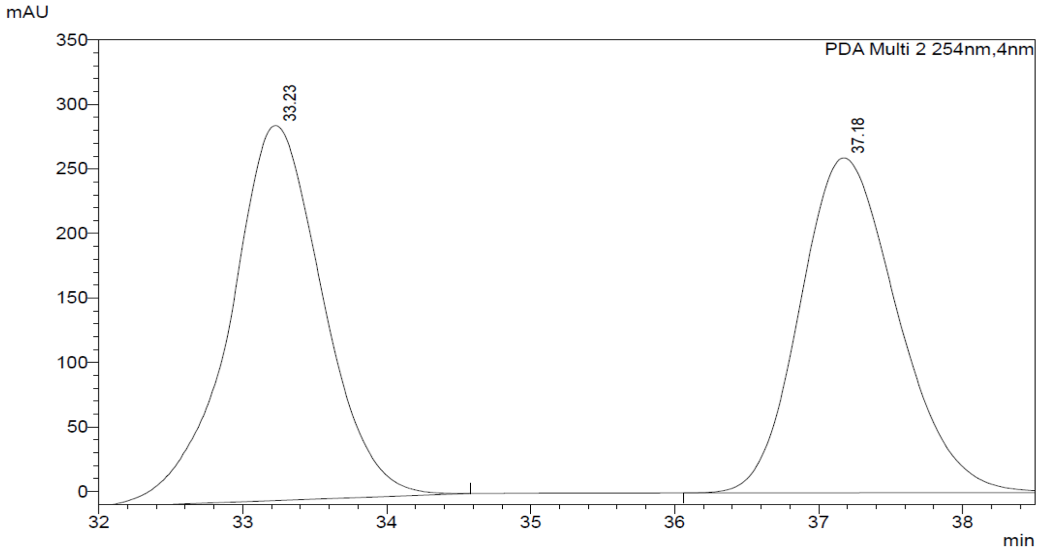

Peak Table

PDA Ch2 254nm

Peak#	Ret. Time	Area	Area%
1	33.23	13050646	51.61
2	37.18	12234954	48.39
Total		25285600	100.00

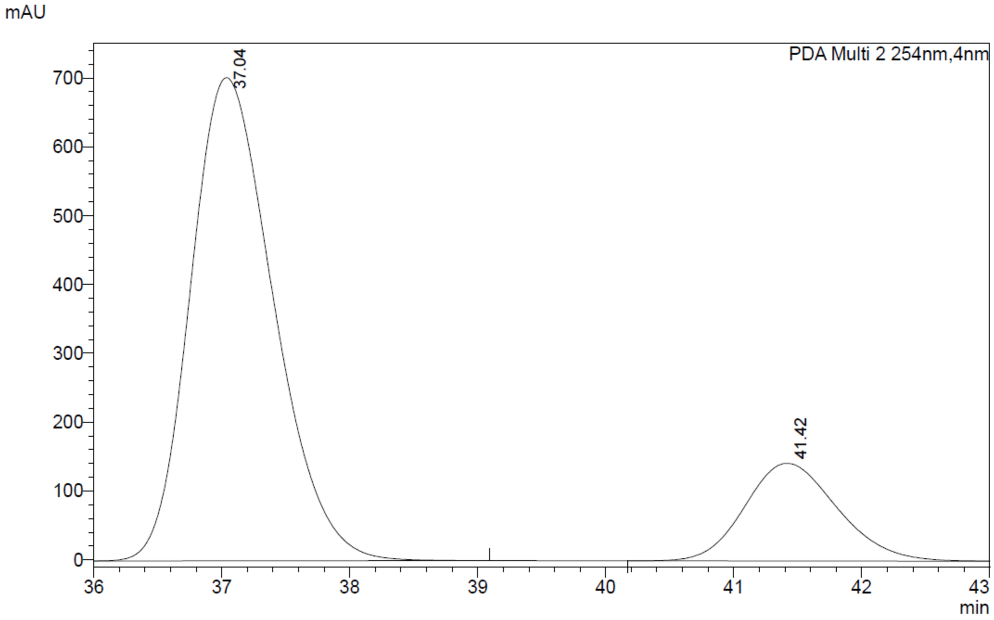

Peak Table

PDA Ch2 254nm

Peak#	Ret. Time	Area	Area%
1	37.04	31666267	81.58
2	41.42	7149212	18.42
Total		38815479	100.00

HPLC traces of compound 5d

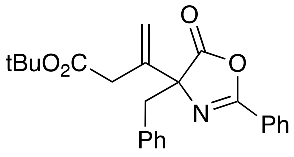

5d

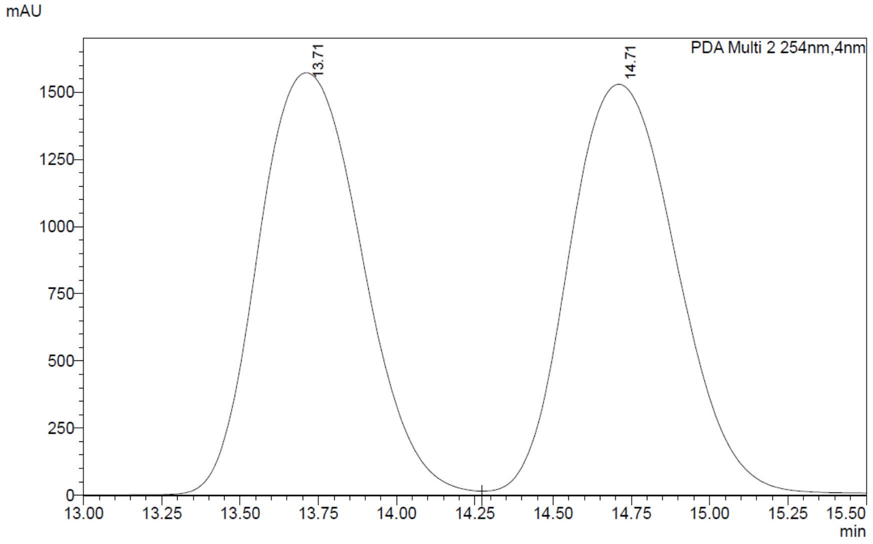

Peak Table

PDA Ch2 254nm

Peak#	Ret. Time	Area	Area%
1	13.71	36239310	49.23
2	14.71	37369328	50.77
Total		73608638	100.00

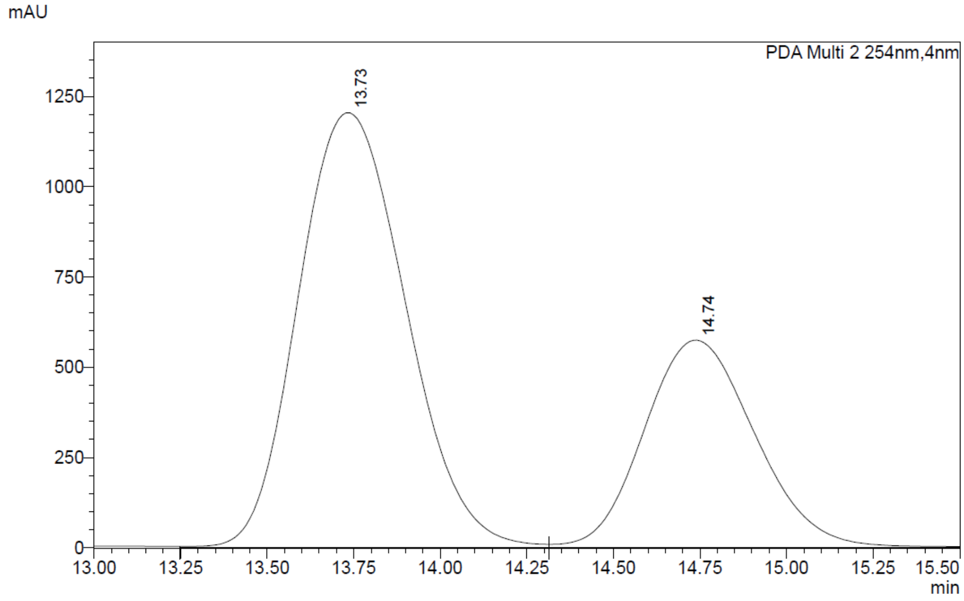

Peak Table

PDA Ch2 254nm

Peak#	Ret. Time	Area	Area%
1	13.73	26022533	66.86
2	14.74	12898901	33.14
Total		38921434	100.00

<u>HPLC traces of compound 5e</u>

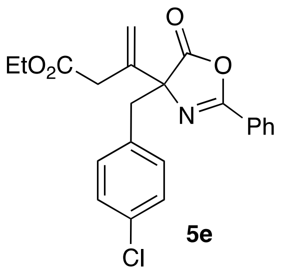

5e

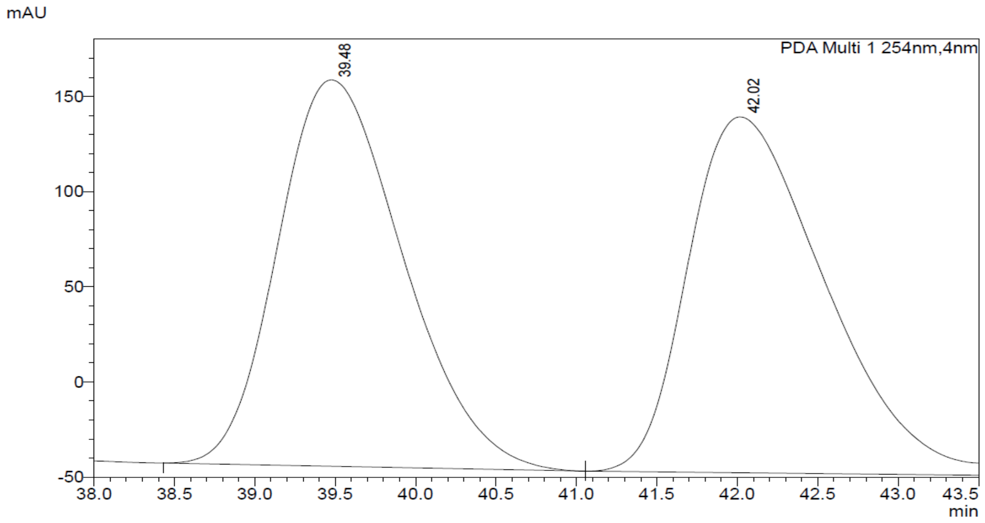

Peak Table

PDA Ch1 254nm

Peak#	Ret. Time	Area	Area%
1	39.48	11003087	49.96
2	42.02	11022192	50.04
Total		22025279	100.00

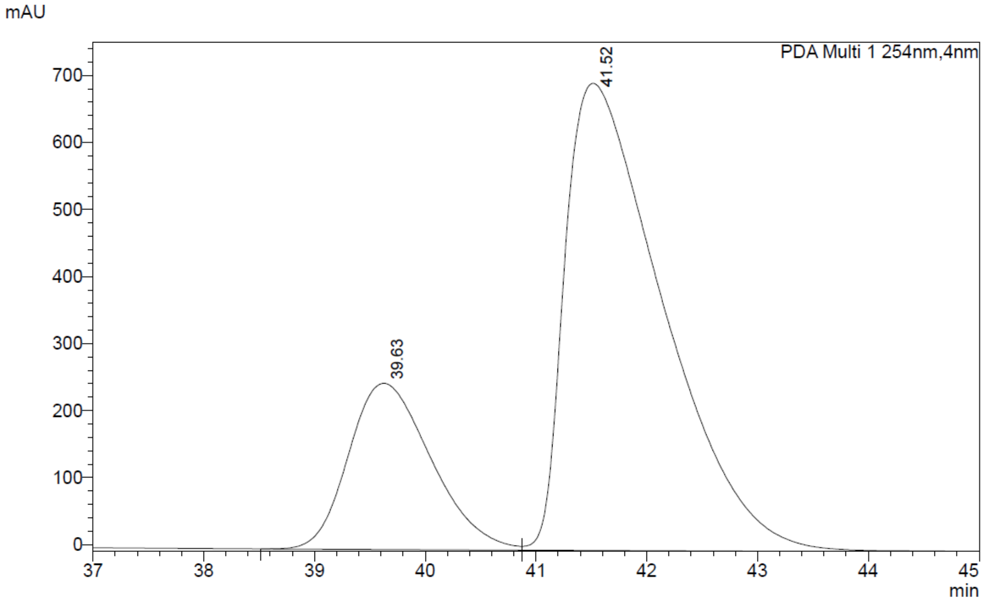

Peak Table

PDA Ch1 254nm

Peak#	Ret. Time	Area	Area%
1	39.63	12888472	23.05
2	41.52	43032898	76.95
Total		55921370	100.00

HPLC traces of compound 5f

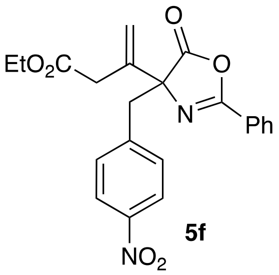

5f

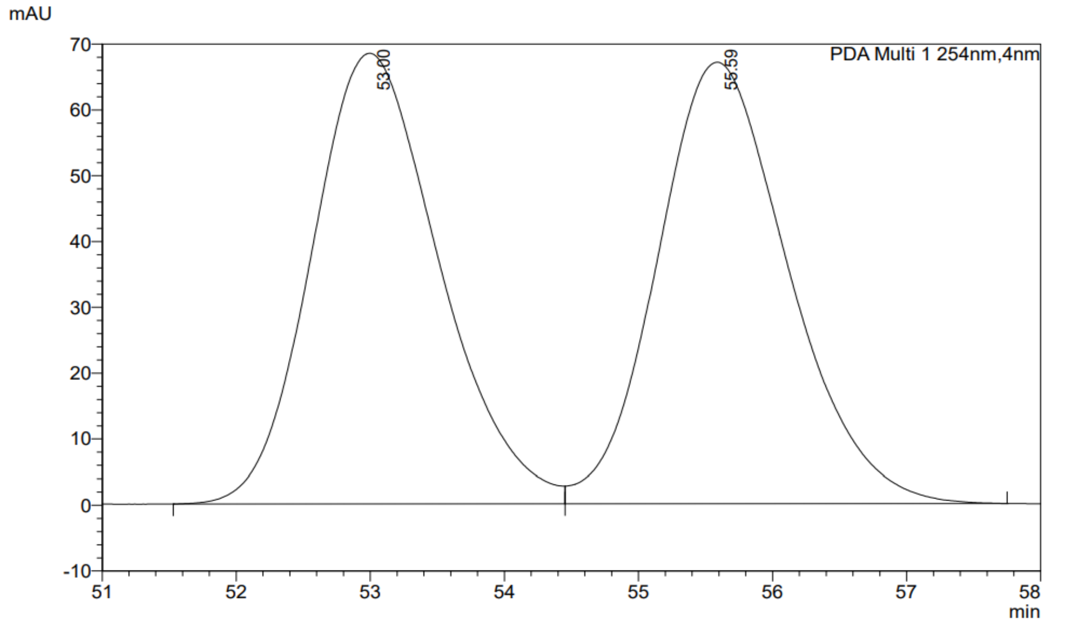

Peak Table

PDA Ch1 254nm

Peak#	Ret. Time	Area	Area%
1	53.00	4512895	49.85
2	55.59	4539681	50.15
Total		9052576	100.00

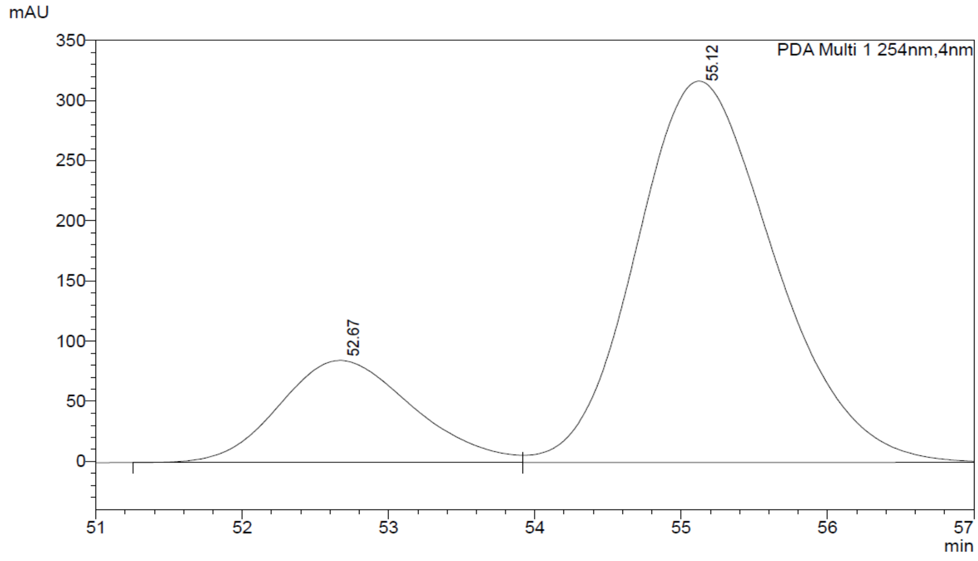

Peak Table

PDA Ch1 254nm

Peak#	Ret. Time	Area	Area%
1	52.67	5420086	20.54
2	55.12	20967253	79.46
Total		26387339	100.00

HPLC traces of compound 5g

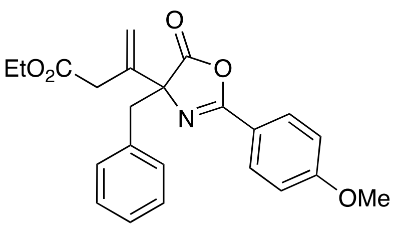

5g

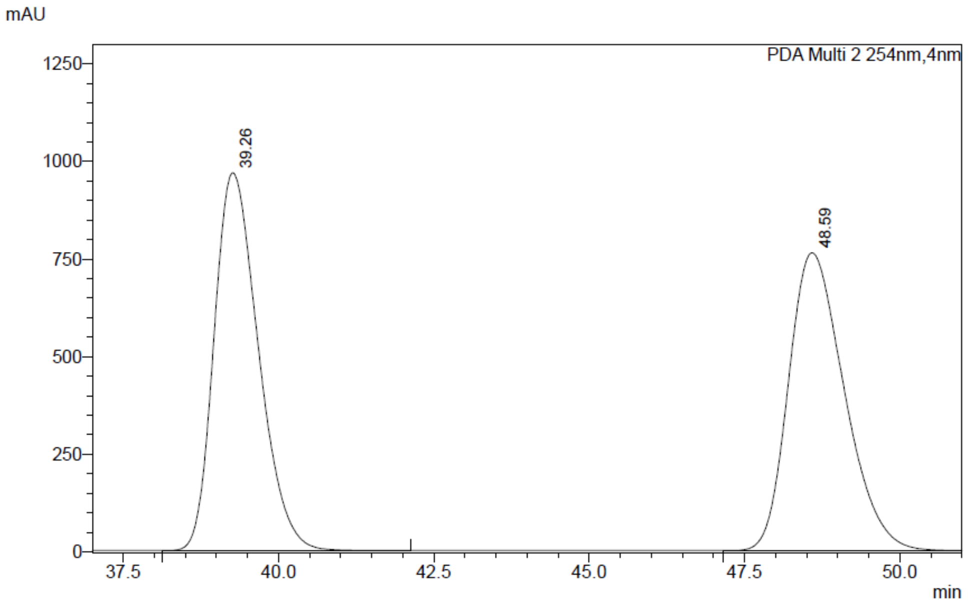

Peak Table

PDA Ch2 254nm

Peak#	Ret. Time	Area	Area%
1	39.26	47885898	50.13
2	48.59	47646696	49.87
Total		95532594	100.00

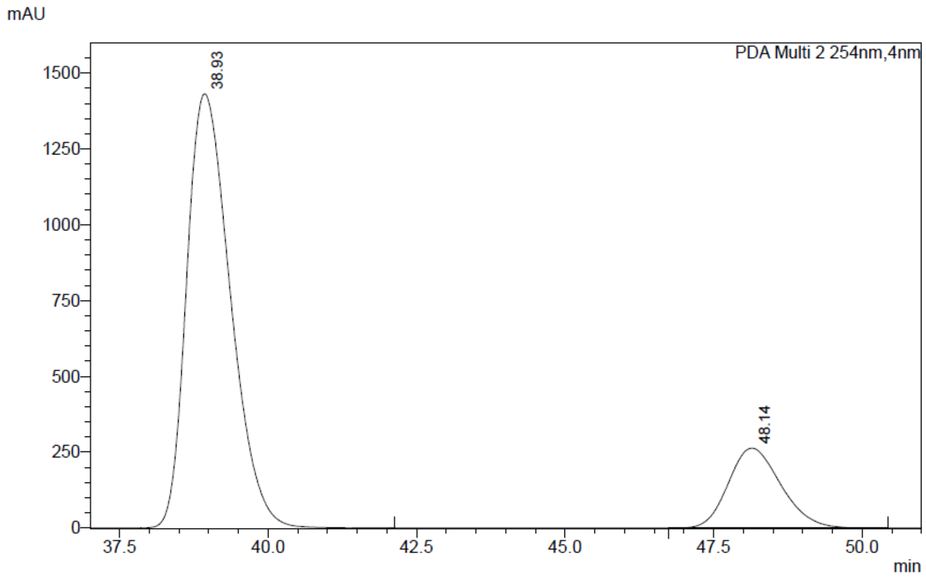

Peak Table

PDA Ch2 254nm

Peak#	Ret. Time	Area	Area%
1	38.93	73851534	81.96
2	48.14	16253845	18.04
Total		90105379	100.00

<u>**HPLC traces of compound 5h**</u>

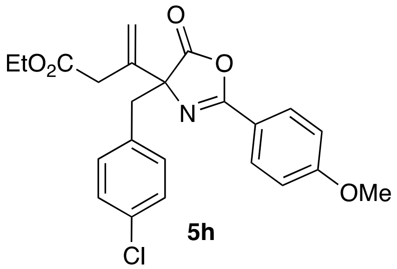

5h

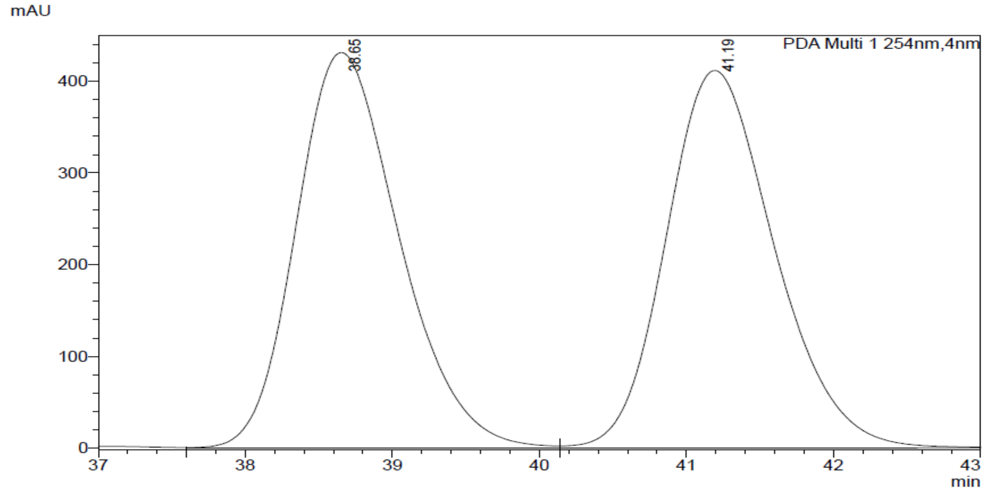

Peak Table

PDA Ch1 254nm

Peak#	Ret. Time	Area	Area%
1	38.65	21021081	49.93
2	41.19	21081792	50.07
Total		42102873	100.00

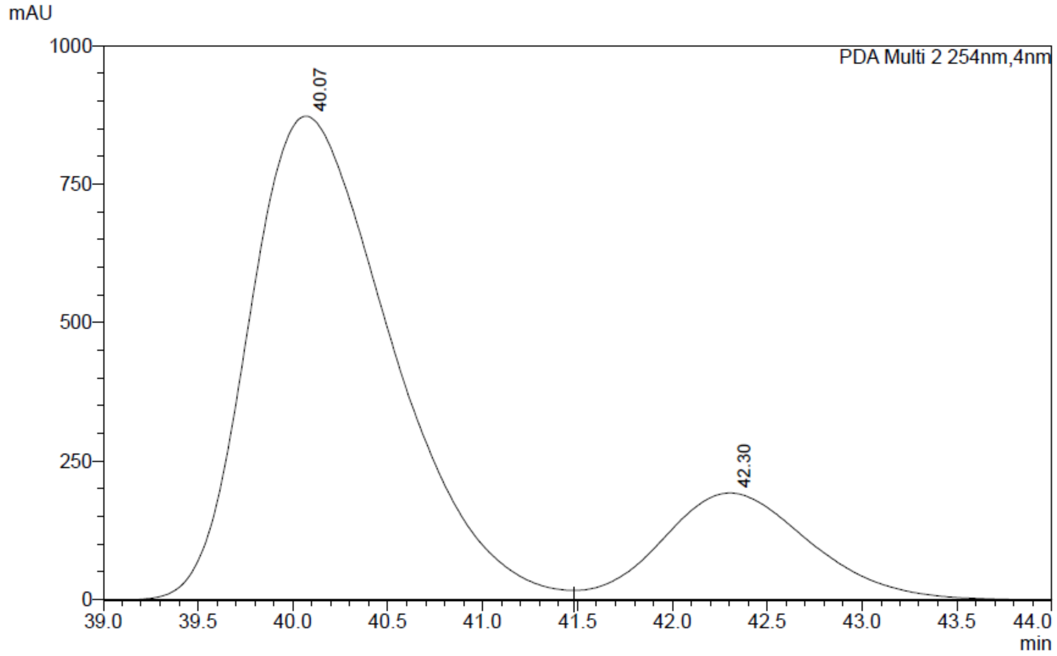

Peak Table

PDA Ch2 254nm

Peak#	Ret. Time	Area	Area%
1	40.07	45563752	81.18
2	42.30	10559991	18.82
Total		56123743	100.00

HPLC traces of compound 5i

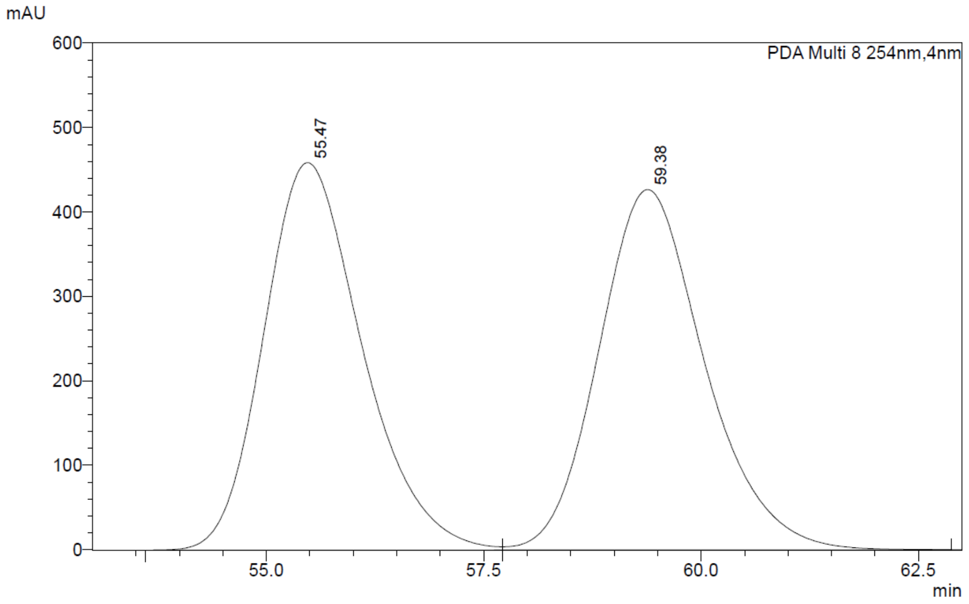

Peak Table

PDA Ch8 254nm

Peak#	Ret. Time	Area	Area%
1	55.47	36122440	49.97
2	59.38	36164191	50.03
Total		72286631	100.00

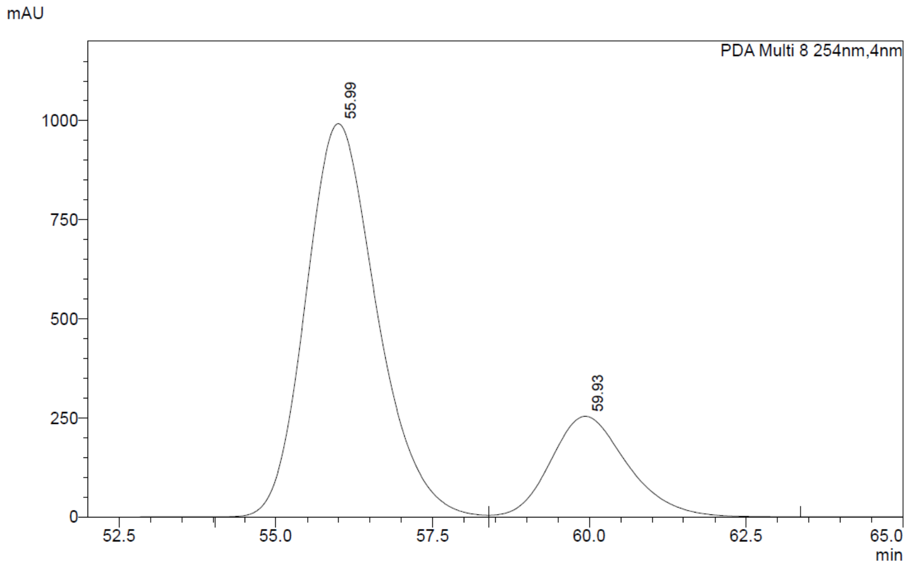

Peak Table

PDA Ch8 254nm

Peak#	Ret. Time	Area	Area%
1	55.99	78354208	78.33
2	59.93	21670718	21.67
Total		100024926	100.00

HPLC traces of compound 5j

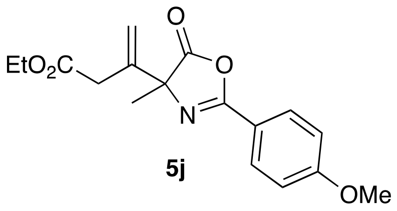

5j

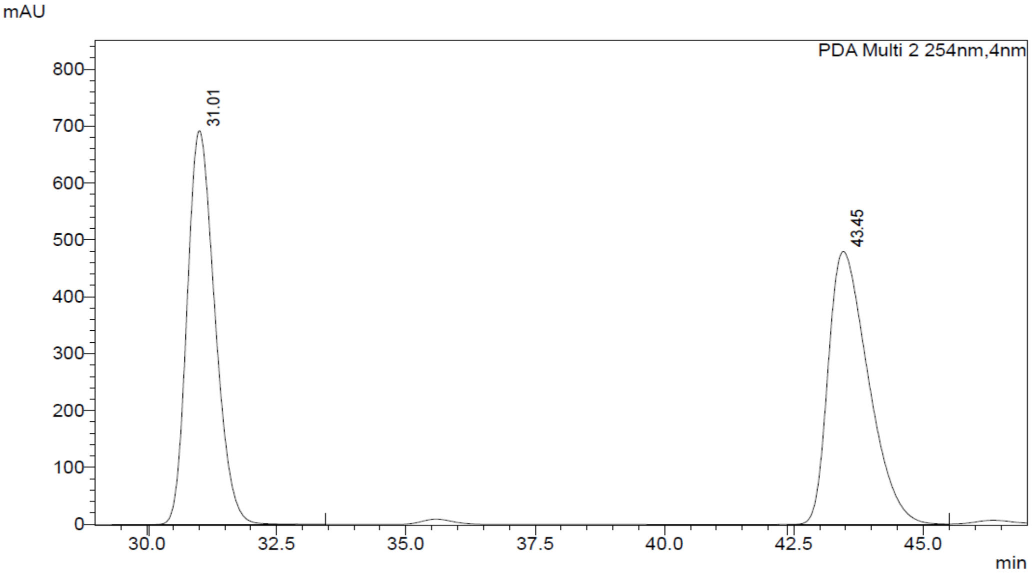

Peak Table

PDA Ch2 254nm

Peak#	Ret. Time	Area	Area%
1	31.01	25809245	49.95
2	43.45	25859036	50.05
Total		51668281	100.00

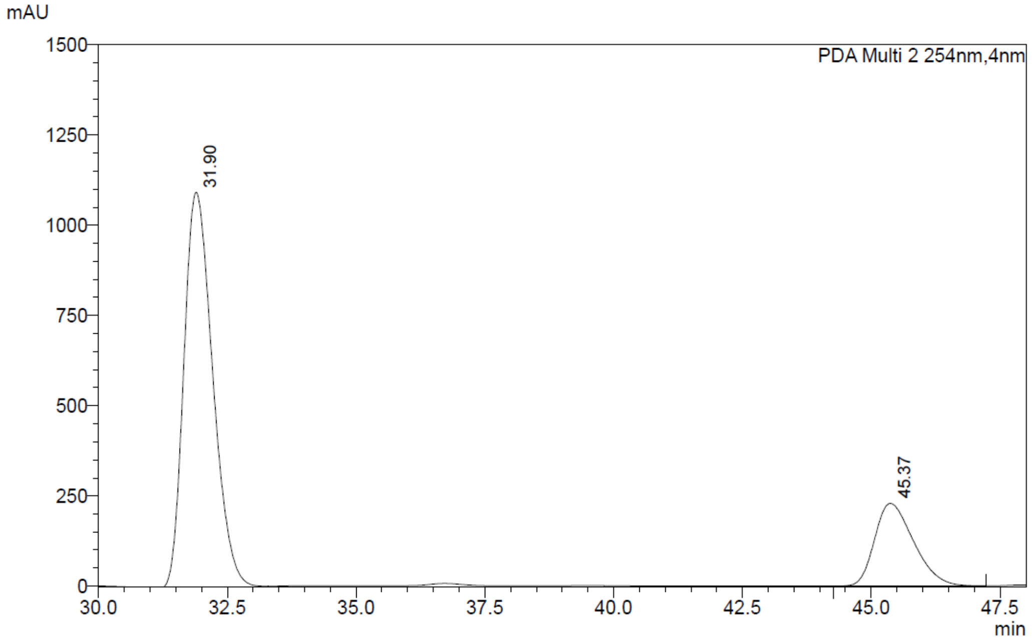

Peak Table

PDA Ch2 254nm

Peak#	Ret. Time	Area	Area%
1	31.90	43027961	78.00
2	45.37	12135252	22.00
Total		55163214	100.00

HPLC traces of compound 5k

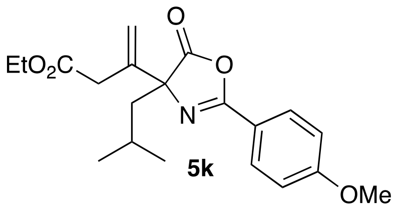

5k

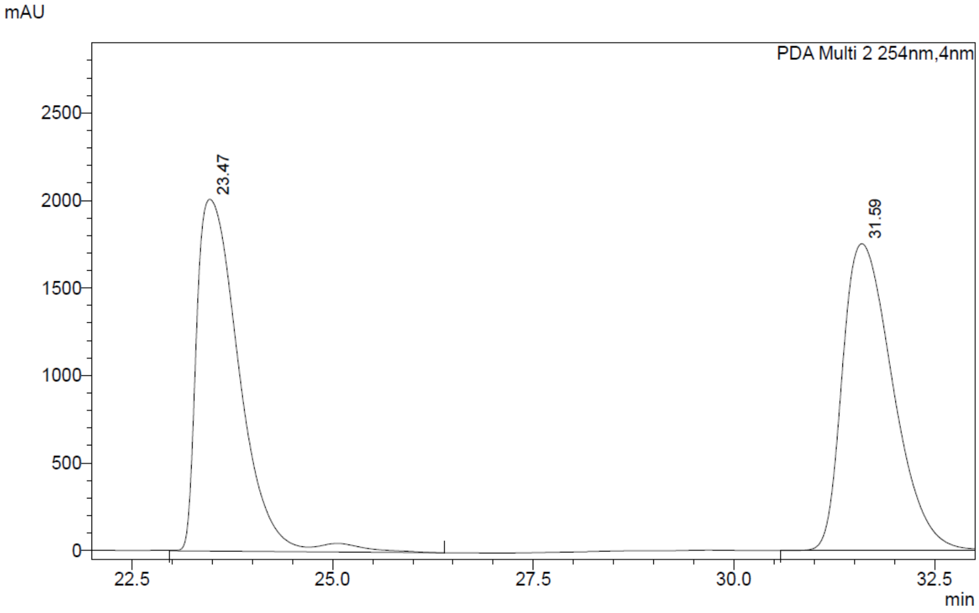

Peak Table

PDA Ch2 254nm

Peak#	Ret. Time	Area	Area%
1	23.47	74530914	49.30
2	31.59	76659495	50.70
Total		151190409	100.00

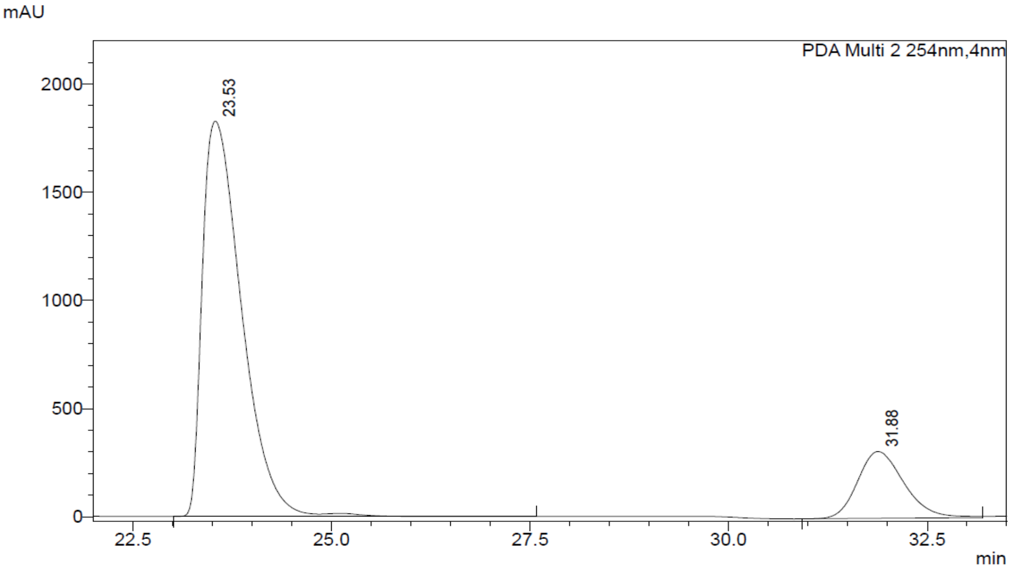

Peak Table

PDA Ch2 254nm

Peak#	Ret. Time	Area	Area%
1	23.53	63048120	83.36
2	31.88	12583478	16.64
Total		75631598	100.00

HPLC traces of compound 5l

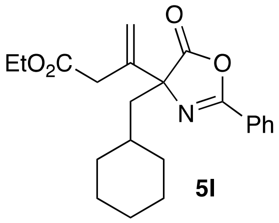

5l

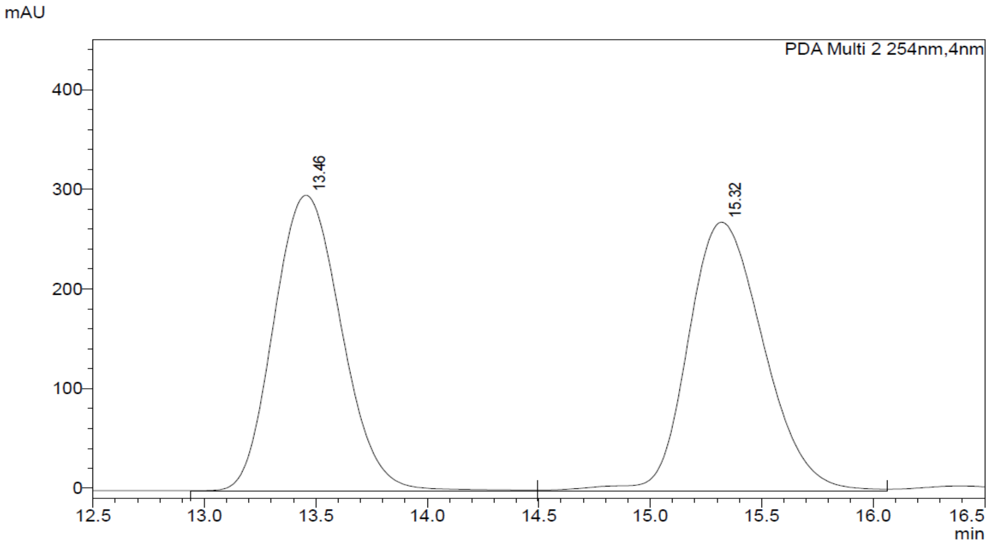

Peak Table

PDA Ch2 254nm

Peak#	Ret. Time	Area	Area%
1	13.46	6258547	49.59
2	15.32	6362149	50.41
Total		12620696	100.00

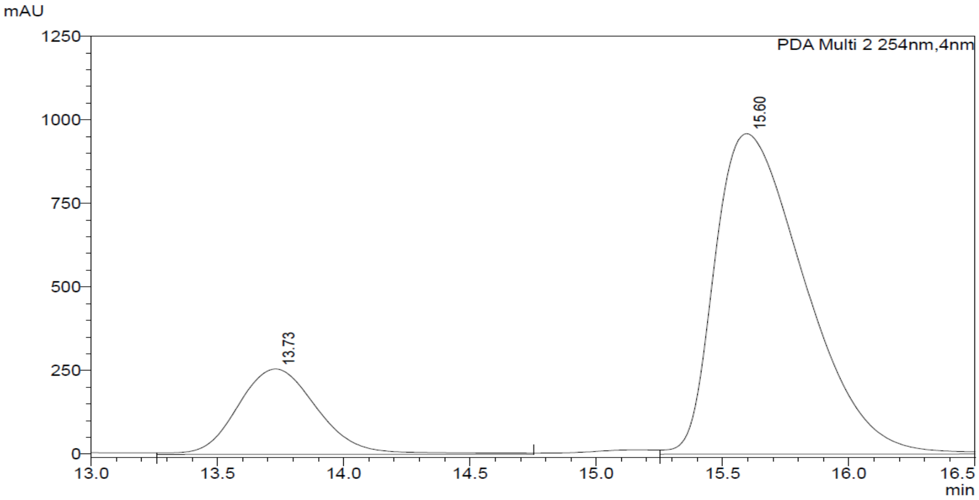

Peak Table

PDA Ch2 254nm

Peak#	Ret. Time	Area	Area%
1	13.73	5681974	19.09
2	15.60	24075662	80.91
Total		29757636	100.00

HPLC traces of compound 5m

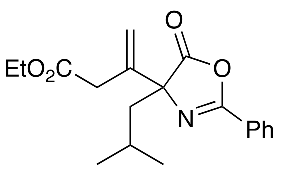

5m

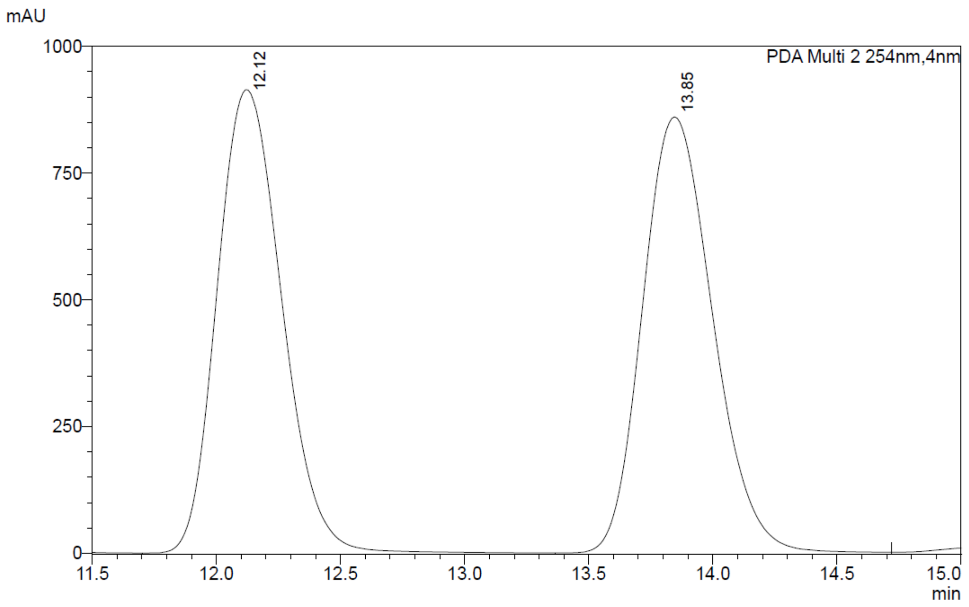

Peak Table

PDA Ch2 254nm

Peak#	Ret. Time	Area	Area%
1	12.12	16858574	49.63
2	13.85	17106534	50.37
Total		33965107	100.00

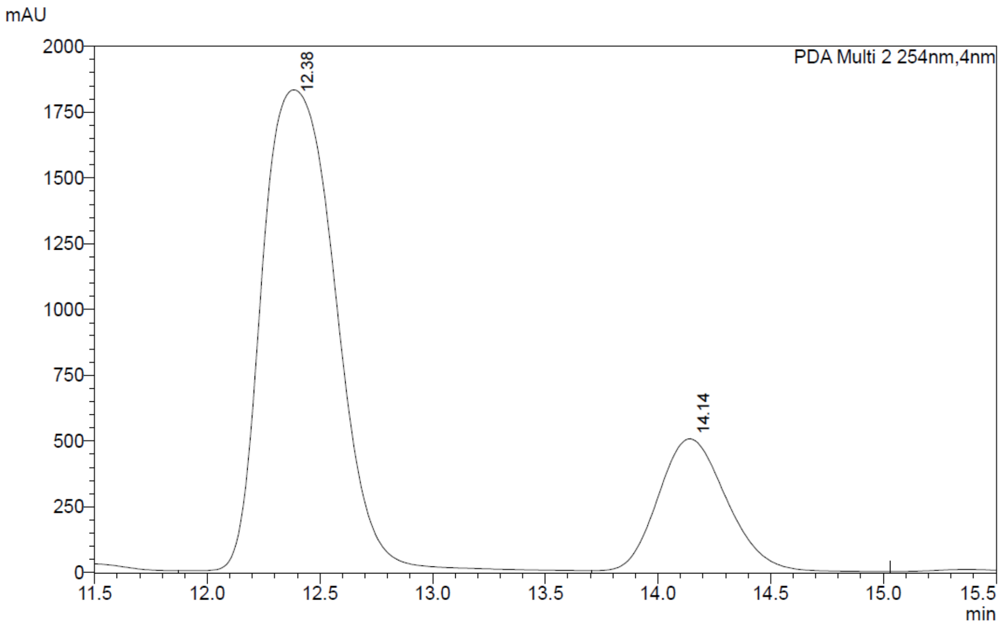

Peak Table

PDA Ch2 254nm

Peak#	Ret. Time	Area	Area%
1	12.38	42338909	79.09
2	14.14	11193623	20.91
Total		53532533	100.00

HPLC traces of compound 5n

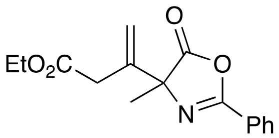

5n

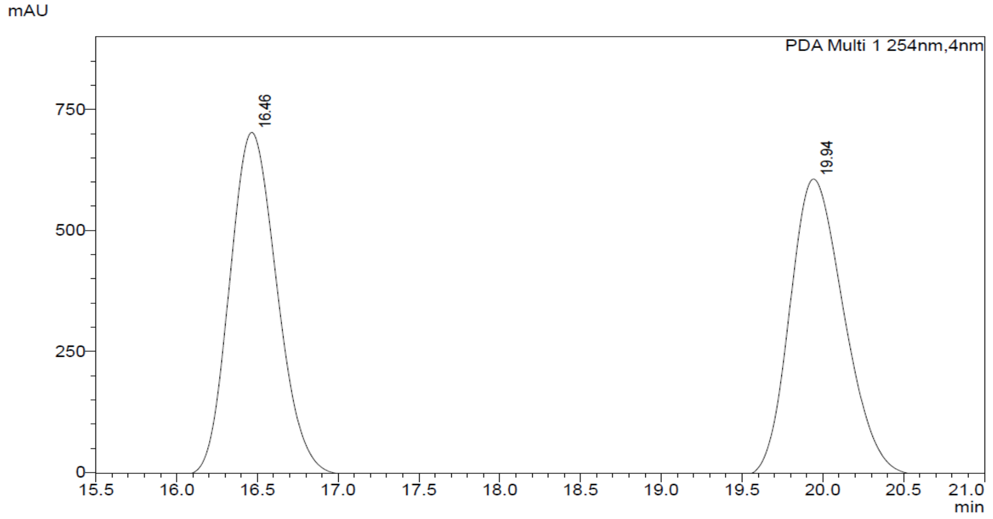

Peak Table

PDA Ch1 254nm

Peak#	Ret. Time	Area	Area%
1	16.46	14552051	49.75
2	19.94	14698394	50.25
Total		29250445	100.00

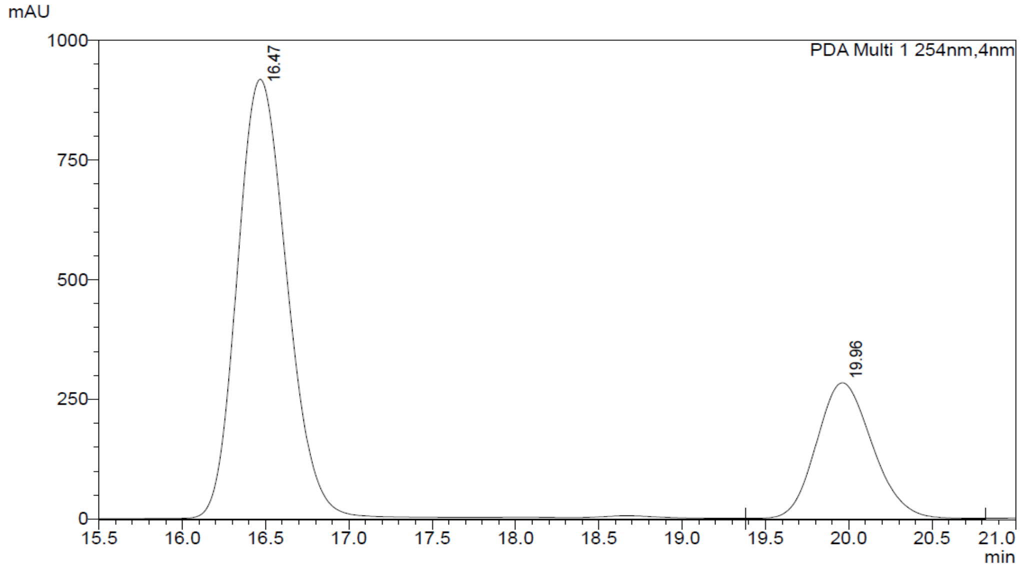

Peak Table

PDA Ch1 254nm

Peak#	Ret. Time	Area	Area%
1	16.47	19567857	74.55
2	19.96	6678516	25.45
Total		26246373	100.00

HPLC traces of compound 5o

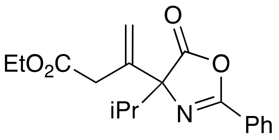

5o

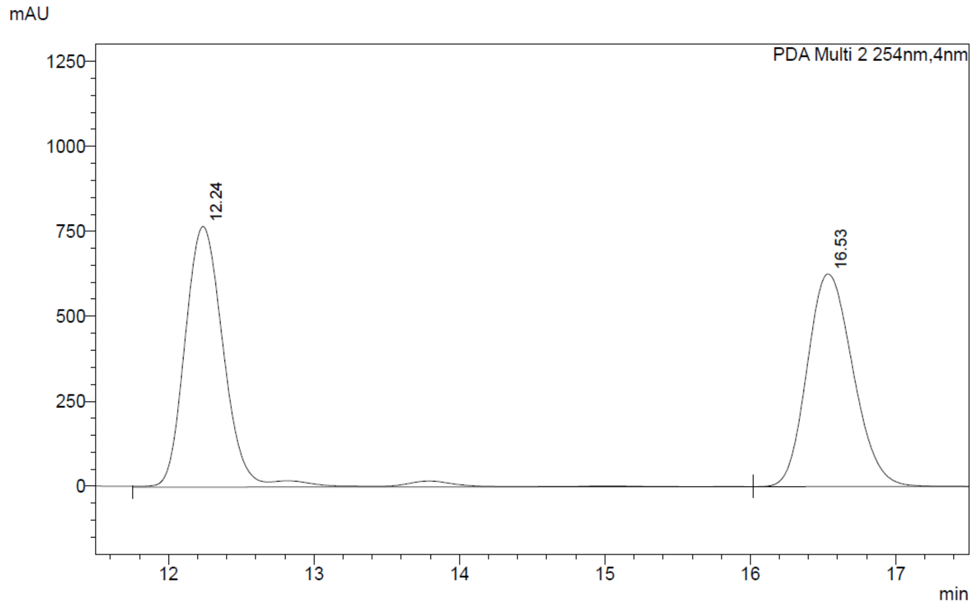

Peak Table

PDA Ch2 254nm

Peak#	Ret. Time	Area	Area%
1	12.24	14733960	51.77
2	16.53	13726796	48.23
Total		28460757	100.00

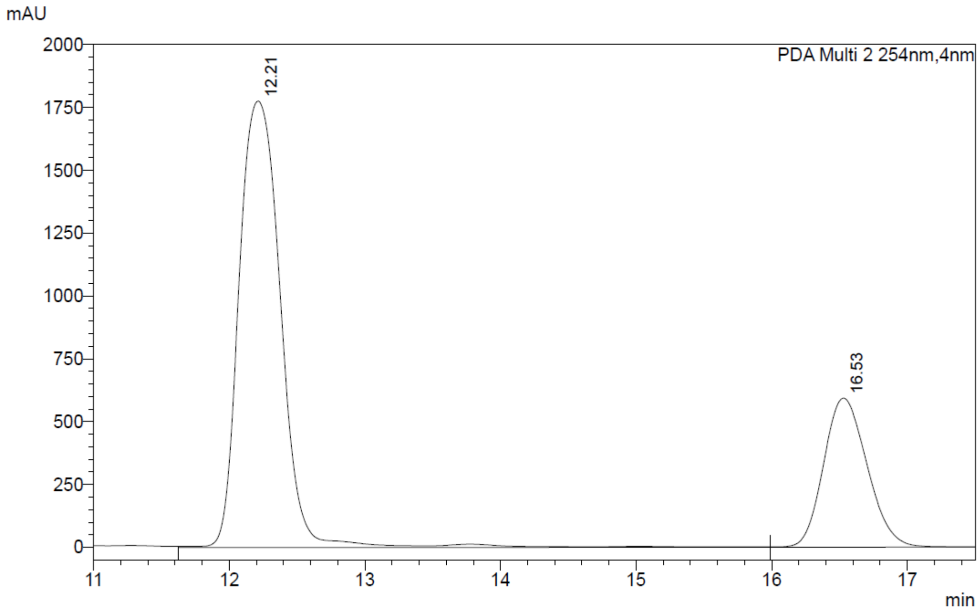

Peak Table

PDA Ch2 254nm

Peak#	Ret. Time	Area	Area%
1	12.21	38245981	74.18
2	16.53	13315363	25.82
Total		51561344	100.00

HPLC traces of compound 5p

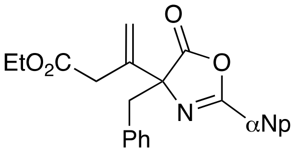

5p

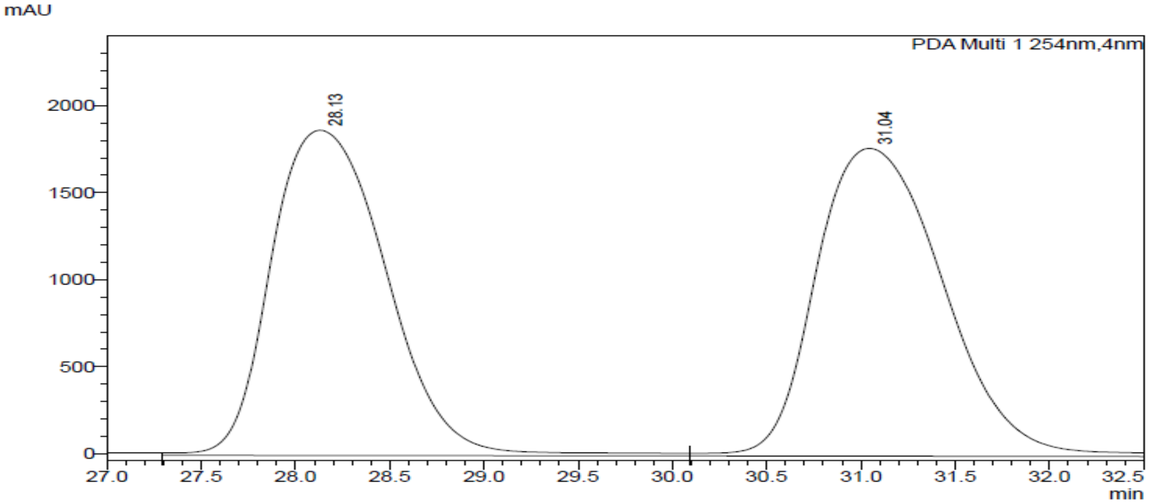

Peak Table

PDA Ch1 254nm

Peak#	Ret. Time	Area	Area%
1	28.13	79700929	46.66
2	31.04	91121437	53.34
Total		170822366	100.00

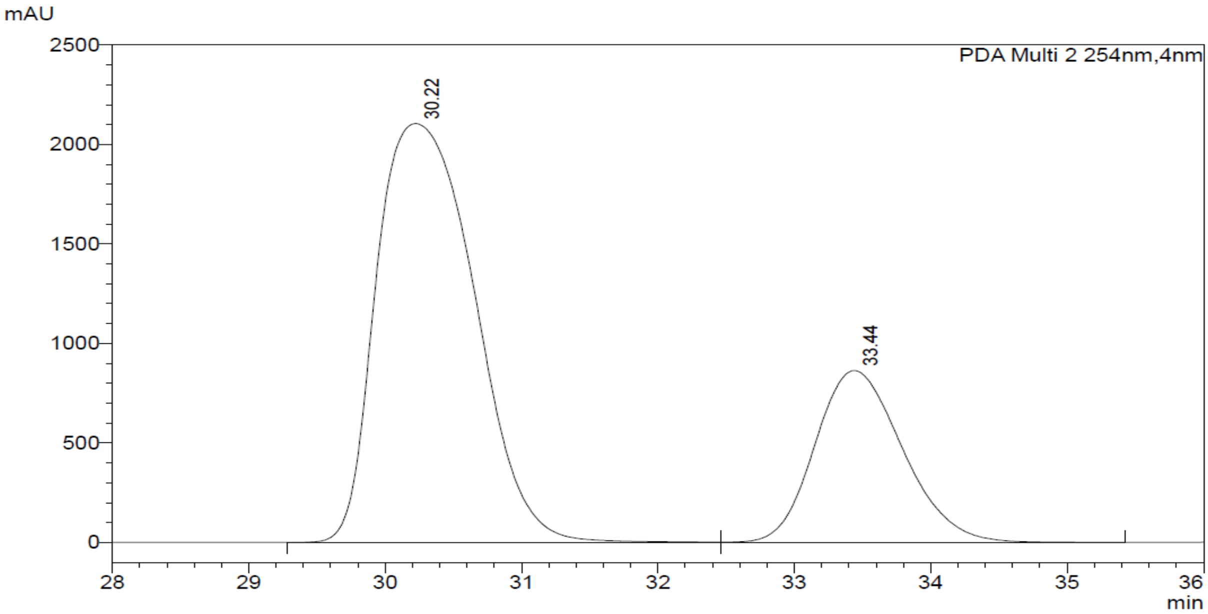

Peak Table

PDA Ch2 254nm

Peak#	Ret. Time	Area	Area%
1	30.22	105150834	73.15
2	33.44	38586996	26.85
Total		143737830	100.00
